# Insulin-degrading enzyme (IDE) as a modulator of microglial phenotypes in the context of Alzheimer’s disease and brain aging

**DOI:** 10.1186/s12974-023-02914-7

**Published:** 2023-10-11

**Authors:** Miriam Corraliza-Gomez, Teresa Bermejo, Jingtao Lilue, Noelia Rodriguez-Iglesias, Jorge Valero, Irene Cozar-Castellano, Eduardo Arranz, Diego Sanchez, Maria Dolores Ganfornina

**Affiliations:** 1 Instituto de Biomedicina y Genética Molecular, Excellence Unit, University of Valladolid-CSIC, Valladolid, Spain; 2 Instituto Gulbenkian de Ciência, Oeiras, Portugal; 3 Achucarro Basque Center for Neuroscience, Science Park of the UPV/EHU, Leioa, Spain; 4 Department of Neurosciences, University of the Basque Country, Leioa, Spain; 5 Institute of Neuroscience of Castilla y León–INCyL, University of Salamanca, Salamanca, Spain; 6 Institute for Biomedical Research of Salamanca, Salamanca, Spain; 7 Centro de Investigación Biomédica en Red de Diabetes y Enfermedades Metabólicas Asociadas (CIBERDEM), Madrid, Spain

**Keywords:** Insulin-degrading enzyme, Microglia, Myelin phagocytosis, Amyloid-beta endocytosis, Inflammation, Oxidative stress, Microglial proliferation, Cytokine secretion

## Abstract

The insulin-degrading enzyme (IDE) is an evolutionarily conserved zinc-dependent metallopeptidase highly expressed in the brain, where its specific functions remain poorly understood. Besides insulin, IDE is able to cleave many substrates in vitro, including amyloid beta peptides, making this enzyme a candidate pathophysiological link between Alzheimer's disease (AD) and type 2 diabetes (T2D). These antecedents led us to address the impact of IDE absence in hippocampus and olfactory bulb. A specific induction of microgliosis was found in the hippocampus of IDE knockout (IDE-KO) mice, without any effects in neither hippocampal volume nor astrogliosis. Performance on hippocampal-dependent memory tests is influenced by IDE gene dose in 12-month-old mice. Furthermore, a comprehensive characterization of the impact of IDE haploinsufficiency and total deletion in metabolic, behavioral, and molecular parameters in the olfactory bulb, a site of high insulin receptor levels, reveals an unambiguous barcode for IDE-KO mice at that age. Using wildtype and IDE-KO primary microglial cultures, we performed a functional analysis at the cellular level. IDE absence alters microglial responses to environmental signals, resulting in impaired modulation of phenotypic states, with only transitory effects on amyloid-β management. Collectively, our results reveal previously unknown physiological functions for IDE in microglia that, due to cell-compartment topological reasons, cannot be explained by its enzymatic activity, but instead modulate their multidimensional response to various damaging conditions relevant to aging and AD conditions.

**Supplementary Information:**

The online version contains supplementary material available at 10.1186/s12974-023-02914-7.

## Background

Alzheimer’s disease (AD) is a progressive neurodegenerative disorder and the most common cause of dementia, comprising one of the greatest health-care challenges of the twenty-first century. The main pathognomonic traits of AD are two aberrant structures, senile plaques and neurofibrillary tangles, composed by amyloid-beta (Aβ) peptides and hyperphosphorylated tau proteins, respectively [1]. A hypothesis for AD pathogenesis proposes that the onset of sporadic AD results from the collective effects of multiple factors [2], including neuronal loss, neuroinflammation, oxidative stress, white matter degeneration and impairments in brain metabolism [3]. Neuroinflammation is a complex process, mainly conducted by microglia and astrocytes in the central nervous system (CNS), and sought to play an important defensive role against pathogens, toxins and other neurodegeneration inducers [4]. Microglia are immunocompetent cells that maintain CNS homeostasis by constantly surveying their environment, acting as professional phagocytes and orchestrators of the innate immune response to protect the brain against pathogenic factors [5]. Astrocytes also maintain CNS homeostasis by transporting ions, removing neurotransmitters and releasing scavengers of reactive oxidative species [6]. Microglia are the first brain cells that respond to inflammatory stimuli and, when the situation is resolved, these cells counteract inflammation, driving astrocytes to an anti-inflammatory profile [7]. However, if the situation is not resolved, or under chronic inflammation, microglia proliferate and release inflammatory cytokines into the brain milieu, where they are sensed by astrocytes as inflammation inducers, triggering a reactive loop [8]. To accomplish their functions, microglia are highly dynamic and plastic cells, adopting many diverse and context-dependent phenotypes or functional states [9].

The term “diabetes mellitus” (DM) describes a group of chronic metabolic disorders characterized by the presence of hyperglycemia in the absence of treatment [10]. Type 1 diabetes (T1D) is an autoimmune disease that causes specific destruction of pancreatic β-cells, leading to low or no insulin secretion into the bloodstream [11], while type 2 diabetes (T2D) is characterized by an insufficient response to insulin by tissues of the body, known as insulin resistance [12]. In 2005, de la Monte and Wands [13] proposed the concept of "type 3 diabetes" which postulates that AD is a neuroendocrine disorder associated with chronic deficits in insulin signaling that shares features with both T1D and T2D (insulin deficiency and resistance, respectively). The synergy between AD and T2D is supported at the preclinical level, with studies on both AD-prediabetes (APP/PS1 mice exposed to high fat diet) and AD-T2D models (APP/PS1xdb/db double transgenic mice). These murine models reveal an initial exacerbation of AD brain pathology in prediabetic mice, significantly worsened in fully established diabetes [14]. In addition, cross-sectional studies in humans show such association in a bidirectional manner: T2D is over-represented in AD patients in comparison with controls [15–17] and, on the other hand, large population-based longitudinal studies show that T2D may significantly increase the risk of AD [18–21].

The insulin-degrading enzyme (IDE) is an evolutionarily conserved zinc-dependent metalloendopeptidase [22], named after its ability to strongly bind and degrade insulin in tissue extracts [23]. Besides insulin, many other different short peptides with similar conformation have been reported to be degraded by IDE in vitro [24]. The discovery of IDE as an Aβ degrading enzyme [25] posited this enzyme, highly expressed in the brain [26], as a candidate pathophysiological link between AD and T2D. In fact, there is compelling evidence that the gene region around IDE might be genetically associated with both AD [27–35] and T2D [36–39]. Preclinical studies reported increased Aβ peptide accumulation in the brain of IDE-deficient mice at 3 months of age [40] and a clear diabetic phenotype in those mice at 6 months [41]. However, no data is available for older animals, undergoing natural aging-related neurodegeneration, nor the specific mechanisms by which IDE absence might alter brain function. A widely used hypothesis postulates that high insulin concentrations would decrease IDE-mediated Aβ degradation due to competitive inhibition [42]. However, brain insulin levels are much lower than the Michaelis constant (Km) of insulin for IDE, and thus unlikely to competitively inhibit Aβ degradation [43]. Furthermore, we recently described that in microglia IDE associates with membranes by their cytosolic side and is exported outside the cell inside extracellular vesicles [22]. On the contrary, both Aβ and insulin would be outside these vesicles in the extracellular compartment or inside endocytic vesicles within the cell. In both situations IDE and its potential substrates are in separate compartments, posing an important question about the feasibility of a proteolytic activity of IDE on insulin and Aβ in vivo.

Our purpose in this work was to initiate a much-needed search for IDE functions in the brain that is not conditioned by the pre-assumption that the consequences of IDE functions are dependent on its Aβ or insulin proteolytic activity. We first found that IDE loss causes a significant deviation from Mendelian proportion due to prenatal lethality. Thus the experimental cohort includes adults surviving IDE-dependent developmental deficits. Our histological analysis of the hippocampus reveals that IDE absence specifically affects microglial phenotypes. We studied a cohort of 12-month-old wild-type (WT), heterozygous (IDE-HET), and knockout (IDE-KO) mice for *Ide* gene and found that performance on hippocampal-dependent memory tests is influenced by IDE gene dose. In contrast, IDE dose does not influence basal insulin-signaling in the olfactory bulb, the brain region with the highest amount of insulin receptors [44, 45]. Still, a multivariate analysis of metabolic, behavioral, and molecular parameters was able to detect an unambiguous "barcode" for IDE-KO mice, disclosing IDE relationship with brain-relevant variables. Our work focused on microglia as master regulators of the neuroinflammatory response, revealing that IDE absence alters different aspects of microglial physiology. Transcriptomic profiling revealed immune responses and regulation of cell activation and division as pathways most affected by IDE loss in microglial cells. IDE loss results in differential cytokine responses to stimuli relevant to aging and neurodegenerative conditions, including a sex-dependent response to Aβ. Also, IDE-KO microglia decrease their proliferation and delay their response to the hematopoietic growth factor M-CSF. While IDE does alter, also in a sex-dependent way, the process of myelin phagocytosis, no significant changes in Aβ internalization or clearance were found, with only minor and transitory effects observed. Collectively, our results reveal previously unknown functions of IDE as a modulator of microglial phenotypes that cannot be directly attributed to its enzymatic activity, and instead place their neuroimmune response at the center for future research.

## Material and methods

### Experimental animals

#### Mice genotyping and maintenance

The IDE-knockout (IDE-KO) mouse [Ide^tm1a(EUCOMM)Wtsi^, Mouse Genome Informatics identifier MGI:4,431,946 [41]], kindly provided by Dr. M.A. Leissring (University of California-Irvine, USA), was backcrossed with WT mice on a C57BL/6J background (Charles River, France), and the colony was maintained by crossing heterozygous animals. Mouse genotypes were evaluated by PCR, as previously described [22], and confirmed by protein expression by immunoblot [46] and Additional file 1: Fig. S1.2). Mice were maintained in positive pressure-ventilated racks at 25 ± 1 °C with 12 h light/dark cycle, fed ad libitum with standard rodent pellet diet (Harlan Inc., USA), and allowed free access to filtered and UV-irradiated water. Experimental procedures were approved by the University of Valladolid Animal Care and Use Committee and the competent authorities at Junta de Castilla y León (project #8,702,359), following the regulations of the Care and the Use of Mammals in Research (European Commission Directive 86/609/CEE, Spanish Royal Decree ECC/566/2015).

#### Behavioral experiments

Behavioral studies were performed in 12-month-old mice using a MIR-100 infrared digital camera and the Activity Monitor acquisition and analysis program (Med Associates, USA). All procedures were in accordance with institutional guidelines for animal care and administration. Behavioral tests were modified from previously described methods [47, 48]. An open field squared box constructed from wood (45 × 45 × 30 cm) was used. Two of the walls of the box in opposite positions had two different figures (a circle and a star) to allow spatial orientation. The procedure included 4 phases, performed on two consecutive days: an open field test (OF) and habituation on the first day, an object familiarization trial, an object location test (OLT), and a novel object recognition test (NORT) on the second day. All the experimental sessions for behavioral tests were performed between 8 and 10 am.

##### Open field test and habituation

Mice were allowed to freely explore the arena for 5 min. Locomotor behavior, ambulatory distance, stereotypic counts, and a ratio center/periphery were automatically analyzed for each mouse. This open field session was followed by two new training sessions in which mice were habituated to the empty arena for 6 min/session, with a 20-min resting period between each session.

##### Object recognition tests

All objects were made from the same material (plastic Lego^®^ parts) but with different shapes, heights, and colors. The objects were fixed on the floor of the arena to ensure that the mice could not displace them. To prevent the existence of olfactory cues between mice, the entire box and objects were thoroughly cleaned with 70% ethanol solution after each session.

- Object familiarization trial: Two identical objects were placed at parallel positions within the box at the same distance from the nearest corner (6 cm). Mice were allowed to explore the identical objects for 10 min, and then returned to their home cages for 40 min.
- Object location test (OLT): One of the objects was moved to an opposite position with respect to the other object, being both objects placed in diagonal positions within the box at the same distance from the nearest corner. This session lasted 10 min and was followed by a 40-min resting period.
- Novel object recognition test (NORT): For the second testing trial, the familiar object that had not been moved during the OLT was replaced by a new object, and mice were placed back in the same arena to carry out a 10-min testing session.

##### Behavioral tests analysis

An exploratory event was considered when the animals directed their nose at a distance within 2 cm of the object and/or touched the object with the nose, while turning around or sitting near the object without looking at it was not considered as an exploration [49]. An independent researcher, blind to mouse genotype, analyzed the videos and annotated every time that the mouse explored any of the two objects. A Discrimination Index (D.I.) was calculated as follows:

$$D.I.\left(OLT\right)=\frac{Novel\;location\;explorations-Familiar\;location\;explorations}{Total\;explorations},$$

$$D.I.\left(NORT\right)=\frac{Novel\;object\;explorations-Familiar\;object\;explorations}{Total\;explorations}.$$

A positive value of the discrimination index indicates more times investigating the novel location or object, while a discrimination index of zero or lower indicates no preference for the novelty.

#### Metabolic parameters

Body weight was monitored by using a digital weight scale (Ohaus, Switzerland). Blood glucose levels after 6-h fasting (10 am to 4 pm) were analyzed by collecting blood from the tail vein and measuring with a Contour Next glucometer (Bayer, Germany). For the preparation of plasma, blood was collected from the tail vein into Microvette^®^ capillary tubes precoated with potassium-EDTA (Sarstedt, Germany) and centrifuged (3300*g,* 10 min, 4 °C). To determine plasma insulin levels, we used the Mouse Ultrasensitive Insulin ELISA (CrystalChem, USA) assay following the manufacturer’s instructions.

To evaluate alterations in glucose homeostasis, we performed an intraperitoneal glucose tolerance test (i.p.GTT). Mice were fasted for 16 h and then injected intraperitoneally with 2 g glucose/kg body weight. Blood glucose levels were quantified immediately before and 15, 30, 60, and 120 min after the glucose challenge.

#### Molecular biology studies in the olfactory bulb

Mouse brains were quickly removed following decapitation after CO_2_ anesthesia and hemispheres were separated. Tissues were immediately frozen by immersion in isopentane and stored at − 80 °C. Olfactory bulb tissues, which had an average weight of 30 mg, were mechanically homogenized in 5 volumes of homogenization buffer [0.5% sodium deoxycholate, 10 mM HEPES pH 7.6, 100 mM KCl, 1 mM EDTA pH 8.0, 1% NP-40, 0.1% SDS, 10% glycerol, 1 mM DTT, 1X Complete protease inhibitor cocktail, 1 mM PMSF, 1X Phosphatase inhibitor cocktail 2, 1X Phosphatase inhibitor cocktail 3 (Sigma-Aldrich, USA)] and incubated on ice for 30 min. Samples were centrifuged (16,400*g*, 10 min, 4 °C) to pellet insolubilized tissue, and supernatants were used in subsequent steps. Protein concentration from each sample was estimated by using the Micro BCA™ Protein Assay Kit (Thermo Scientific, USA). Protein extracts (50 μg) were mixed with protein sample buffer [63 mM Tris–HCl pH 6.8, 10% (v/v) glycerol, 2% (w/v) SDS, 100 mM DTT, 0.05% (w/v) bromophenol blue], heated (70 °C, 5 min) and analyzed by immunoblotting.

#### Histological studies in the hippocampus

Twelve-month-old mice were intraperitoneally anesthetized with a mixture of ketamine and xylazine (100 mg/kg and 10 mg/kg, respectively). Animals were perfused with warm PBS (constant flow rate 1.5 ml/min), followed by 4% paraformaldehyde in PBS (1.5 volumes of fixative per body weight). Brains were collected, post-fixed by immersion in 4% PFA in PBS for 3 h, and stored in 30% sucrose, 30% ethylene glycol in PBS at − 20 °C until analysis. Six series of 50-µm-thick sagittal sections of mouse brains were cut using a LeicaVT1200S vibrating blade microtome (Leica Microsystems, Germany). Fluorescent immunostaining was performed following standard procedures. Free-floating vibratome sections were blocked in permeabilization solution (0.2% Triton X-100, 3% BSA in PBS; all from Sigma-Aldrich, USA) for 1 h at room temperature (RT), and then incubated overnight with primary antibodies [goat anti-GFAP (1:1000; #AB53554, Merck-Millipore, Germany) and rabbit anti-Iba1 (1:1000; #016-20001, Wako Chemicals, Germany)] diluted in permeabilization solution at 4 °C. Then, brain sections were thoroughly washed with PBS and subsequently incubated with fluorochrome-conjugated secondary antibodies [donkey anti-goat-Alexa Rhodamine Red (1:500; 705-295-147, Jackson ImmunoResearch, UK) and donkey anti-rabbit-Alexa 488 (1:500; 711-545-152, Jackson ImmunoResearch, UK)] and DAPI (5 µg/ml; Sigma-Aldrich, USA) diluted in the permeabilization solution for 2 h at RT. After washing with PBS, the sections were mounted on glass slides with Dako Cytomation Fluorescent Mounting Medium (Agilent Technologies, Spain). Samples were imaged using an automated digital slide scanner (Pannoramic Midi II; 3D Histech Ltd, Hungary) equipped with a Plan-Apochromat 20 × objective (NA: 0.8). The final resolution of our images for analysis was 0.2031 μm^2^/pixel. At least 5 slices/mouse were analyzed. We collected images from sections containing the septal hippocampus (from medial/lateral coordinates 0.2 to 1.6 mm). To obtain volumetric measurements, we used Cavalieri's method on images from one out of every six sections (with an interval of 1/6), using our ImageJ macro VolumestJ [50]. To obtain the fraction of stained area (area fraction), we used our ImageJ macro Thresholderer (https://github.com/Jorvalgl/Thresholderer). We pre-processed the images to reduce the background using a difference of Gaussians (DoG) filter with a minimum sigma of 0 and a maximum sigma of 50 μm. Then, the area fractions of the selected regions containing pixels above the different intensity levels (from 0 to 255) were sequentially obtained and analyzed. For our final analysis, we used fraction area data from a representative intensity level corresponding to the specific staining.

### Immunoblotting

Samples were run in gels (5–15% acrylamide) under denaturing conditions (0.5% SDS, 25 mM DTT). Gel electrophoresis was carried out at constant voltage (100 V) in electrophoresis buffer [25 mM Tris, 190 mM glycine, 1% SDS, pH 8.3]. Proteins were then transferred to PVDF membranes (Immobilon-P, Merck-Millipore, Germany) using an immersion system at a constant current (400 mA) for 90 min. Transferred membranes were incubated for 1 h at RT with blocking solution (0.5 M Tris-base pH 7.5, 1.5 M NaCl, 0.05% Tween-20, with either 5% non-fat dry milk or 3% BSA). Primary antibodies, incubated overnight at 4 °C in blocking solution, included: anti-phospho-insulin receptor-β (Tyr1150/1151), anti-insulin receptor-β, anti-phospho-Akt (Ser473), anti-Akt (all made in rabbit and used at 1:1000; #3024, #3025, #9271 and #9272, respectively, from Cell Signaling, USA), rabbit serum anti-IDE (1:40,000; #AB9210, Merck-Millipore, Germany); rat anti-CD11b (1:200; DSHB, USA); mouse anti-Iba1 (1:200 = 1 μg/ml; #sc-32725), mouse anti-GFAP (1:2000 = 0.1 μg/ml; #sc-33673), goat serum anti-ApoD (1:1000 = 0.2 μg/ml; #sc-34760), mouse anti-β-amyloid (1:1000 = 0.2 μg/ml; #sc-28365) (Santa Cruz Biotechnology, USA); mouse anti-β-actin-HRP (1:200,000 = 0.015 μg/ml; #A3854, Sigma-Aldrich, USA). Afterwards, membranes were incubated with HRP-conjugated secondary antibodies (1:10,000; Jackson ImmunoResearch, USA). Membranes were developed with enhanced-chemiluminescence reagents (ECL; Merck-Millipore, Germany), and the signal was visualized with a digital camera (VersaDoc; BioRad, Spain). The integrated optical density of the immunoreactive protein bands was measured in images taken within the linear range of the camera, avoiding signal saturation and using the Quantity One 1-D Analysis Software (BioRad, Spain).

### Primary microglial cultures and treatments

Gender assessment of the pups was performed as previously described [51]. Primary microglial cultures were prepared from individual postnatal day 0 male and female mice, following the “mild trypsinization method” described by Saura and colleagues [52] with minor modifications [22]. Mixed glial cultures were seeded at a density of 62,500 cells/cm^2^ on different formats (1.13–78.5 cm^2^), depending on the experiment. After 20 days in vitro, microglia were separated from astrocytes and incubated in astrocyte-derived conditioned media supplemented with macrophage colony-stimulating factor [M-CSF, 20 ng/ml, (Peprotech, Germany)] for 3–5 days to obtain “resting microglia”. Only pure microglial cultures were used throughout the experiments, discarding any culture well or flask containing residual astrocytes after the step of mild trypsinization (Additional file 3: Fig. S3.1). Experimental treatments were performed in RPMI 1640 medium under no-serum conditions, except for the proliferation assays, which were performed in DMEM-12 supplemented with 10% FBS. Treatments assessed included: LPS [100–1000 ng/ml, 8–24 h (lipopolysaccharide; Sigma-Aldrich, USA)], Paraquat [25–500 μM, 8–24 h (PQ; Sigma-Aldrich, USA)], IL-4 + IL-13 [20 ng/ml and 50 ng/ml, respectively, 18 h (PeproTech, Germany)], myelin-DiI [20 μg/ml, 3–24 h, Sigma-Aldrich, USA; prepared as described by [53]], Aβ oligomers [1–4 μM, 8–18 h, [Bachem, Switzerland]), and FAM-Aβ oligomers [1 μM, 0.5–24 h (Bachem, Switzerland)].

### RNA-sequencing and quantitative PCR validations

WT and IDE-KO primary microglial cultures were prepared as described above, with each sample coming from an individual male mouse, and seeded in Petri dishes (78.5 cm^2^). RNAs were extracted using the RNeasy kit (Qiagen, Spain), following the manufacturer's instructions. The purity and yield of total RNA were assessed in a NanoDrop ND-1000 spectrophotometer (Thermo Scientific, USA). RNA quality was evaluated by microcapillary electrophoresis in a TapeStation System (Agilent Technologies, Germany). Only RNA samples with A260/A280 > 2.0 and RNA Integrity Number (RIN) > 9.9 were used. Three samples per genotype were processed. Libraries were prepared using the QuantSeq 3′ mRNA-Seq Library Prep Kit for Illumina (Lexogen, Austria), according to the protocols recommended by the manufacturer. Sequencing was performed using NextSeq500 High Output Kit 75 cycles (Illumina, USA), single-ended, 20 million reads per sample, in a HiSeq2000 platform (Illumina, USA). Quality control was performed using FastQC. Samples were aligned using STAR software [54], and counts obtained with featureCounts [55] using the mouse genome mm10 GRCm38. The quality of sequence alignments to the reference genome was assessed using Qualimap [56]. Differential gene expression analysis was conducted using the DeSeq2 package in R [57]. The principal component analysis (PCA) of the triplicates suggested discarding one sample of each genotype because they were clear outliers. Therefore, the analysis was repeated with duplicates per genotype. Common gene names were retrieved with the biomaRt R package [58] based on Ensembl gene IDs. Gene Ontology Enrichment Analysis of the differentially expressed genes (DEGs, with a corrected *p*-value < 0.05) was performed using gprolifer2 [59].

To validate RNA-seq results, we selected four transcripts to assay their transcription with quantitative real-time RT-PCR (RT-qPCR) in WT and IDE-KO using independent microglia RNA samples. Total RNA was treated with DNase and reverse transcribed with PrimeScript™ (Takara Bio Inc., Otsu, Japan), using Oligo-dT primers and random hexamers. cDNAs were used as template for amplification using SybrGreen (SYBR^®^ Premix Ex Taq™kit, Takara). Primers used for RT-qPCR are: Slfn1-Forward: AATCCAAGTGCTGAGAAGGAC; Slfn1-Reverse: TTCATTTTCCAGCAGTCTTGC; Lcn2-Forward: TGTACAGCACCATCTATGAGC; Lcn2-Reverse: ATGGCGAACTGGTTGTAGTC; Fcna-Forward: CAAAGTTGCCCTGGCTTTC; Fcna-Reverse: GTCTCCCAGCTCCTTTTCAC; Timd4-Forward: GTCCGCCTTCACTACAGAAT; Timd4-Reverse: CTGCAAAGACTCACTTGTTGT; Rpl18-Forward: TTCCGTCTTTCCGGACCT; Rpl18-Reverse: TCGGCTCATGAACAACCTCT. The mRNA transcription levels were evaluated by the ΔΔCT method [60]. Transcripts were normalized to the Rpl18 gene.

### Live/dead viability assay

To determine the viability of primary microglial populations (seeded in 6-well plates, 9.61 cm^2^/well), we used the “LIVE/DEAD™ Fixable Near-IR Dead Cell Stain Kit” (Invitrogen, USA) following the manufacturer's instructions. A staining control was routinely prepared by dividing one sample into two aliquots, one of which was heated at 99 °C for 5 min to kill the cells, and after cooling, both aliquots were rejoined (50% live/dead). Live/Dead dye (0.5 µl) was added to samples, mixed by vortex, incubated for 1 min at RT, and centrifuged (400*g*, 5 min, 4 °C) to wash away the excess dye. Cell pellets were resuspended in 500 µl of FACS buffer (2% FBS, 3 mM NaN_3_, 1 mM EDTA in PBS) and immediately analyzed in a Gallios flow cytometer (Beckman Coulter, Spain), exciting the cells with a red laser (633 nm) and collecting fluorescence intensity in the 755 LP channel (> 755 nm). Data were processed using Kaluza software (Beckman Coulter, Spain). At least 10,000 cells per condition were analyzed.

### Proliferation assay

Primary microglial cells seeded on 12-mm-diameter coverslips (1.13 cm^2^) were allowed to attach to the culture surface for 24 h. Then, Click-iT EdU assays (Invitrogen, USA) were performed in complete medium (DMEM-F12 + 10% FBS + 1% L-glutamine + 1% P/S). Cells were treated with 10 μM EdU solution for 24–30 h, either in control (complete medium) or proliferative (complete medium supplemented with 50 μg/ml M-CSF) conditions. Afterwards, cells were washed with PBS, fixed with 4% paraformaldehyde in PBS for 15 min at RT, and processed following the manufacturer’s instructions. Briefly, cells were permeabilized with 0.1% Triton X-100 in PBS for 10 min, followed by incubation with Click-iT Plus reaction cocktail for 30 min at RT. After PBS washes, cell nuclei were stained with Hoechst 33,342 solution (5 μg/ml, Invitrogen, USA). Samples were mounted with Vectashield (Vector Laboratories, USA) and visualized with an Eclipse 90i fluorescence microscope equipped with a DS-Ri1 digital CCD camera (Nikon, The Netherlands). At least 12 images per condition were acquired using a 20X objective under the same conditions of illumination, diaphragm and condenser adjustments, exposure time, background correction, and color levels. Images were analyzed using FIJI software and results were expressed as a percentage of proliferating cells:

$$\%Proliferating\;cells=\frac{EdU\left(+\right)nuclei}{Total\;nuclei}x100.$$

### Cytokine profiling: ELISA and Luminex assays

Primary microglial cultures, seeded in 6-well plates, were isolated as described above and incubated for 5 days in astrocyte-produced conditioned media supplemented with M-CSF (20 ng/ml). Then, cells were serum-starved for 3 h and exposed to different stimuli in RPMI 1640 medium without FBS for 18 h. Conditions assessed were: unstimulated cells with carrier; 100 ng/ml LPS; 20 ng/ml IL-4 + 50 ng/ml IL-13; 25 μM PQ and 1 μM Aβ oligomers. Each condition was performed in sextuplicate (samples from male and female microglial cells, triplicates for each sex). After the incubation period, culture media were collected, centrifuged (1000*g*, 10 min, 4 °C) to pellet cell debris, and stored at − 20 °C. We used a customized Luminex kit (R&D System, USA) to perform a 5-plex assay to measure IL-1β, IL-6, TNF-α , IL-4, and IL-10, following the manufacturer's instructions. We also analyzed a negative control sample containing fresh RPMI medium supplemented with IL-4 + IL-13 to check that the IL-4 that was added as a stimulus was not detected by the assay. Samples were analyzed using a Luminex 100^®^ Analyzer (Luminex Corp, USA).

TGF-β was quantified in cell culture supernatants using the assay DuoSet ELISA Mouse TGF-β1 (R&D System, USA), following the manufacturer’s instructions. The absorbance of the samples was measured by spectrophotometry (*λ* = 450 nm) using the SOFTmax Pro microplate reader (Molecular Devices, USA).

### Myelin phagocytosis and degradation experiments

Confluent primary microglial cultures, grown in 6-well plates, were obtained as described above. Myelin isolation and labeling were performed as previously described, following a method based on discontinuous gradient centrifugation of sucrose [61]. DiI-labeled myelin was added to microglial cells (20 µg/ml) in RPMI 1640 medium. Different treatment time courses were explored: 3–18 h for phagocytosis and 3–24 h for degradation. After the incubation period, non-phagocytosed and unbound myelin was removed by washes with PBS. Cells were collected by combining trypsin/EDTA incubation (2.5 mg/ml, 15 min, 37 °C) and micropipette resuspension. After centrifugation (400*g*, 5 min, 4 °C), cells were resuspended in FACS buffer and immediately analyzed by flow cytometry. The amount of myelin phagocytosed and/or degraded was determined by measuring cellular fluorescence intensity recorded in the 575 BP30 channel (560–590 nm) after excitation with a blue laser (488 nm). At least 10,000 cells per condition were analyzed. Histograms of untreated microglia, unexposed to myelin-DiI, were used to define the fluorescence threshold value to consider events as DiI-positive microglia.

### FAM-Aß managing experiments

FITC-labeled Aβ (FAM-Aβ) oligomers were prepared as previously described [46]. Treatments with FAM-Aβ oligomers (1 µM), prepared in RPMI 1640 medium without FBS, were added to primary microglial cultures grown in 6-well plates. Different time courses of treatments were explored: 0.5–3 h for internalization and 1–24 h for degradation. After the incubation period, cells were washed with PBS to remove non-internalized FAM-Aβ. Cells were collected by trypsin/EDTA incubation (2.5 mg/ml, 15 min, 37 °C) followed by resuspension with micropipette and centrifugation (400*g*, 5 min, 4 °C). To discard the signal from FAM-Aβ externally bound to cell membranes, cells were incubated with 0.05% trypan blue stain (Lonza, Italy) in PBS (pH 7.4) for 1 min at RT to quench only extracellular signal, as previously described [62]. Then, samples were centrifuged (400*g*, 5 min, 4 °C) to wash out excess trypan blue, and cell pellets were resuspended in FACS buffer and immediately analyzed by flow cytometry [525 BP40 channel (505–545 nm), illumination with a blue laser (488 nm)]. The percentage of FAM-Aβ-positive cells, as well as the FAM-Aβ fluorescence intensity, were analyzed with Kaluza software.

### Statistical analysis

Statistical analyses were performed with SigmaPlot (Systat Software, USA) and RStudio. To check the normality of distributions, a Kolmogorov–Smirnov test was used. To analyze statistically significant differences between two sets of data, Student’s *t*-test (for parametric data) or Mann–Whitney *U* test (for non-parametric data) were applied. Comparisons between more than two groups of data were performed using ANOVAs of as many ways as factors (for parametric data) and Kruskal–Wallis test (for non-parametric data). Post hoc analyses were carried out using Bonferroni tests (parametric data) or Holm–Sidak tests (non-parametric data). For the multivariate analysis, the correlation between variables was assessed by calculating Pearson correlation coefficients. Variables with high collinearity were discarded for the dimensionality reduction by principal component analysis (PCA). A multinomial logistic regression was performed to test whether the PCA-selected variables are able to significantly differentiate individuals by their genotype. For hypothesis testing, α was set at 0.05 as a threshold for significant changes. The specific tests used for each experiment are stated in the figure legends.

## Results

### IDE loss causes deviation of Mendelian inheritance due to prenatal lethality

This study was designed to experimentally evaluate the role of IDE in 12-month-old WT, HET (heterozygous) and KO mice for the *Ide* gene, in order to assess its function in the nervous system at an age when functional deficits start accumulating [63]. Our cohort is in the age range used in many studies analyzing AD models in mice [64], and can thus be appropriate to test current hypotheses of IDE role in AD or as a link between AD and T2D.

Our first finding, while generating the experimental cohorts, is that the progeny obtained from crosses of IDE-HET mice did not follow Mendelian inheritance (*χ*^2^ = 12.6; degrees of freedom = 2; *p* = 0.002; *N* = 179 mice from 29 litters). A clear deviation from expected proportions is caused by the increase of HET to the detriment of KO mice, strongly supporting that absence of IDE causes prenatal lethality. No previous studies on this IDE-KO line reported this finding, which posits an important role for IDE during development, worth of future studies. We should therefore be aware that the IDE-KO group is composed of resilient adults surviving IDE loss.

### Microglial cells but not astroglia are altered in the hippocampus of IDE-KO mice

**Fig. 1:**
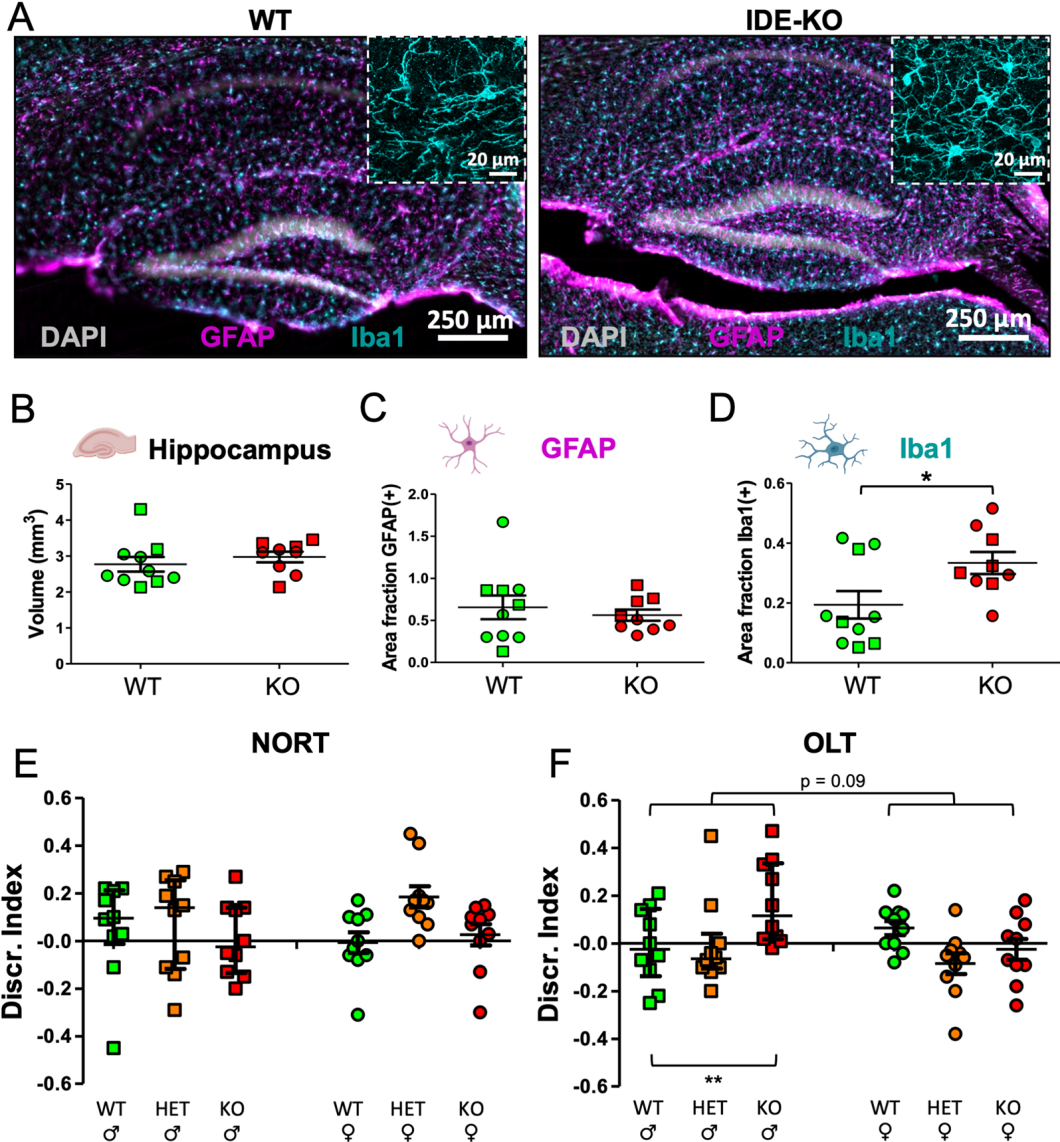
IDE absence has specific effects on hippocampal microglia, triggering microglia phenotypic modulation without affecting astrocytes. **A** Representative confocal images of immunohistochemistry experiments on 12-month-old WT and IDE-KO mice. Iba1 and GFAP are used as microglial and astroglial markers, respectively. Nuclei are stained with DAPI. Insets show close-ups where morphological changes in IDE-KO microglia can be appreciated. The intensity of labeling in WT inset has been multiplied by a factor of 4, to help visualization. **B** Total hippocampal volume (*t*-test; *p* = 0.29). **C** Astrogliosis, measured as the percentage of area occupied by GFAP labeling (*t*-test; *p* = 0.57). **D** Microgliosis, measured as the percentage of area occupied by Iba1 labeling (*U* Mann–Whitney test; *p* = 0.04). In **B**–**D** each point represents an individual mouse (squares = males, circles = females). Horizontal lines depict the mean ± SEM. *N* = 4–6 mice per genotype and sex. Statistical differences were initially assessed by two-way ANOVA considering the factors genotype and sex. Since no sex-differences were detected (hippocampal volume *p* = 0.383, Iba1^+^ area *p* = 0.573, GFAP^+^ area *p* = 0.405), male and female mice of the same genotype were pooled. *p < 0.05. **E**, **F** Object location test (OLT) and Novel Object Recognition Test (NORT) results. *N* = 10 mice per genotype and sex. Each point represents an individual mouse. Lines depict the median ± interquartile range. Statistical differences in OLT and NORT were assessed by two-way ANOVA, considering the factors genotype and sex, followed by all pairwise multiple comparisons using the Holm–Sidak method. Only biologically relevant differences are shown. **p < 0.01

Since early signs of AD start in the hippocampus and the expression of IDE is known to be particularly high in astrocytes [65], we decided to search for potential changes in glial cells caused by IDE loss. We immunostained the hippocampus of 12-month-old WT and IDE-KO mice for the astrocytic marker GFAP and the microglial marker Iba1 (Fig. 1A). Septal hippocampal volume does not change between WT and IDE-KO mice (Fig. 1B), which suggests that global cell numbers are similar in both genotypes. No differences were appreciated in the astrocytic labeling between genotypes, with no significant changes in the percentage area occupied by the GFAP marker (Fig. 1C). In contrast, we observed a significant increase in Iba1^+^ area fraction in the IDE-KO hippocampi (Fig. 1D) that is accompanied by a remarkable morphology change in KO microglia, which show enlarged soma and more numerous and thicker processes (Fig. 1A, insets). These results reveal specific effects of IDE in the hippocampus, particularly in microglial physiology, leading us to focus our analysis on microglial cells.

### Performance on hippocampal-dependent memory tests is influenced by IDE gene dose

Microglia are known to have important roles in hippocampal-dependent memory processes, where microglial phagocytosis of apoptotic newborn neurons modulates neurogenesis [66]. Thus, before proceeding to functional analysis at the cellular level, we tested our cohort of IDE-KO, IDE-HET, and WT male and female mice for their performance in memory tests. We used the OLT, particularly dependent on hippocampal function, and the NORT, dependent on more distributed memory circuits. These tests were preceded by open field analysis of their locomotor behavior. While no differences between genotypes or sexes are observed in NORT performance (Fig. 1E), OLT exhibited significant differences between genotypes in males, where IDE-KO males obtain higher discrimination indexes than WT males, and IDE-HET animals show intermediate or lower scores (Fig. 1F). A potential difference between sexes is also revealed (*p* = 0.09), an expected finding if microglia, with their well-recognized sex-dependent properties [67], do participate in the final outcomes observed.

Our open field study of locomotor behavior revealed a single genotype-related difference (Additional file 1: Table S1.1). The number of ambulatory episodes was decreased in IDE-KO females in comparison with WT female mice, with IDE-HET females showing intermediate values. This significant, though mild, effect does not relate to female performance in the memory tests. Open field, however, did reveal consistent significant differences between sexes in the total distance travelled, with females moving longer distances than males. No signs of anxiety were present in the animal cohort, with stereotypic counts and center/periphery time ratio unaltered by genotype or sex (Additional file 1: Table S1.1).

### An unambiguous barcode characterizes IDE-deficient 12-month-old mice

An extensive characterization of our 12-month-old WT, IDE-HET and IDE-KO mice cohort is presented in Additional file 1. A multivariate analysis was performed after measuring systemic metabolism-related variables, and nervous system-related variables. For a molecular analysis by immunoblot (Additional file 1: Fig. S1.2), we chose the olfactory bulb, the brain region with the highest expression of insulin receptors in mice [44, 45]. Correlation and PCA reveal coordinated changes of variables in each individual mouse, and relationships among variables in their relative contributions to total variability in the sample. Interesting relationships are revealed (Additional file 1: Fig. S1.3). For example, the two behavioral tests were opposite to each other and orthogonal to the genotype, and IDE protein levels contribute in the same direction as the metabolic parameters in component 1 of PCA, while in component 2 IDE clusters with gliosis markers. Insulin pathway components correlate negatively with weight as expected, and sex differences are also observed. Interestingly, IDE protein dose neither correlates with plasma insulin or glucose levels nor with insulin receptors in the olfactory bulb, also revealed by their orthogonal relationship in the PCA. However, IDE protein levels do correlate negatively with Akt levels, ApoD, gliosis markers, and Aβ.

To further assess if this set of variables is able to significantly differentiate mice by their genotype, we performed a multinomial logistic regression. The model successfully predicts the group classification: 100% of IDE-KO mice were correctly classified, while the success rate for both WT and IDE-HET groups was 80%, with a global misclassification error of 13.3% (Additional file 1: Table S1.2). In conclusion, this multivariate analysis, where the meaning and relationships between variables are blind to the workflow, produced an unambiguous “barcode” for the IDE-knockout mice.

Our data, therefore, disclose IDE relationship with brain-relevant variables and, particularly, that IDE influences hippocampal function in vivo*.* These results clearly point to alterations in microglia, probably resulting from complex functional states altering multiple variables, an unexpected role for IDE deserving further analysis at the cellular level.

### IDE absence in microglia modulates the time course of myelin phagocytosis in a sex-dependent way

For the functional analysis of IDE at the cellular level, we performed IDE-KO and WT microglial primary cultures from male and female neonates (single individual/culture). A first question that arises is whether IDE might influence phagocytic activity in microglial cells. Knowing that IDE is associated with the cytoplasmic side of membranes in microglial cells [22], we hypothesized that IDE-KO microglia might have defects in phagocytosis, a process that involves important membrane rearrangements.

**Fig. 2:**
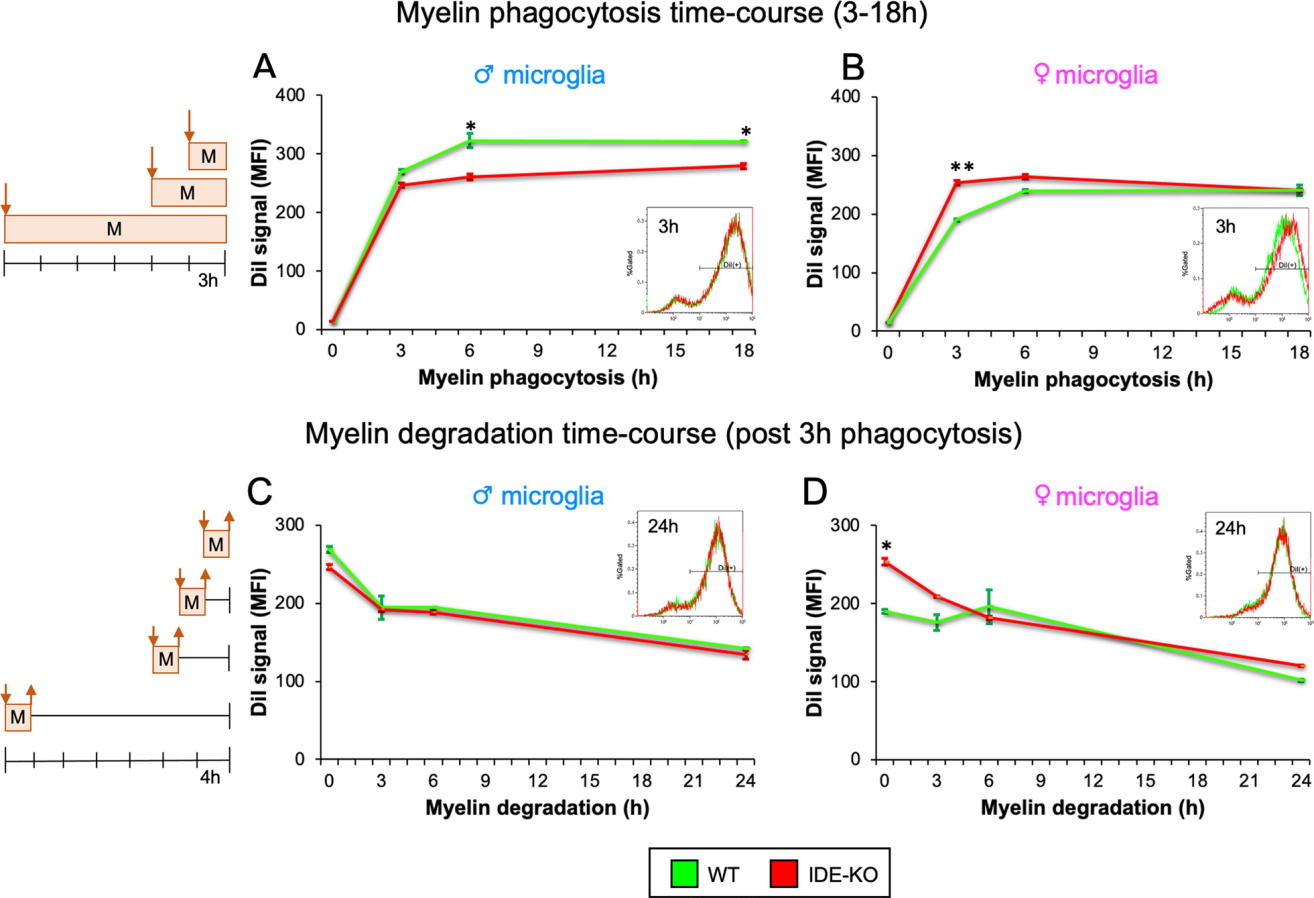
Myelin phagocytosis by microglia shows sex- and IDE-dependent effects, while myelin degradation remains unaltered. Myelin management was assessed by flow cytometry experiments. The histogram of control microglia without myelin was used to define the fluorescence threshold to classify an event as DiI-positive microglia. **A**, **B** Time-course experiments of myelin phagocytosis by WT and IDE-KO male (**A**) and female (**B**) microglia exposed to myelin-DiI for 3–18 h. Histograms of a representative experiment of 3 h phagocytosis are shown in the insets. **C**, **D** Time-course experiments of myelin degradation by WT and IDE-KO male (**C**) and female (**D**) microglia exposed to myelin-DiI for 3 h (phagocytosis time), followed by degradation times ranging from 3 to 24 h. Histograms of a representative experiment of 24 h degradation are shown. Dots in the graphs represent the average ± SEM of 2 independent experiments with 2 male and 2 female cultures, with at least 10,000 cells/sample. Statistical differences were assessed by three-way ANOVA considering the factors genotype, sex and time, followed by post-hoc pairwise comparisons by Holm–Sidak method. Sex factor was significant in both phagocytosis (*p* < 0.001) and degradation (*p* = 0.04) experiments. Phagocytosis in males: factor genotype (*p* = 0.005), WT vs IDE-KO at 3 h (*p* = 0.23), 6 h (*p* = 0.011), 18 h (*p* = 0.049). Phagocytosis in females: factor genotype (*p* = 0.009), WT vs IDE-KO at 3 h (*p* = 0.003), 6 h (*p* = 0.12), 18 h (*p* = 0.98). Degradation in males: factor genotype (*p* = 0.28), WT vs IDE-KO at 0 h (*p* = 0.21), 3 h (*p* = 0.87), 6 h (*p* = 0.72), 18 h (*p* = 0.67). Degradation in females: factor genotype (*p* = 0.07), WT vs IDE-KO at 0 h (*p* = 0.03), 3 h (*p* = 0.22), 6 h (*p* = 0.56), 24 h (*p* = 0.46). *p < 0.05; **p < 0.01

We assessed phagocytic activity by flow cytometry after exposing microglia to DiI-labeled myelin for 3–18 h. We quantified the mean fluorescence intensity (MFI) for each sample and found that lack of IDE has distinct effects in both sexes: male IDE-KO microglia exhibited lower myelin uptake than WT microglia at any time explored (Fig. 2A), suggesting an impaired phagocytosis process, while a transient increase in myelin phagocytosis is observed in IDE-KO female microglia (peak at 3 h, Fig. 2B).

We then evaluated myelin degradation after a 3-h phagocytosis period. IDE genotype does not result in myelin clearance differences in male microglia (Fig. 2C), and female microglia are able to compensate for the excess myelin phagocytosed at the starting point, resulting in similar final levels of myelin degradation (Fig. 2D). Overall, these data indicate that IDE affects in a sex-dependent way the time-course of myelin phagocytosis, with less efficient uptake in male microglia, but without major alteration of the myelin degradation process in both sexes.

### IDE absence in microglia does not significantly affect Aβ oligomers internalization and degradation

**Fig. 3:**
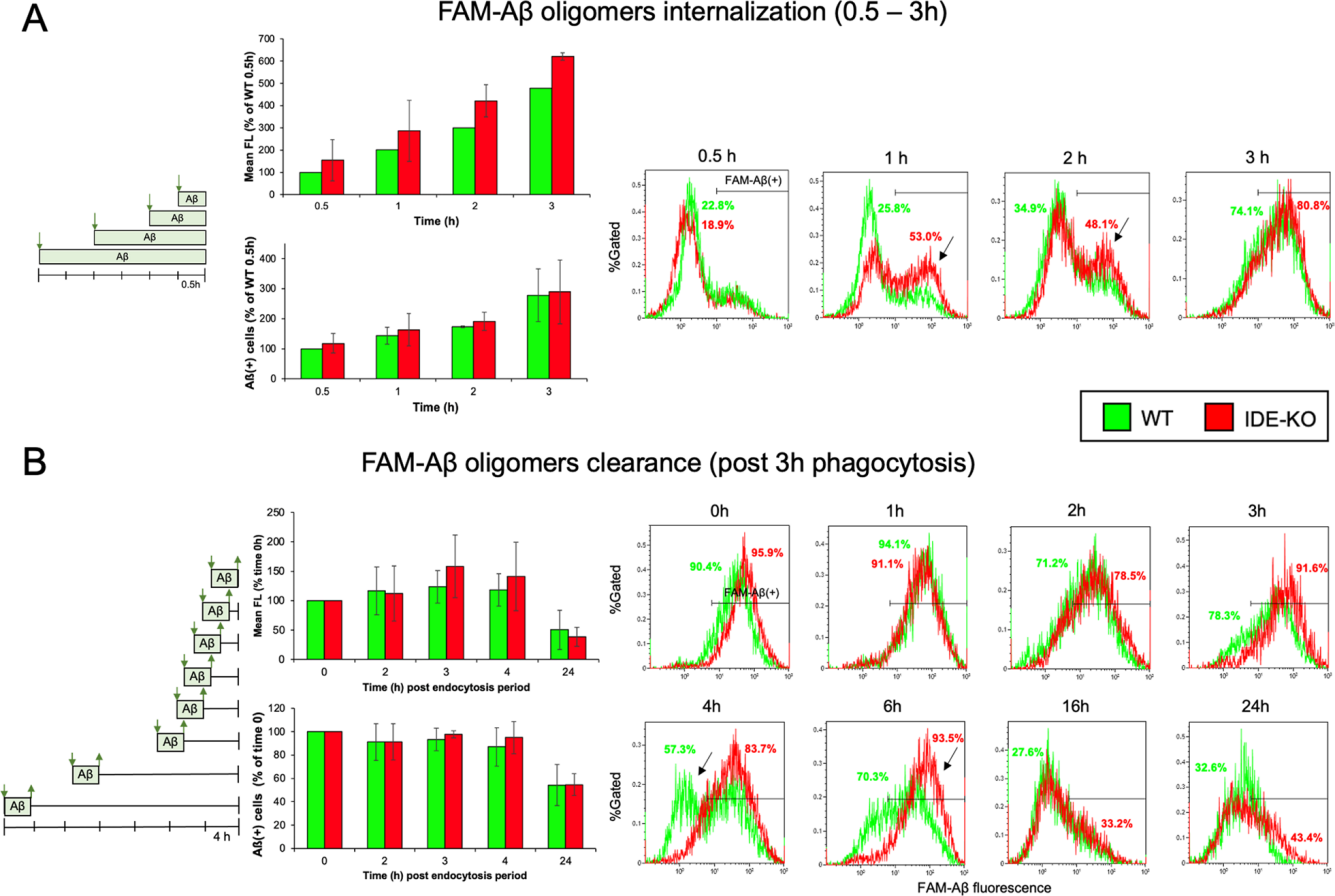
Aβ oligomers internalization and clearance is independent on microglial IDE genotype. **A** FAM-Aβ oligomers internalization measured by flow cytometry after different exposure times (0.5–3 h). Four independent experiments (2–4 samples/time point) were analyzed. Normalized average fluorescence change, and % of Aβ positive cells are represented. Histograms of the experiment with the highest difference between genotypes show a mild, transitory acceleration of endocytosis. The control histogram without FAM-Aβ exposure defines the fluorescence threshold to classify an event as FAM-Aβ positive microglia. Numbers depict the percentage of FAM-Aβ( +) cells. Arrows point out the greatest differences. **B** Time course of Aβ oligomers clearance measured by flow cytometry in cells exposed to FAM-Aβ oligomers for 3 h (internalization period), followed by several degradation times (0–24 h). Three independent experiments with at least 10,000 cells per sample and 2–4 samples/time point were analyzed. Normalized average fluorescence change, and % of Aβ positive cells are represented. Histograms of the experiment with the highest difference between genotypes shows a transitory deceleration of clearance. No statistical differences are found at any time point in both assays. Statistical differences were assessed by 2-way ANOVA considering the factors genotype and time, followed by post-hoc Holm–Sidak comparisons. Endocytosis mean FL [genotype *p = *0.078; time *p = *0.0003]; endocytosis %Aß( +) cells [genotype *p = *0.534; time *p = *0.0252]; clearance mean FL [genotype *p = *0.5636; time *p = *0.0333]; clearance %Aß(+) cells [genotype *p = *0.6752; time *p = *0.0155]

Microglial control of amyloid peptide accumulation in the extracellular milieu is expected to condition the progression of AD [68]. Furthermore, we have found that primary WT microglia respond to the presence of Aβ oligomers by releasing CD81/IDE positive exosomes [22]. It is, therefore, pertinent to test the effect of IDE loss in the processes of endocytosis and degradation of Aβ oligomers. Aβ internalization experiments were performed by exposing WT and IDE-KO microglial cultures to FAM-Aβ oligomers for 0.5–3 h and analyzing fluorescence by flow cytometry. No statistical difference was observed in Aβ internalization (Fig. 3A) and, at most, IDE-KO microglia showed a tendency to transiently accelerate the uptake of FAM-Aβ oligomers in comparison to WT cells. At longer time points Aβ internalization was always equivalent in both genotypes.

Aβ clearance was monitored for 0–24 h after a 3-h exposure to FAM-Aβ oligomers. Again, no significative change in Aβ clearance was observed, with some experiments showing a transitory delay of the degradation process in IDE-KO microglia. When a delay was observed, it was compensated at later times (Fig. 3B). These data suggest that no significant alteration in both Aβ oligomers internalization or Aβ degradation rate takes place in the absence of IDE. They also reveal that IDE-KO microglial cells are able to compensate for observed timing defects in the long run, when they occur.

In summary, effects of IDE loss in myelin or Aβ oligomers intake and degradation are at most transitory, revealing that IDE might be implicated in mechanisms regulating these processes, rather than be a necessary factor for the completion of these tasks. Dysregulations of time courses might indicate, instead, a contribution of IDE to microglial proper reading of external stimuli and acquiring an adequate phenotypic state.

### Transcriptomic profiling of microglia reveals that IDE absence alters microglial responses to various types of environmental signals

**Fig. 4:**
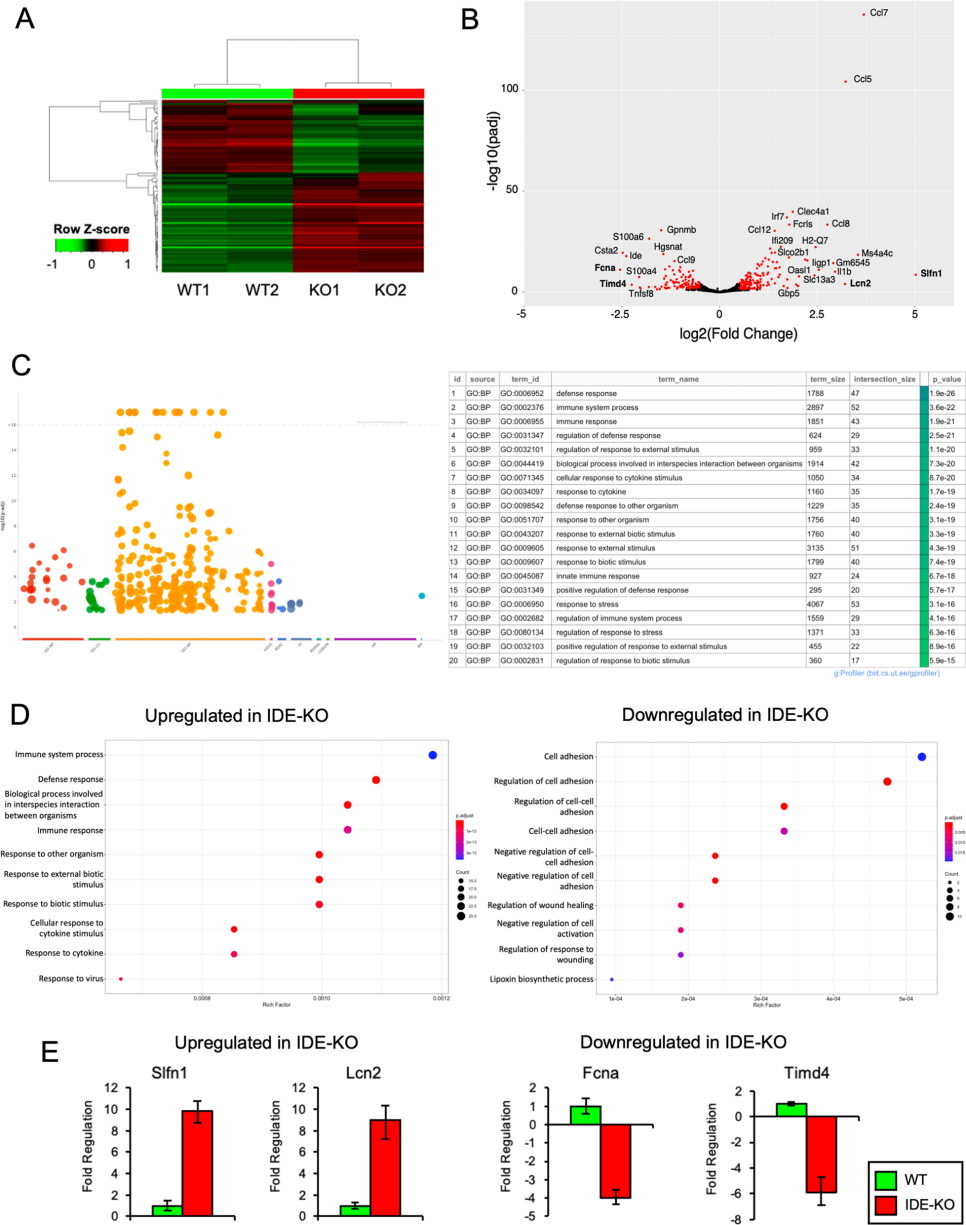
Transcriptomic profiling of WT and IDE-KO primary microglia. **A** Heatmap representation of the top 100 differentially expressed genes (DEGs) in IDE-KO vs. WT microglia (*N* = 2 individual microglial cultures/genotype). Clustering of genes by expression profile is shown on the left. **B** Volcano plot showing in red the genes that are differentially expressed (FDR < 0.05). **C** Gene enrichment analysis using the 103 DEGs. Manhattan plot on the left depicting functional terms grouped by data sources (X-axis) versus the adjusted enrichment p-values in negative log_10_ scale (Y-axis). Circle sizes are in accordance with the corresponding term size. Data sources: *GO* Gene Ontology (with three major categories: *MF* Molecular Functions, *BP* Biological Process, and *CC* Cell Component); Biological pathway databases (KEGG, *REAC* Reactome, and *WP* WikiPathways); regulatory motifs in DNA (*TF* Transcription Factors; *MIRNA* micro-RNAs), Protein databases (CORUM and *HP* Human Protein Atlas). The table on the right has the top 20 most significant GO terms, sorted by p-values. **D** Gene enrichment analyses performed separately in upregulated (left) and downregulated (right) DEGs in IDE-KO vs. WT microglia, showing the top 10 GO terms. The Rich Factor is calculated as the ratio between the number of target genes belonging to a pathway and the number of all annotated genes located in the pathway. The size of the dots indicates the number of target genes in the pathway, while dot's color reflects the different p-value range. **E** Validation of RNA-Seq data by qPCR using RNA from an independent pair of microglial cultures

To globally understand the impact of IDE absence on microglial physiology, we compared the transcriptomic profiles of primary cultures of WT and IDE-KO microglia by bulk RNA-Seq (Fig. 4A). We identified a total of 430 differentially expressed genes (DEGs) in IDE-KO microglia in comparison with WT cells (FDR < 0.05, as shown in Fig. 4B). A complete list of DEGs can be found in Additional file 2. For all subsequent comparisons, we considered as DEGs those with an absolute log_2_ fold change equal or greater than 1 (log_2_FC ≥ 1; twofold change), which resulted in 103 DEGs (59 upregulated and 44 downregulated genes in IDE-KO microglia). Within the set of upregulated genes with an assigned function in "MouseMine" database (MGI), the largest differences were observed for *Slfn1* (a negative regulator of G1/S transition in the mitotic cell cycle) and for the chemokines *Ccl7* (involved in the response to cytokines and in chemotaxis) and *Ccl5* (a negative regulator of apoptotic processes and positive regulator of chemotaxis and response to TNF). Among the downregulated genes, the largest differences were observed for *Fcna* (involved in carbohydrate binding and lectin pathways and acting on complement activation), *Csta2* (with cysteine-type endopeptidase inhibitor activity) and *Timd4* (acting on apoptotic cell clearance by enabling phosphatidylserine binding activity). To delve into the biological meaning of the set of DEGs, a gene set enrichment analysis was performed (Fig. 4C), revealing that the main biological processes affected were related to defensive responses, cytokine signaling, immune system and responses to stimulus, stress and inflammation. More specifically, the set of upregulated genes in IDE-KO microglia is involved in defence and immune responses, including response to stimulus and cytokines (Fig. 4D, left), while downregulated genes are mainly involved in cell adhesion regulation, wound healing, and regulation of cell activation (Fig. 4D, right). A more detailed analysis can be found in Additional file 2.

Validation of RNA-seq results using qRT-PCR (selected genes in bold letters in Fig. 4B) shows a clear overexpression of *Slfn1* and *Lcn2* in IDE-KO microglia, whereas *Fcna* and *Timd4* are significantly downregulated in microglia lacking IDE (Fig. 4E).

Our RNA-Seq results reveal IDE involvement in the microglial response to various types of environmental signals, including those triggering apoptotic cell phagocytosis (required in the neurogenic niches), responses to stress and inflammation signals, and processes that control microglial numbers (cell division or apoptosis) and movement (migration). Therefore, we expanded our experimental tests to the following biological processes: cell-death/survival balance upon stressful stimuli, cell proliferation, and cytokine production profiles upon reception of neuroinflammation and neurodegeneration relevant stimuli.

### Microglial viability is not significantly altered by the absence of IDE in conditions mimicking neuroinflammation, oxidative stress, or amyloid stress

**Fig. 5:**
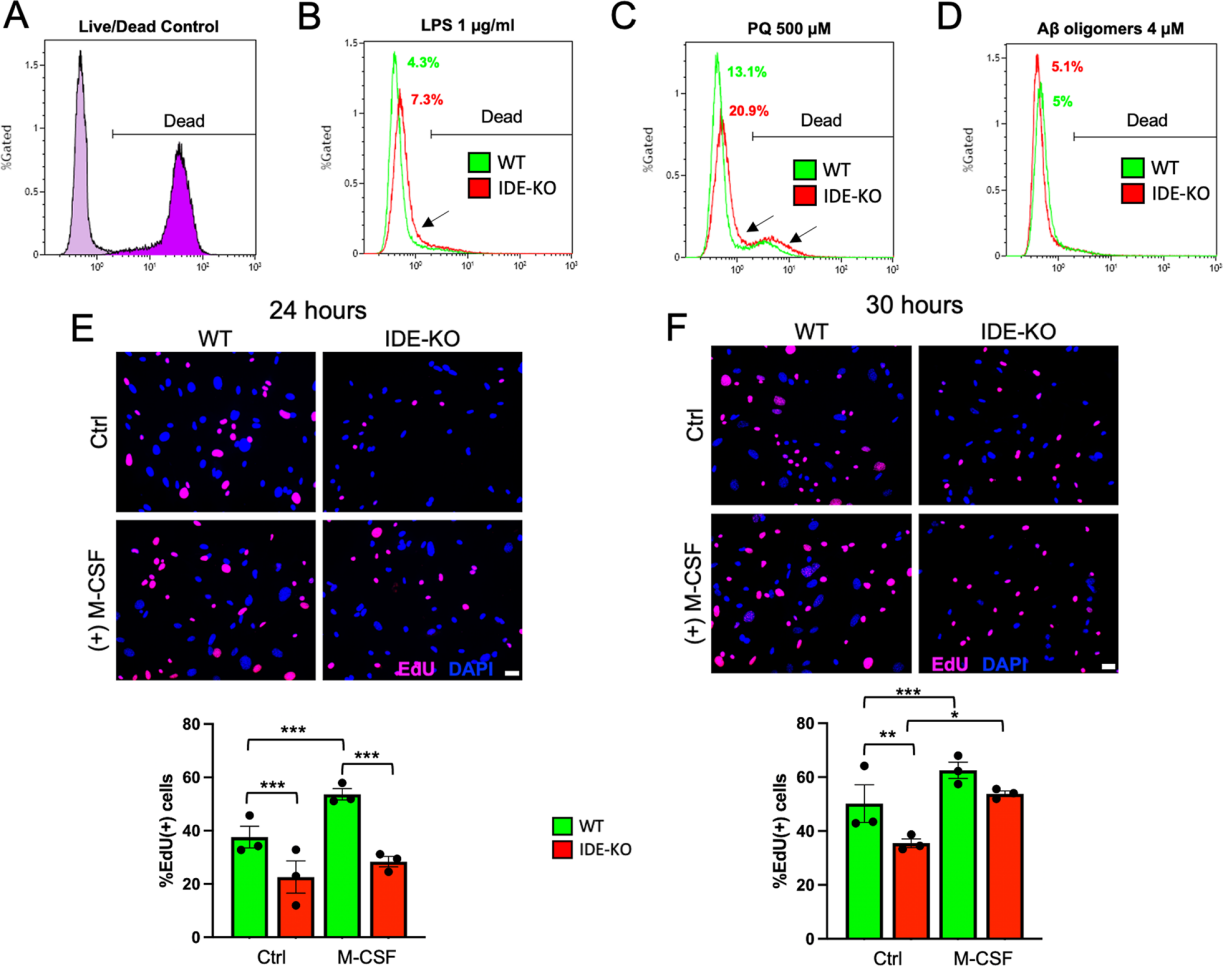
IDE absence decreases microglial proliferation, delays response to M-CSF and produces no significant changes in viability. **A**–**D** Flow cytometry experiments using Live/Dead viability assays to compare WT and IDE-KO microglial viability when stimulated for 8 h with 1 μg/ml LPS (**B**), 500 μM paraquat (**C**) and 4 μM Aβ oligomers (**D**), respectively. The positive control is shown in **A**. Two independent experiments with at least 20,000 cells per sample and 2–4 samples/genotype and stimulus were analyzed (histograms of the experiment with the highest difference between genotypes is shown). Statistical differences in **B**–**D** were assessed by two-way ANOVA, considering the factors genotype (*p*-value = 0.3968) and treatment (*p*-value = 0.0048), followed by all pairwise comparisons between genotypes by Holm–Sidak (LPS *p*-value = 0.6692, PQ *p*-value = 0.5382, Aβ *p*-value = 0.8150). **E**, **F** EdU proliferation assays to quantify WT and IDE-KO primary microglia proliferation under control conditions (Ctrl) and upon M-CSF (macrophage-colony stimulating factor, 50 ng/ml) for 24 h (**E**) and 30 h (**F**). Bars represent the mean ± SEM of EdU-positive cells (3 biological samples, with *N* = 600–1800 cells/condition. Scale bar = 20 μm. Statistical differences in **E** and **F** were evaluated by two-way ANOVA, considering the factors genotype (*p* < 0.001) and treatment (*p* < 0.001), followed by all pairwise multiple comparisons by Holm–Sidak method. Only biologically relevant differences are shown. *p < 0.05; **p < 0.01; ***p < 0.001

We first evaluated if the absence of IDE expression in primary microglia influences their death/survival balance in different conditions. Daily observations of WT and IDE-KO cultures indicated that cell viability was not compromised in IDE-KO microglia under control conditions. We then analyzed cell viability by flow cytometry, using the Live/Dead viability assay (Fig. 5A) after challenging microglia for 8 h with different types of stressors. Neither LPS exposure, mimicking an inflammatory insult, nor severe oxidative stress, induced by PQ exposure, resulted in significant reductions of microglial survival of both genotypes, with only minor differences evidenced in one of the experiments (shown as the “extreme” experiment in Fig. 5B, C). The challenge with Aβ oligomers presented the smallest effect on survival and, again, no difference between genotypes (Fig. 5D). These results highlight microglial endurance when encountering Aβ oligomers, that, as shown above (Fig. 3), they are able to endocytose and degrade without much difficulty. They are also resistant to neuroinflammatory or oxidative stress conditions concomitant to so many neurodegenerative diseases. Collectively, our results indicate no significant effects of IDE function on microglial viability and are coherent with the upregulation of *Ccl5* in IDE-KO microglia. Only the most toxic stimuli tested (pro-inflammatory and oxidative) showed, in some cultures, a slightly higher mortality in IDE-KO microglia.

### Loss of IDE impairs microglial proliferation in response to M-CSF

We then tested if proliferation, a relevant biological process for microglia, as pointed out by our RNA-Seq analysis, might be potentially dependent on IDE. Based on *Slfn1* upregulation in IDE-KO, we hypothesized that the lack of IDE in microglia might result in impaired proliferation. Thus, we analyzed microglial proliferation using the Edu Click-iT assay. Primary microglial cultures were exposed to EdU for 24 and 30 h in complete medium in the presence or absence of M-CSF, a stimulator of microglial proliferation via the CSF1R pathway [69]. At 24 h, IDE-KO microglia proliferated significantly less than WT cells and, while WT microglia showed an increase in their proliferation when incubated with M-CSF, IDE-KO cells were not responsive to this cytokine (Fig. 5E). Interestingly, response to M-CSF is observed at 30 h (Fig. 5F). These results indicate that microglial proliferation is decreased in the absence of IDE, and the putative mechanism for this impairment is a delayed response to M-CSF. This effect might result in the discoordination of microglial responses to natural or pathological stimuli requiring their proliferation.

### IDE modulates the secretion of specific subsets of cytokines in response to inflammation, oxidative stress, and amyloid stimuli

All results described so far; the changes observed in hippocampal microglia, the effects on myelin debris management, their delayed response to mitogens, and the transcriptomic changes; support the hypothesis that IDE plays an active role in the modulation of microglial phenotypic states. So far, our analysis questions that this regulatory role is directly related to “effector functions” as a protease, though it cannot be discarded in still unexplored situations. This new frame of ideas is coherent with previous works where microglia phenotypic switches were correlated with IDE expression [46, 70]. However, attention should be paid, since IDE net expression but also traffic (export into extracellular vesicles) is modified by stimulus-induced switches in microglial phenotypes [22]. Therefore, we characterized the cytokine production profiles of primary male and female microglia subjected to different stimuli, relevant to neuroinflammatory or degenerative processes, and across different microglial polarization axes. Data obtained from cytokine profiling are summarized in Additional file 3.

**Fig. 6:**
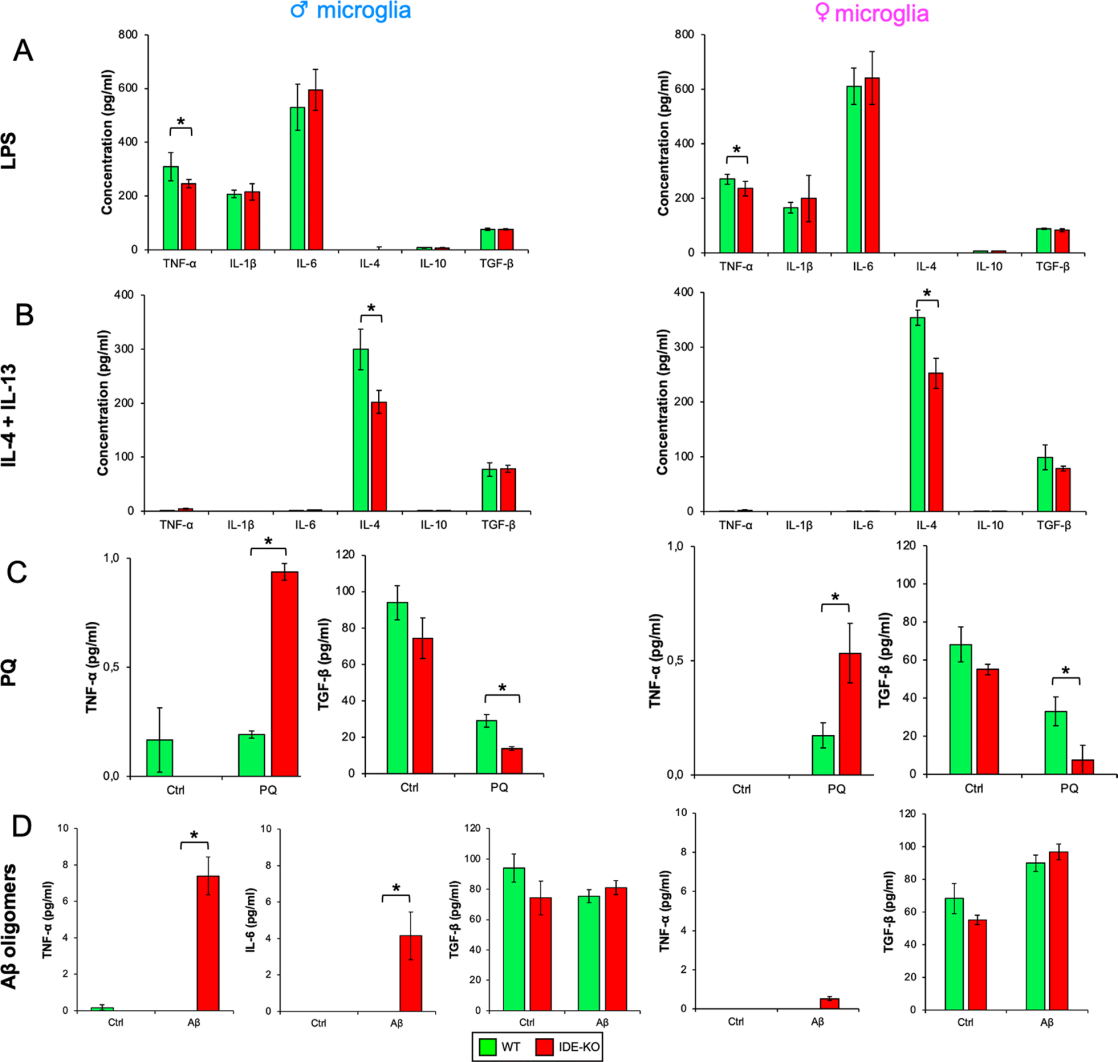
IDE-KO microglia exhibit impaired cytokine profiling when activated with different stimuli. WT and IDE-KO primary microglial cultures were challenged with different stimuli for 18 h, and then cytokines released to the culture medium were measured. Stimuli tested include: **A** LPS (100 ng/ml), **B** IL-4 + IL-13 (20 and 50 ng/ml, respectively), **C** paraquat (50 µM) and **D** Aβ oligomers (1 µM). *N* = 3 samples per genotype and sex. Bars represent the mean ± SEM for each cytokine (unstimulated condition is not plotted in **A** and **B**). Statistical analyses were performed separately in males and females by two-way ANOVAs, considering the factors genotype and treatment. Individual differences were assessed by post hoc Holm–Sidak pairwise comparisons. *p < 0.05

A first conclusion of this profiling (Fig. 6) is that our microglial cultures are in a homeostatic condition, not producing detectable cytokines when unstimulated (with the exception of TGF-β). LPS treatment triggered, as expected, a potent pro-inflammatory response, with high production of IL-1β, TNF-α, and IL-6. However, no significant differences were observed among genotypes in these cytokines except for TNF-α, showing a slight decrease in the absence of IDE in both male and female microglia (Fig. 6A). Conversely, IL-4 + IL-13 stimulation resulted in a high release of IL-4 and, interestingly, IDE-KO microglia secreted a significantly lower amount of this cytokine in both sexes, but no genotype-dependent change is observed in TGF-β (Fig. 6B). Our results demonstrate that IDE is required for the cytokine response organized in both phenotypic states.

PQ-induced oxidative stress promotes TNF-α production and reduces the secretion of TGF-β in WT microglia from both sexes. No other cytokine responds to this stimulus. The lack of IDE resulted in an exacerbated production of TNF-α, accompanied by a marked decrease in TGF-β relative to WT microglia in both sexes, with no changes in the other cytokines tested (Fig. 6C). Thus, PQ-triggered TGF-β decrease and TNF-α increase were more pronounced in IDE-KO microglia, suggesting IDE as an important factor for restraining microglial responses to oxidative stress. Finally, Aβ oligomers treatment triggered the production of TNF-α and IL-6 exclusively in male IDE-KO microglia, indicating a sex- and genotype-specific response to Aβ, while no significant changes were observed for TGF-β production (Fig. 5D). The rest of cytokines tested do not show Aβ-dependent changes by genotype or sex (Additional file 3).

Collectively, these data support that IDE absence results in impaired stimulus-mediated microglial phenotypic state modulation, with both inhibitory or stimulatory effects on the production of specific subsets of cytokines, and important sex-dependent differences in the response to Aβ oligomers.

## Discussion and conclusions

The physiological role of IDE is not as clear as its name would suggest. The evolutionary conservation of IDE [22] and its widespread distribution in different tissues [26] and subcellular compartments [22, 24] suggest a more general function for this protein, and/or the acquisition of diverse roles throughout evolution, not restricted to the degradation of insulin or Aβ peptides.

The initial studies characterizing IDE as an insulin [23] and Aβ [25] degrading enzyme were performed by incubating these substrates with rat tissue extracts in vitro, in which IDE was identified as the main responsible enzyme for such proteolytic activities. Thus, in these original experiments, the enzyme and its substrates were incubated as free molecules in extracts, omitting their physiological context surrounded by membranes. Recently, electron microscopy analyses in microglia have demonstrated that IDE location is mostly cytosolic. It associates to the cytoplasmic side of plasma and multivesicular body membranes from which it gets internalized in exosomes [22]. Therefore, evidence about the physical interaction of IDE with insulin or Aβ in vivo is still needed, since the presence of the enzyme and its substrates in separate subcellular compartments, insulin or Aβ locate either in the extracellular milieu or inside endosomal vesicles, argues against the possibility of an encounter. In addition, the hypothesis that postulated IDE as a candidate link between AD and T2D [42] based on the competitive inhibition of its proteolytic activity has not been demonstrated yet. With this background in mind, and based on recent evidence that proposes IDE as a moonlighting protein involved in several cellular functions beyond its proteolytic activity [71], we devoted this work to characterize IDE functions in the brain, without assuming that the consequences of IDE functions depend on its proteolytic activity.

Based on previous studies that described that Aβ_40_ and Aβ_42_ [40, 72] are elevated in the brains of IDE deficient mice, and provided that Aβ peptides accumulate preferentially in the hippocampus, we initially performed histological studies in this brain region. Our results revealed a specific effect of IDE in microglia, with IDE-KO microglia showing increased Iba1 expression when compared to WT microglia. In contrast, neither hippocampal volume nor GFAP expression were affected by IDE absence. These results led us to analyze the effect of IDE absence on the brain function by using two memory tests. The OLT, especially dependent on the hippocampus [48], revealed significant differences between genotypes, with IDE-KO males obtaining higher discrimination indexes than WT mice. These results suggest a sex-dependent exaggerated response to the moved object when IDE is absent, and thus, a modulation by IDE of spatial cognition. Conversely, no effects for IDE absence were detected in the NORT, which relies on more distributed circuits [48] underlying memory mechanisms with widespread cortical representations. Interestingly, an inverse correlation between the performance on both tests was described in our multivariate analysis (Additional file 1).

The specific effects of IDE absence in microglia led us to focus our study on this cell type. A previous study shows that *Nlrp3* gene deficiency in a mouse model of AD (APP/PS1 mice) increases IDE (both mRNA and protein) with a concomitant modulation of microglial phenotype—increased expression of *Arg-1*, *Fizz1* and *Il4,* and enhanced Aβ phagocytosis [73]. We, therefore, hypothesized that IDE absence might trigger an impairment in microglial phagocytosis. Within WT microglia, our experiments demonstrate sex-dependent differences in myelin phagocytosis: male WT microglia phagocytosed more myelin than female cells, contrary to recent evidence that suggests that female microglia have a higher phagocytic ability [74]. Factors differing between in vitro and in vivo experiments, including estrogens deprivation in cultured female microglia, might cause this disagreement. Interestingly, we found that IDE absence affects myelin phagocytosis in a sex-dependent manner, decreasing myelin uptake in males and increasing it in females, without affecting myelin degradation, which suggests again that IDE absence has transitory effects on modulating microglia responses.

Microglial cells play a prominent role in the degradation of extracellular Aβ by secretion of proteases, but they also phagocytose Aβ for lysosome-mediated degradation. For the first scenario, extracellular Aβ degradation by secreted IDE has already been described [75, 76]. However, the interaction of Aβ with IDE in vivo still requires demonstration because, attending to their subcellular localization, they would be in compartments physically separated by membranes [22] and would require exosomal membrane rupture to interact. This reason, together with the fact that IDE stably associates with the plasma membranes in microglia and that the internalization of molecules is dependent on membrane-specific changes, led us to focus on the internalization of Aβ oligomers and their intracellular degradation, and on the potential consequences that IDE absence might have on these processes. Contrary to the results reported by Fu and colleagues [77], we did find microglial uptake of Aβ oligomers. The main cause for this disagreement could be the different Aβ oligomer concentrations used: the nanomolar range used by Fu and colleagues [77] might be below detection levels. Remarkably, IDE absence in microglia resulted in subtle alteration with only a transitory acceleration of Aβ oligomers internalization observed in some experiments, a process that will take place at the same time that the release of IL-6 and TNF-α specifically by IDE-KO male cells. Although phagocytosis is enhanced in *Arg-1*/*Fizz1*/*Il4* expressing microglial phenotypes, that concur with IDE increased expression in the APP/PS1mice [73], Aβ internalization can occur by other mechanisms besides phagocytosis, such as endocytosis and pinocytosis [78]. These processes might be differently conditioned by both the activation state of microglia and the absence of IDE in microglial membranes [22]. On the other hand, it is clear that IDE is not required for Aβ oligomers degradation, but it can condition its normal rate. In any case, the putative modulation by IDE of the dynamics of Aβ oligomers clearance cannot be attributed to a direct effect of IDE enzymatic activity on Aβ clearance, since, as mentioned above, they are in different cell compartments unable to physically interact [22]. There are other intracellular mechanisms worth further study, such as intracellular proteases, the ubiquitin–proteasome system or the autophagy pathway [78], that might be altered when IDE is absent in microglia.

The proliferation of microglial cells, regulated by activation of the CSF1R pathway, is a hallmark of many neurodegenerative conditions [79]. Moreover, it has been recently demonstrated that the turnover of microglial populations is a highly dynamic process that happens several times during a healthy lifetime [80]. These are two examples in which microglial proliferation is a crucial physiological process. We observed that IDE-KO microglia proliferate less than WT cells and also have a delayed response to M-CSF. M-CSF is a growth factor constitutively expressed by astrocytes and, to a lesser extent, by microglia, that selectively promotes microglial division through the CSF1R pathway [81]. Upregulation of *Slfn1,* a break to cell division, further highlights the requirement of IDE for appropriate microglial proliferation. Our results align with data obtained by IDE knockdown in the HepG2 cell line (human hepatoma), producing a decrease in cell proliferation attributed in this case to the dysregulation of the p53 pathway [82], and also with a previous work in which IDE downregulation impaired SH-SY5Y cell line (human neuroblastoma) proliferation [83].

Cytokine profiling experiments suggest that microglial phenotypes in response to different stimuli are impaired in the absence of IDE, with IL-4 + IL-13 treatment triggering the production of less IL-4 in IDE-KO microglia and, conversely, PQ treatment producing both an increase in TNF-α and a decrease in TGF-β protein in both male and female microglia. These results tally with the work of Heneka and coworkers [70] performed in *Nlrp3* deficient mice, where microglia overexpression of *Arg-1*, *Fizz-1,* and *Il4* was associated with an increase in IDE protein expression. To the knowledge that IDE expression is modulated by or correlates with different phenotypic states of microglia [46, 70, 84–87] the present work adds that, in fact, IDE plays an important role as a modulator of microglial phenotypic states and reveals a previously unknown function for IDE in microglia responses to IL-4 + IL-13, as well as to oxidative and amyloid stress, without altering microglia viability. Our data suggest that IDE has a significant role in dampening the responses of microglia to oxidative stress, a condition that often initiates reverberating positive feedback loops between microglia and astrocytes in pathological conditions [8].In addition, a most striking result is the sex- and genotype-dependent response to Aβ oligomers, with only male IDE-KO microglial cells responding with the secretion of both TNF-α and IL-6. Sex differences in the neuroimmune system, including glial activation and associated cytokine production in the brain, is a recently emerging field [88].

Cytokine responses perfectly align with our global transcriptomic profiling of IDE-KO primary microglia, which strongly supports that IDE is involved in the regulation of immune responses, including the production and response to cytokines, as well as the regulation of the response to several types of stimuli (external, biotic, stress, or interaction with other organisms). In this regard, it is highly relevant the pattern of delayed responses we observed in several microglial functions when IDE is absent – including phagocytosis of myelin, degradation of Aβ oligomers, and response to the mitogen M-CSF –, which suggests that IDE is required by microglia for a proper reading of stimuli and acquisition of an adequate phenotype. Without IDE, the timing of microglial responses to stimuli is impaired, probably due to a blockage at some stage of the mechanisms that regulate cell homeostasis, provoking transitory effects that alter microglial phenotypes. Collectively, our work has unveiled previously unknown non-enzymatic functions for IDE as a modulator of microglial phenotypes, which makes IDE, and the modulation of its expression and traffic [22], a potential therapeutic target for neurodegenerative processes.

### Supplementary Information

**Additional file 1: File S1.** An unambiguous barcode characterizes IDE-deficient 12-month-old mice.

**Additional file 2: File S2.** Transcriptomic profiling of IDE-KO vs. WT primary microglia.

**Additional file 3: File S3.** Cytokine profiling results of IDE-KO vs. WT primary microglia.

## Data Availability

The datasets supporting the conclusions of this article are included within the article (and its additional files).

## Abbreviations

AD: Alzheimer’s disease
Aβ: Amyloid-beta
CNS: Central nervous system
D.I.: Discrimination index
DEGs: Differentially Expressed Genes
DM: Diabetes mellitus
i.p.GTT: Intraperitoneal glucose tolerance test
IDE: Insulin-degrading enzyme
Km: Michaelis constant
KO: Knockout
LPS: Lipopolysaccharide
M-CSF: Macrophage colony-stimulating factor
MFI: Mean Fluorescence Intensity
NORT: Novel object recognition test
OF: Open field
OLT: Object location test
PCA: Principal component analysis
RNA-Seq: RNA sequencing
RT: Room temperature
T1D: Type 1 diabetes
T2D: Type 2 diabetes
WT: Wildtype
