# Supplementary material for: Insulin-degrading enzyme (IDE) as a modulator of microglial phenotypes in the context of Alzheimer’s disease and brain aging
Source: J Neuroinflammation. 2023 Oct 11;20:233. doi: 10.1186/s12974-023-02914-7 (PMC10566021; doi:10.1186/s12974-023-02914-7)
Supplement: Supplementary file 1 — Additional file 1: File S1. An unambiguous barcode characterizes IDE-deficient 12-month-old mice. [file 12974_2023_2914_MOESM1_ESM.docx]

**Insulin-degrading enzyme (IDE) as a modulator of microglial phenotypes in the context of Alzheimer’s disease and brain aging.**

**Miriam Corraliza-Gomez, Teresa Bermejo, Jingtao Lilue, Noelia Rodriguez-Iglesias, Jorge Valero, Irene Cozar-Castellano, Eduardo Arranz, Diego Sanchez and Maria Dolores Ganfornina**

**Supplementary File 1.** **An unambiguous barcode characterizes IDE-deficient 12-month-old mice.**

A multivariate analysis was performed after measuring systemic metabolism-related variables, and nervous system-related variables in our 12-month-old WT, IDE-HET and IDE-KO mice cohort. **Table S1.1** contains values obtained in all variables and results on statistical tests performed. A total of 10 mice per genotype and sex were used for this analysis.

We first checked if total or partial loss of IDE altered body weight: this parameter remained unchanged in female mice, while IDE genotype had a dose-dependent effect in males, with p-values close to α (set to 0.05). Next, we measured blood glucose and plasma insulin levels under 6 hour-fasting conditions within their diurnal period (avoiding hypoglycemia-triggered brain shock). Glucose levels showed a tendency to be decreased in HET mice in both sexes while, intriguingly, KO mice exhibited a bimodal distribution, which could indicate two stages of metabolic impairment. Plasma insulin levels remained unchanged, with no significant differences neither between genotypes nor sexes (**Table S1.1**). To elucidate whether KO mice lost their metabolic phenotype [1–3] due to aging, we performed an i.p.GTT on a small cohort of 6-month-old WT and KO mice (**Figure S1.1A**). KO mice showed a tendency to have glucose intolerance, as reflected by its higher area under the curve (**Figure S1.1B**, p = 0.13), which indicates that *Ide* null mice had diabetic traits early in their life.


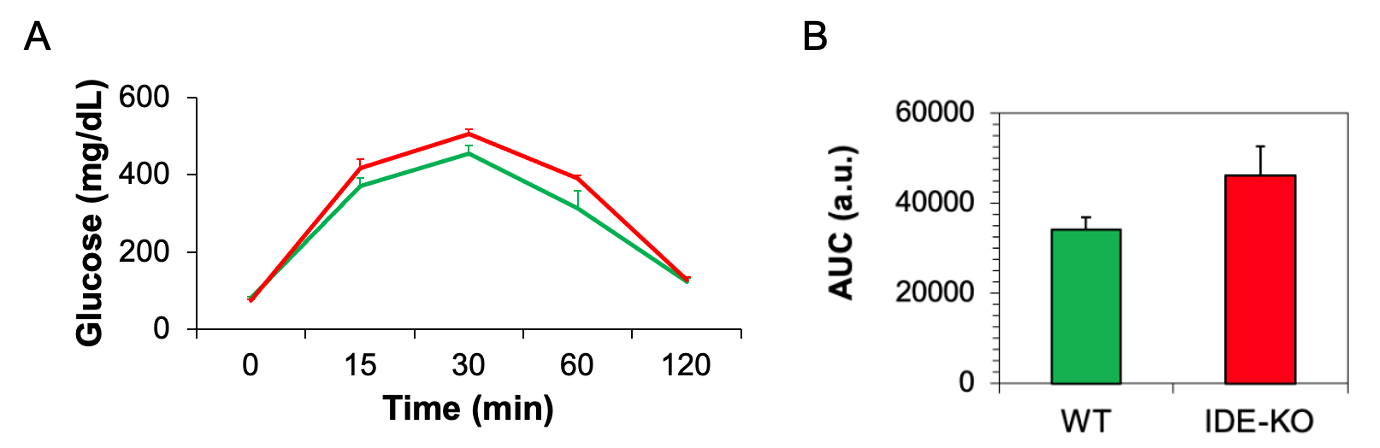


**Figure S1.1. Intraperitoneal glucose tolerance test (ipGTT) at 6 months of age.** **A)** Glycemia is measured after a bolus of Glucose (2 g glucose/kg body weight). **B)** Area under the curve (AUC) for each genotype. Statistical differences were evaluated by t-test (p-value = 0.13). N = 6 WT (3 ♂ and 3 ♀) and 7 IDE-KO mice (4 ♂ and 3 ♀). Since no differences between sexes were detected, male and female mice were pooled for this analysis.

We then performed molecular biology studies in the same cohort by quantitative immunoblot of relevant proteins (examples shown in **Figure S1.2**; complete results summarized in **Table S1.1**). For this aim, we decided to focus our study on the olfactory bulb, since it shows the highest concentration of insulin and insulin receptors in the mouse [4,5]. We analyzed: insulin receptor and AKT1 (downstream of brain insulin signalling), IDE (to analyze its expression levels among genotypes and sexes), CD11b and Iba1 (as microglia phenotype markers), GFAP (astrogliosis marker), ApoD (one of the most consistently upregulated proteins in the mammalian aging brain), and Aβ species (whose accumulation is related with impaired brain function). Again, sex but not genotype resulted in differential protein expression. In some variables, a high dispersion in the sample was observed that could reflect an underlying bimodal population. This opens the possibility to the existence of coordinated changes of variables in each individual mouse.


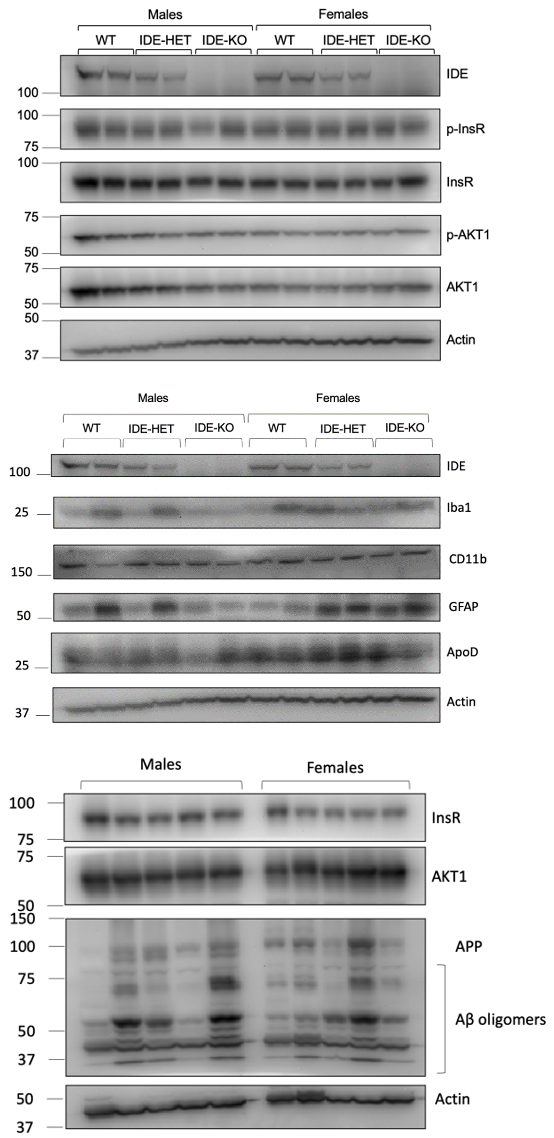


**Figure S1.2. Representative immunoblots of markers included in our multivariate analysis.** A total of 10 mice per genotype and sex were analyzed. Quantitative data shown in Table S1.1.

A correlation analysis was therefore performed to reveal relevant relationships between variables (**Figure S1.3A**). The multivariate analysis detects mild positive correlations between genotype (number of functional copies of *Ide* gene). In addition, OLT index was inversely correlated with CD11b and GFAP (p=0.15 and p=0.16, respectively), while NORT showed weak positive correlations between test performance and gliosis markers (GFAP p=0.1; CD11b p=0.2; Iba1 p=0.2). All metabolic parameters analyzed showed negative correlations with OF parameters, indicating an important effect of metabolism on locomotor behavior. Weight showed a positive correlation with blood glucose, while a negative correlation was found between blood glucose and gliosis markers (CD11b p=0.03; GFAP p=0.09). The correlation analysis allowed us to detect variables with high collinearity (for example distance travelled and ambulatory episodes). Redundant information was discarded in the next steps.

Principal component analysis (PCA) was performed therefore with 18 variables (out of the initial 25 variables) (**Figure S1.3B**). PCA further reduced these variables to 2 new dimensions which explain 41.5% of total variance. These dimensions clearly separated and clustered OF variables (lower left quadrant, yellow), metabolism-related parameters (right quadrants, orange) and gliosis markers (upper quadrants, green). Remarkably, the two behavioral tests were opposite to each other and orthogonal to the genotype. Regarding IDE (protein levels, pink), it contributes to Dimension 1 in the same direction as the metabolic parameters, while in component 2 IDE clusters with gliosis markers.

We further assessed whether the variables contributing to PCA components were able to significantly differentiate mice by their genotype. For that purpose, a multinomial logistic regression was used. The goodness of fit was checked by the Hosmer-Lemeshow test (X^2^ = 3.7, degrees of freedom = 14, p-value = 0.997). **Table S1.2** contains the parameter estimates for both IDE-HET and IDE-KO equations. Overall, the model was predictive of the group classification: the model correctly classified 100% of IDE-KO mice, while the success rate for both WT and IDE-HET groups was 80%, with a global misclassification error of 13.3%.

In summary, individual analyses of behavioral, metabolic, or molecular parameters revealed that the main differences between mice were not genotype-dependent, but sex-related instead. Interestingly, the multivariate analysis, where the meaning and relationships between variables are blind to the workflow, produced an unambiguous “barcode” for the IDE-knockout mice, able to correctly classify the individuals in the sample, and disclosing IDE relationship with brain-relevant variables.


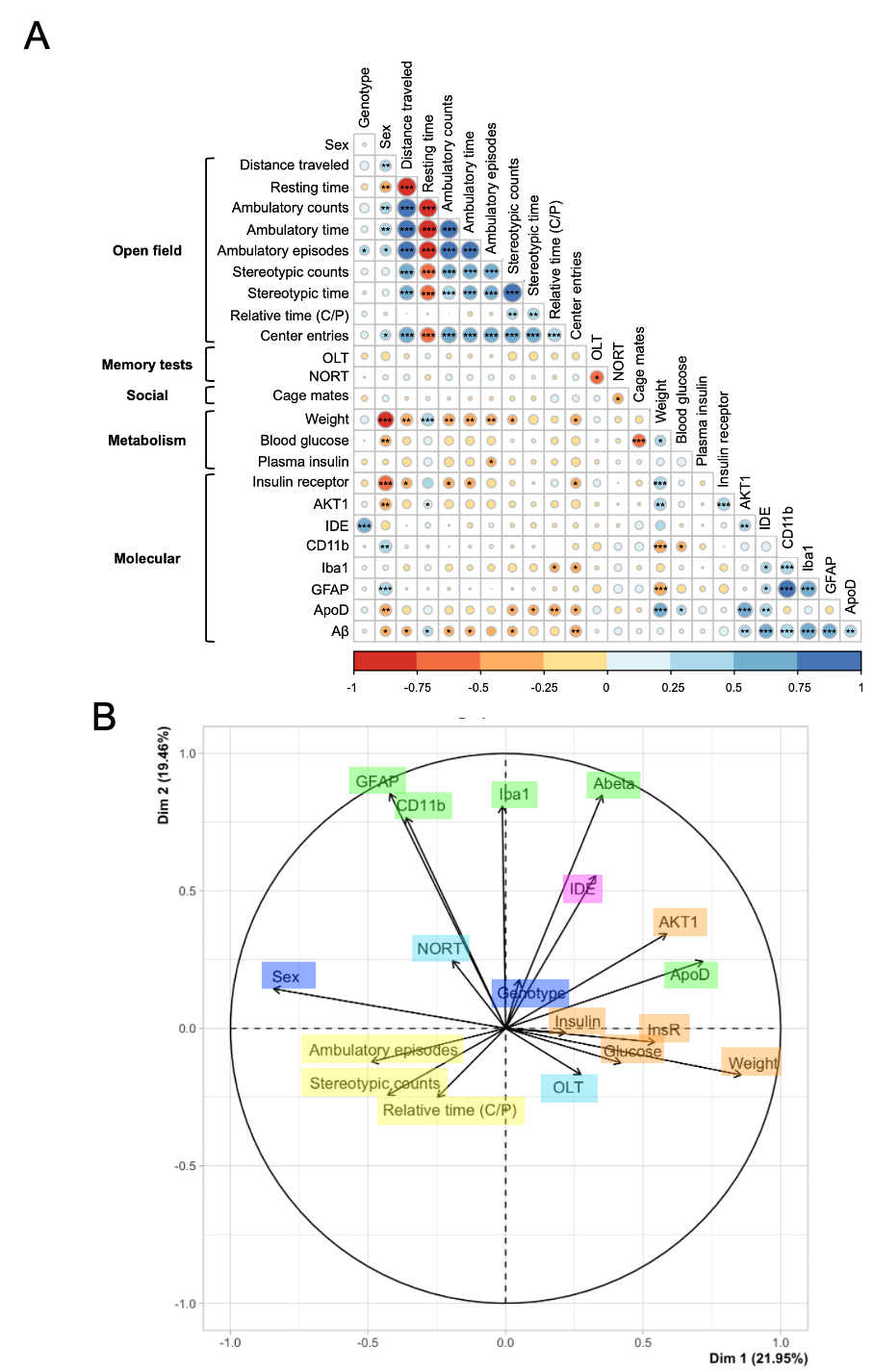


**Figure S1.3. Multivariate analysis A)** Correlation analysis showing all the variables analysed in the cohort. Since the input variables had different scales, data were first standardized and then correlation coefficients were calculated between each pair of variables using the Pearson correlation method. Categorical variables are coded as follows: genotype represents the functional copies of the Ide gene, being WT = 2, IDE-HET = 1, and IDE-KO = 0; while sex is coded as 0 = male, 1 = female. Correlation p-values are shown as follows: *, p<0.05; **, p<0.01; ***, p<0.001. **B)** Principal Component Analysis (PCA) of representative variables (redundant variables or variables with high collinearity were removed).

**References**

1. Farris W, Mansourian S, Chang Y, Lindsley L, Eckman EA, Frosch MP, et al. Insulin-degrading enzyme regulates the levels of insulin, amyloid -protein, and the -amyloid precursor protein intracellular domain in vivo. Proceedings of the National Academy of Sciences. 2003;100:4162–7.

2. Abdul-Hay SO, Kang D, McBride M, Li L, Zhao J, Leissring MA. Deletion of Insulin-Degrading Enzyme Elicits Antipodal, Age-Dependent Effects on Glucose and Insulin Tolerance. Fadini GP, editor. PLoS ONE. 2011;6:e20818.

3. Steneberg P, Bernardo L, Edfalk S, Lundberg L, Backlund F, Östenson C-G, et al. The Type 2 Diabetes–Associated Gene Ide Is Required for Insulin Secretion and Suppression of a-Synuclein Levels in β-Cells. Diabetes. 2013;62:2004–14.

4. Banks WA, Owen JB, Erickson MA. Insulin in the brain: There and back again. Pharmacology & Therapeutics. 2012;136:82–93.

5. Gray SM, Barrett EJ. Insulin transport into the brain. American Journal of Physiology-Cell Physiology. 2018;315:C125–36.

**Table S1.1. Statistical analyses on all the variables analyzed.** *N = 10 mice per genotype and sex. Red asterisks highlight statistically significant results.*

|  | **Variable** | **Statistic** | **WT**♂ | **IDE-HET** ♂ | **IDE-KO** ♂ | **WT** ♀ | **IDE-HET** ♀ | **IDE-KO** ♀ | **2-way ANOVA** | | |
| --- | --- | --- | --- | --- | --- | --- | --- | --- | --- | --- | --- |
|  | **Distance travelled**  **(cm)** | Mean | 1213.14 | 1155.18 | 1117.72 | 1481.44 | 1436.47 | 1242.10 | **Genotype p-value** | **Sex**  **p-value** | **Interaction**  **p-value** |
|  |  | 95% C.I. Mean  Lower Bound  Upper Bound | 997.13 | 935.61 | 928.35 | 1287.78 | 1206.22 | 993.41 | 0.212 | 0.006* | 0.665 |
|  |  |  | 1429.14 | 1374.75 | 1307.08 | 1675.11 | 1666.71 | 1490.80 | **Post-hoc (Holm-Sidak) p-values** | | |
|  |  | Median | 1218.85 | 1034.94 | 1203.47 | 1423.81 | 1376.68 | 1234.70 | **Genotype** | **within ♂** | **within ♀** |
|  |  | Std. Deviation | 301.95 | 306.94 | 264.71 | 270.72 | 321.87 | 347.65 | WT vs KO | 0.485 | 0.084 |
|  |  | Minimum | 768.40 | 772.06 | 626.93 | 1179.35 | 1097.98 | 616.72 | HET vs KO | 0.784 | 0.158 |
|  |  | Maximum | 1888.17 | 1602.35 | 1430.45 | 1928.38 | 2172.00 | 1766.81 | WT vs HET | 0.671 | 0.742 |
|  |  | Range | 1119.77 | 830.29 | 803.52 | 749.03 | 1074.02 | 1150.09 |  |  |  |
|  |  | Interquartile Range | 298.71 | 645.27 | 445.05 | 508.78 | 428.74 | 425.73 |  |  |  |
|  | **Resting time (s)** | Mean | 226.58 | 230.31 | 228.10 | 211.58 | 211.32 | 221.05 | **Genotype p-value** | **Sex**  **p-value** | **Interaction**  **p-value** |
|  |  | 95% C.I. Mean  Lower Bound  Upper Bound | 216.38 | 217.56 | 215.87 | 197.80 | 197.90 | 204.05 | 0.639 | 0.006* | 0.594 |
|  |  |  | 236.77 | 243.05 | 240.33 | 225.35 | 224.75 | 238.04 | **Post-hoc (Holm-Sidak) p-values** | | |
|  |  | Median | 226.70 | 235.40 | 222.63 | 210.50 | 210.02 | 220.84 | **Genotype** | **within ♂** | **within ♀** |
|  |  | Std. Deviation | 14.25 | 17.82 | 17.10 | 19.26 | 18.76 | 23.75 | WT vs KO | 0.856 | 0.263 |
|  |  | Minimum | 199.87 | 201.83 | 210.83 | 174.07 | 173.33 | 185.10 | HET vs KO | 0.793 | 0.250 |
|  |  | Maximum | 250.10 | 252.70 | 260.33 | 236.67 | 236.83 | 262.67 | WT vs HET | 0.658 | 0.976 |
|  |  | Range | 50.23 | 50.87 | 49.50 | 62.60 | 63.50 | 77.57 |  |  |  |
|  |  | Interquartile Range | 17.10 | 34.30 | 28.40 | 30.73 | 24.10 | 22.76 |  |  |  |
|  | **Variable** | **Statistic** | **WT**♂ | **IDE-HET** ♂ | **IDE-KO** ♂ | **WT** ♀ | **IDE-HET** ♀ | **IDE-KO** ♀ | **2-way ANOVA** | | |
|  | **Ambulatory counts** | Mean | 571.70 | 553.30 | 487.00 | 746.40 | 738.10 | 575.40 | **Genotype p-value** | **Sex**  **p-value** | **Interaction**  **p-value** |
|  |  | 95% C.I. Mean  Lower Bound  Upper Bound | 429.72 | 406.46 | 385.17 | 618.98 | 584.31 | 414.98 | 0.085 | 0.005* | 0.695 |
|  |  |  | 713.68 | 700.14 | 588.83 | 873.82 | 891.89 | 735.82 | **Post-hoc (Holm-Sidak) p-values** | | |
|  |  | Median | 561.00 | 463.00 | 533.00 | 697.00 | 683.50 | 565.50 | **Genotype** | **within ♂** | **within ♀** |
|  |  | Std. Deviation | 198.47 | 205.27 | 142.34 | 178.12 | 214.99 | 224.26 | WT vs KO | 0.338 | 0.056 |
|  |  | Minimum | 331.00 | 307.00 | 234.00 | 530.00 | 483.00 | 201.00 | HET vs KO | 0.452 | 0.069 |
|  |  | Maximum | 1057.00 | 858.00 | 646.00 | 1030.00 | 1245.00 | 937.00 | WT vs HET | 0.834 | 0.925 |
|  |  | Range | 726.00 | 551.00 | 412.00 | 500.00 | 762.00 | 736.00 |  |  |  |
|  |  | Interquartile Range | 180.25 | 412.50 | 251.00 | 344.50 | 254.50 | 251.00 |  |  |  |
|  | **Ambulatory time (s)** | Mean | 52.34 | 49.55 | 50.84 | 66.70 | 67.87 | 57.43 | **Genotype p-value** | **Sex**  **p-value** | **Interaction**  **p-value** |
|  |  | 95% C.I. Mean  Lower Bound  Upper Bound | 42.77 | 37.69 | 40.84 | 53.98 | 54.81 | 42.02 | 0.567 | 0.005* | 0.550 |
|  |  |  | 61.91 | 61.41 | 60.84 | 79.42 | 80.93 | 72.84 | **Post-hoc (Holm-Sidak) p-values** | | |
|  |  | Median | 52.20 | 44.36 | 54.93 | 67.09 | 68.95 | 57.72 | **Genotype** | **within ♂** | **within ♀** |
|  |  | Std. Deviation | 13.38 | 16.58 | 13.99 | 17.78 | 18.26 | 21.54 | WT vs KO | 0.846 | 0.232 |
|  |  | Minimum | 32.50 | 29.83 | 25.37 | 46.47 | 44.00 | 22.30 | HET vs KO | 0.867 | 0.179 |
|  |  | Maximum | 77.96 | 75.76 | 67.57 | 103.97 | 103.97 | 91.43 | WT vs HET | 0.718 | 0.880 |
|  |  | Range | 45.46 | 45.93 | 42.20 | 57.50 | 59.97 | 69.13 |  |  |  |
|  |  | Interquartile Range | 19.14 | 33.07 | 24.45 | 26.29 | 24.85 | 22.01 |  |  |  |

|  | **Variable** | **Statistic** | **WT**♂ | **IDE-HET** ♂ | **IDE-KO** ♂ | **WT** ♀ | **IDE-HET** ♀ | **IDE-KO** ♀ | **2-way ANOVA** | | |
| --- | --- | --- | --- | --- | --- | --- | --- | --- | --- | --- | --- |
|  | **Ambulatory Episodes** | Mean | 62.60 | 63.30 | 59.00 | 80.70 | 74.60 | 62.50 | **Genotype p-value** | **Sex**  **p-value** | **Interaction**  **p-value** |
|  |  | 95% C.I. Mean  Lower Bound  Upper Bound | 51.03 | 51.88 | 47.79 | 71.72 | 66.46 | 46.88 | 0.089 | 0.010* | 0.357 |
|  |  |  | 74.17 | 74.72 | 70.21 | 89.68 | 82.74 | 78.12 | **Post-hoc (Holm-Sidak) p-values** | | |
|  |  | Median | 63.00 | 61.00 | 64.00 | 78.00 | 73.50 | 64.00 | **Genotype** | **within ♂** | **within ♀** |
|  |  | Std. Deviation | 16.17 | 15.96 | 15.68 | 12.55 | 11.37 | 21.83 | WT vs KO | 0.616 | 0.014* |
|  |  | Minimum | 38.00 | 42.00 | 27.00 | 63.00 | 57.00 | 27.00 | HET vs KO | 0.549 | 0.095 |
|  |  | Maximum | 94.00 | 88.00 | 77.00 | 100.00 | 90.00 | 93.00 | WT vs HET | 0.922 | 0.396 |
|  |  | Range | 56.00 | 46.00 | 50.00 | 37.00 | 33.00 | 66.00 |  |  |  |
|  |  | Interquartile Range | 20.00 | 31.00 | 24.00 | 25.00 | 23.00 | 31.00 |  |  |  |
|  | **Stereotypic Counts** | Mean | 443.70 | 424.8 | 429.50 | 466.10 | 436.00 | 448.70 | **Genotype p-value** | **Sex**  **p-value** | **Interaction**  **p-value** |
|  |  | 95% C.I. Mean  Lower Bound  Upper Bound | 410.06 | 394.89 | 370.79 | 429.72 | 420.71 | 403.04 | 0.361 | 0.216 | 0.946 |
|  |  |  | 477.34 | 454.71 | 488.21 | 502.48 | 451.29 | 494.36 | **Post-hoc (Holm-Sidak) p-values** | | |
|  |  | Median | 451.50 | 433.00 | 433.00 | 472.00 | 430.00 | 464.50 | **Genotype** | **within ♂** | **within ♀** |
|  |  | Std. Deviation | 47.02 | 41.81 | 82.08 | 50.86 | 21.38 | 63.83 | WT vs KO | - | - |
|  |  | Minimum | 356.00 | 366.00 | 287.00 | 383.00 | 415.00 | 313.00 | HET vs KO | - | - |
|  |  | Maximum | 501.00 | 492.00 | 529.00 | 544.00 | 488.00 | 547.00 | WT vs HET | - | - |
|  |  | Range | 145.00 | 126.00 | 242.00 | 161.00 | 73.00 | 234.00 |  |  |  |
|  |  | Interquartile Range | 76.00 | 75.00 | 148.00 | 73.00 | 26.00 | 77.00 |  |  |  |

|  | **Variable** | **Statistic** | **WT**♂ | **IDE-HET** ♂ | **IDE-KO** ♂ | **WT** ♀ | **IDE-HET** ♀ | **IDE-KO** ♀ | **2-way ANOVA** | | |
| --- | --- | --- | --- | --- | --- | --- | --- | --- | --- | --- | --- |
|  | **Stereotypic time (s)** | Mean | 21.13 | 20.12 | 20.87 | 21.68 | 20.79 | 21.52 | **Genotype p-value** | **Sex**  **p-value** | **Interaction**  **p-value** |
|  |  | 95% C.I. Mean  Lower Bound  Upper Bound | 19.70 | 18.86 | 18.12 | 19.79 | 20.08 | 19.36 | 0.476 | 0.350 | 0.997 |
|  |  |  | 22.55 | 21.48 | 23.62 | 23.56 | 21.51 | 23.67 | **Post-hoc (Holm-Sidak) p-values** | | |
|  |  | Median | 21.46 | 20.89 | 21.15 | 21.86 | 20.74 | 21.95 | **within ♂** | **within ♂** | **within ♂** |
|  |  | Std. Deviation | 1.99 | 1.90 | 3.84 | 2.64 | 1.00 | 3.02 | WT vs KO | - | - |
|  |  | Minimum | 17.40 | 17.07 | 14.00 | 16.60 | 19.17 | 15.03 | HET vs KO | - | - |
|  |  | Maximum | 23.43 | 22.40 | 25.56 | 26.03 | 22.70 | 25.90 | WT vs HET | - | - |
|  |  | Range | 6.03 | 5.33 | 11.56 | 9.43 | 3.53 | 10.87 |  |  |  |
|  |  | Interquartile Range | 3.17 | 3.20 | 6.71 | 3.00 | 1.39 | 3.90 |  |  |  |
|  | **Time Center/Periphery** | Mean | 0.16 | 0.14 | 0.13 | 0.14 | 0.16 | 0.14 | **Genotype p-value** | **Sex**  **p-value** | **Interaction**  **p-value** |
|  |  | 95% C.I. Mean  Lower Bound  Upper Bound | 0.08 | 0.10 | 0.09 | 0.09 | 0.10 | 0.11 | 0.769 | 0.811 | 0.603 |
|  |  |  | 0.24 | 0.18 | 0.16 | 0.19 | 0.22 | 0.17 | **Post-hoc (Holm-Sidak) p-values** | | |
|  |  | Median | 0.12 | 0.12 | 0.15 | 0.12 | 0.14 | 0.15 | **Genotype** | **within ♂** | **within ♀** |
|  |  | Std. Deviation | 0.11 | 0.06 | 0.05 | 0.07 | 0.08 | 0.04 | WT vs KO | - | - |
|  |  | Minimum | 0.04 | 0.09 | 0.04 | 0.06 | 0.07 | 0.09 | HET vs KO | - | - |
|  |  | Maximum | 0.42 | 0.27 | 0.18 | 0.31 | 0.33 | 0.22 | WT vs HET | - | - |
|  |  | Range | 0.38 | 0.18 | 0.13 | 0.25 | 0.27 | 0.13 |  |  |  |
|  |  | Interquartile Range | 0.11 | 0.08 | 0.09 | 0.06 | 0.12 | 0.08 |  |  |  |

|  | **Variable** | **Statistic** | **WT**♂ | **IDE-HET** ♂ | **IDE-KO** ♂ | **WT** ♀ | **IDE-HET** ♀ | **IDE-KO** ♀ | **2-way ANOVA** | | |
| --- | --- | --- | --- | --- | --- | --- | --- | --- | --- | --- | --- |
|  | **Center entries** | Mean | 22.7 | 22.90 | 19.10 | 26.20 | 27.40 | 24.20 | **Genotype p-value** | **Sex**  **p-value** | **Interaction**  **p-value** |
|  |  | 95% C.I. Mean  Lower Bound  Upper Bound | 17.75 | 18.04 | 12.63 | 21.98 | 22.10 | 18.09 | 0.304 | 0.028* | 0.944 |
|  |  |  | 27.65 | 27.76 | 25.57 | 30.42 | 32.70 | 30.31 | **Post-hoc (Holm-Sidak) p-values** | | |
|  |  | Median | 22.50 | 23.50 | 23.00 | 26.50 | 24.50 | 23.50 | **Genotype** | **within ♂** | **within ♀** |
|  |  | Std. Deviation | 6.91 | 6.79 | 9.05 | 5.90 | 7.41 | 8.53 | WT vs KO | 0.289 | 0.554 |
|  |  | Minimum | 12.00 | 13.00 | 6.00 | 16.00 | 17.00 | 10.00 | HET vs KO | 0.263 | 0.345 |
|  |  | Maximum | 38.00 | 32.00 | 29.00 | 34.00 | 39.00 | 41.00 | WT vs HET | 0.953 | 0.722 |
|  |  | Range | 26.00 | 19.00 | 23.00 | 18.00 | 22.00 | 31.00 |  |  |  |
|  |  | Interquartile Range | 5.75 | 14.25 | 17.50 | 10.25 | 12.50 | 8.75 |  |  |  |
|  | **OLT** | Mean | -0.01 | -0.01 | 0.17 | 0.0 | -0.08 | -0.02 | **Genotype p-value** | **Sex**  **p-value** | **Interaction**  **p-value** |
|  |  | 95% C.I. Mean  Lower Bound  Upper Bound | -0.12 | -0.1407 | 0.05 | 0.00 | -0.18 | -0.12 | 0.052 | 0.094 | 0.024* |
|  |  |  | 0.10 | 0.13 | 0.29 | 0.13 | 0.01 | 0.07 | **Post-hoc (Holm-Sidak) p-values** | | |
|  |  | Median | -0.02 | -0.06 | 0.12 | 0.08 | -0.06 | -0.03 | **Genotype** | **within ♂** | **within ♀** |
|  |  | Std. Deviation | 0.16 | 0.19 | 0.17 | 0.09 | 0.14 | 0.14 | WT vs KO | 0.010* | 0.199 |
|  |  | Minimum | -0.25 | -0.20 | -0.02 | -0.08 | -0.38 | -0.26 | HET vs KO | 0.011* | 0.38 |
|  |  | Maximum | 0.21 | 0.46 | 0.47 | 0.22 | 0.14 | 0.18 | WT vs HET | 0.956 | 0.053 |
|  |  | Range | 0.46 | 0.66 | 0.49 | 0.30 | 0.52 | 0.44 |  |  |  |
|  |  | Interquartile Range | 0.29 | 0.15 | 0.32 | 0.14 | 0.13 | 0.21 |  |  |  |

|  | **Variable** | **Statistic** | **WT**♂ | **IDE-HET** ♂ | **IDE-KO** ♂ | **WT** ♀ | **IDE-HET** ♀ | **IDE-KO** ♀ | **2-way ANOVA** | | |
| --- | --- | --- | --- | --- | --- | --- | --- | --- | --- | --- | --- |
|  | **NORT** | Mean | 0.05 | 0.0 | 0.01 | 0.01 | 0.19 | 0.0 | **Genotype p-value** | **Sex**  **p-value** | **Interaction**  **p-value** |
|  |  | 95% C.I. Mean  Lower Bound  Upper Bound | -0.10 | -0.08 | -0.10 | -0.10 | 0.08 | -0.08 | 0.074 | 0.559 | 0.258 |
|  |  |  | 0.20 | 0.21 | 0.12 | 0.09 | 0.28 | 0.13 | **Post-hoc (Holm-Sidak) p-values** | | |
|  |  | Median | 0.10 | 0.14 | -0.02 | -0.02 | 0.16 | 0.08 | **Genotype** | **within ♂** | **within ♀** |
|  |  | Std. Deviation | 0.21 | 0,20 | 0.15 | 0.14 | 0.14 | 0.14 | WT vs KO | - | - |
|  |  | Minimum | -.46 | -0.29 | -0.20 | -0.31 | 0.00 | -0.30 | HET vs KO | - | - |
|  |  | Maximum | 0.22 | 0.29 | 0.27 | 0.17 | 0.46 | 0.15 | WT vs HET | - | - |
|  |  | Range | 0.68 | 0.59 | 0.47 | 0.48 | 0.46 | 0.45 |  |  |  |
|  |  | Interquartile Range | 0.22 | 0.38 | 0.27 | 0.17 | 0.16 | 0.15 |  |  |  |
|  | **Weight (g)** | Mean | 33.25 | 30.80 | 30.87 | 25.70 | 25.35 | 24.43 | **Genotype p-value** | **Sex**  **p-value** | **Interaction**  **p-value** |
|  |  | 95% C.I. Mean  Lower Bound  Upper Bound | 31.19 | 29.94 | 27.83 | 24.10 | 22.85 | 23.32 | 0.109 | <0.001* | 0.501 |
|  |  |  | 35.31 | 31.66 | 33.91 | 27.30 | 27.85 | 25.54 | **Post-hoc (Holm-Sidak) p-values** | | |
|  |  | Median | 33.50 | 30.45 | 30.05 | 25.20 | 24.30 | 24.50 | **Genotype** | **within ♂** | **within ♀** |
|  |  | Std. Deviation | 2.88 | 1.20 | 4.25 | 2.23 | 3.49 | 1.54 | WT vs KO | 0.063 | 0.316 |
|  |  | Minimum | 26.70 | 29.60 | 26.10 | 21.80 | 22.30 | 22.00 | HET vs KO | 0.956 | 0.467 |
|  |  | Maximum | 36.70 | 33.10 | 41.40 | 28.80 | 34.30 | 27.60 | WT vs HET | 0.056 | 0.782 |
|  |  | Range | 10.00 | 3.50 | 15.30 | 7.00 | 12.00 | 5.60 |  |  |  |
|  |  | Interquartile Range | 3.30 | 1.88 | 4.05 | 3.70 | 3.40 | 1.98 |  |  |  |

|  | **Variable** | **Statistic** | **WT**♂ | **IDE-HET** ♂ | **IDE-KO** ♂ | **WT** ♀ | **IDE-HET** ♀ | **IDE-KO** ♀ | **2-way ANOVA** | | |
| --- | --- | --- | --- | --- | --- | --- | --- | --- | --- | --- | --- |
|  | **Blood glucose (mg/dL)**  **(after 6h fasting)** | Mean | 137.10 | 120.20 | 132.50 | 115.90 | 108.90 | 121.00 | **Genotype p-value** | **Sex**  **p-value** | **Interaction**  **p-value** |
|  |  | 95% C.I. Mean  Lower Bound  Upper Bound | 125.46 | 109.09 | 110.09 | 102.03 | 101.06 | 104.18 | 0.110 | 0.008* | 0.686 |
|  |  |  | 148.74 | 131.31 | 154.91 | 129.77 | 116.74 | 137.82 | **Post-hoc (Holm-Sidak) p-values** | | |
|  |  | Median | 134.50 | 120.00 | 140.00 | 120.00 | 107.50 | 126.50 | **Genotype** | **within ♂** | **within ♀** |
|  |  | Std. Deviation | 16.27 | 15.53 | 31.33 | 19.39 | 10.96 | 23.52 | WT vs KO | 0.619 | 0.581 |
|  |  | Minimum | 118.00 | 99.00 | 88.00 | 91.00 | 93.00 | 90.00 | HET vs KO | 0.187 | 0.194 |
|  |  | Maximum | 172.00 | 150.00 | 188.00 | 149.00 | 128.00 | 147.00 | WT vs HET | 0.072 | 0.450 |
|  |  | Range | 54.00 | 51.00 | 100.00 | 58.00 | 35.00 | 57.00 |  |  |  |
|  |  | Interquartile Range | 21.00 | 25.00 | 46.00 | 34.00 | 17.00 | 45.00 |  |  |  |
|  | **Plasma insulin (ng/mL)**  **(after 6h fasting)** | Mean | 0.56 | 0.52 | 0.58 | 0.47 | 0.43 | 0.58 | **Genotype p-value** | **Sex**  **p-value** | **Interaction**  **p-value** |
|  |  | 95% C.I. Mean  Lower Bound  Upper Bound | 0.44 | 0.38 | 0.36 | 0.22 | 0.36 | 0.15 | 0.574 | 0.494 | 0.894 |
|  |  |  | 0.68 | 0.66 | 0.81 | 0.72 | 0.50 | 1.01 | **Post-hoc (Holm-Sidak) p-values** | | |
|  |  | Median | 0.49 | 0.47 | 0.47 | 0.39 | 0.44 | 0.44 | **Genotype** | **within ♂** | **within ♀** |
|  |  | Std. Deviation | 0.17 | 0.19 | 0.31 | 0.35 | 0.10 | 0.60 | WT vs KO | - | - |
|  |  | Minimum | 0.35 | 0.32 | 0.31 | 0.22 | 0.29 | 0.26 | HET vs KO | - | - |
|  |  | Maximum | 78,00 | 0.98 | 1.27 | 1.46 | 0.56 | 2.28 | WT vs HET | - | - |
|  |  | Range | 0.43 | 0.66 | 0.96 | 1.23 | 0.27 | 2.03 |  |  |  |
|  |  | Interquartile Range | 0.34 | 0.21 | 0.49 | 0.13 | 0.20 | 0.10 |  |  |  |

|  | **Variable** | **Statistic** | **WT**♂ | **IDE-HET** ♂ | **IDE-KO** ♂ | **WT** ♀ | **IDE-HET** ♀ | **IDE-KO** ♀ | **2-way ANOVA** | | |
| --- | --- | --- | --- | --- | --- | --- | --- | --- | --- | --- | --- |
|  | **Insulin Receptor** | Mean | 102.75 | 136.33 | 124.12 | 48.72 | 54.98 | 57.42 | **Genotype p-value** | **Sex**  **p-value** | **Interaction**  **p-value** |
|  |  | 95% C.I. Mean  Lower Bound  Upper Bound | 85.96 | 92.45 | 87.78 | 33.15 | 36.68 | 29.86 | 0.265 | <0.001* | 0.558 |
|  |  |  | 119.54 | 180.22 | 160.46 | 64.29 | 73.28 | 84.99 | **Post-hoc (Holm-Sidak) p-values** | | |
|  |  | Median | 98.74 | 116.37 | 122.59 | 42.61 | 48.00 | 37.17 | **Genotype** | **within ♂** | **within ♀** |
|  |  | Std. Deviation | 23.47 | 61.34 | 50.80 | 21.76 | 25.58 | 38.53 | WT vs KO | 0.235 | 0.627 |
|  |  | Minimum | 65.69 | 66.73 | 54.23 | 20.44 | 27.25 | 18.5 | HET vs KO | 0.496 | 0.891 |
|  |  | Maximum | 137.60 | 263.94 | 226,43 | 91.68 | 111.90 | 132.88 | WT vs HET | 0.065 | 0.726 |
|  |  | Range | 71.91 | 197.21 | 172.19 | 71.24 | 84.66 | 114.38 |  |  |  |
|  |  | Interquartile Range | 33.09 | 84.18 | 74.80 | 26.99 | 34.09 | 61.79 |  |  |  |
|  | **AKT1** | Mean | 105.55 | 115.97 | 77.71 | 55.24 | 61.08 | 59.82 | **Genotype p-value** | **Sex**  **p-value** | **Interaction**  **p-value** |
|  |  | 95% C.I. Mean  Lower Bound  Upper Bound | 53.39 | 57.58 | 41.94 | 36.65 | 42.74 | 35.48 | 0.502 | 0.004* | 0.492 |
|  |  |  | 157.70 | 174.37 | 113.48 | 73.84 | 79.43 | 84.16 | **Post-hoc (Holm-Sidak) p-values** | | |
|  |  | Median | 76.37 | 70.49 | 67.09 | 53.97 | 51.66 | 46.84 | **Genotype** | **within ♂** | **within ♀** |
|  |  | Std. Deviation | 72.91 | 81.63 | 50.00 | 25.99 | 25.65 | 34.03 | WT vs KO | 0.247 | 0.848 |
|  |  | Minimum | 34.62 | 41.53 | 21.48 | 20.57 | 29.33 | 25.83 | HET vs KO | 0.113 | 0.958 |
|  |  | Maximum | 223.12 | 276.73 | 173.27 | 102.59 | 108.05 | 122.01 | WT vs HET | 0.663 | 0.807 |
|  |  | Range | 188.51 | 235.20 | 151.79 | 82.02 | 78.73 | 96.19 |  |  |  |
|  |  | Interquartile Range | 147.99 | 130.71 | 84.34 | 38.32 | 23.89 | 55.57 |  |  |  |

|  | **Variable** | **Statistic** | **WT**♂ | **IDE-HET** ♂ | **IDE-KO** ♂ | **WT** ♀ | **IDE-HET** ♀ | **IDE-KO** ♀ | **2-way ANOVA** | | |
| --- | --- | --- | --- | --- | --- | --- | --- | --- | --- | --- | --- |
|  | **IDE** | Mean | 102.88 | 88.03 | 0.00 | 71.16 | 43.05 | 0.00 | **Genotype p-value** | **Sex**  **p-value** | **Interaction**  **p-value** |
|  |  | 95% C.I. Mean  Lower Bound  Upper Bound | 61.06 | 40.97 | 0.00 | 34.04 | 14.66 | 0.00 | <0.001* | 0.031* | 0.272 |
|  |  |  | 144.69 | 135.09 | 0.00 | 108.29 | 71.44 | 0.00 | **Post-hoc (Holm-Sidak) p-values** | | |
|  |  | Median | 106.02 | 68.82 | 0.00 | 65.22 | 29.04 | 0.00 | **Genotype** | **within ♂** | **within ♀** |
|  |  | Std. Deviation | 58.45 | 65.79 | 0.00 | 51.90 | 39.68 | 0.00 | WT vs KO | <0.001* | <0.001* |
|  |  | Minimum | 31.06 | 26.63 | 0.00 | 3.84 | 2.57 | 0.00 | HET vs KO | <0.001* | 0.036* |
|  |  | Maximum | 205.13 | 223.57 | 0.00 | 160.66 | 116.49 | 0.00 | WT vs HET | 0.461 | 0.166 |
|  |  | Range | 174.07 | 196.94 | 0.00 | 156.82 | 113.92 | 0.00 |  |  |  |
|  |  | Interquartile Range | 101.06 | 108.88 | 0.00 | 83.99 | 46.66 | 0.00 |  |  |  |
|  | **CD11b** | Mean | 112.81 | 123.92 | 102.31 | 248.02 | 300.75 | 246.47 | **Genotype p-value** | **Sex**  **p-value** | **Interaction**  **p-value** |
|  |  | 95% C.I. Mean  Lower Bound  Upper Bound | 75.11 | 83.66 | 51.95 | 109.22 | 96.23 | 91.19 | 0.756 | 0.001* | 0.922 |
|  |  |  | 150.51 | 164.18 | 152.67 | 386.83 | 505.26 | 401.76 | **Post-hoc (Holm-Sidak) p-values** | | |
|  |  | Median | 100.71 | 103.73 | 79.96 | 183.30 | 207.83 | 148.02 | **Genotype** | **within ♂** | **within ♀** |
|  |  | Std. Deviation | 52.70 | 56.27 | 70.40 | 194.04 | 285.89 | 217.07 | WT vs KO | 0.892 | 0.984 |
|  |  | Minimum | 76.05 | 74.90 | 43.94 | 59.54 | 78.70 | 77.14 | HET vs KO | 0.780 | 0.483 |
|  |  | Maximum | 254.40 | 236.50 | 277.28 | 646.91 | 967.38 | 672.98 | WT vs HET | 0.886 | 0.496 |
|  |  | Range | 178.35 | 161.61 | 233.34 | 587.37 | 888.68 | 595.84 |  |  |  |
|  |  | Interquartile Range | 36.47 | 58.47 | 56.13 | 265.84 | 211.79 | 216.74 |  |  |  |

|  | **Variable** | **Statistic** | **WT**♂ | **IDE-HET** ♂ | **IDE-KO** ♂ | **WT** ♀ | **IDE-HET** ♀ | **IDE-KO** ♀ | **2-way ANOVA** | | |
| --- | --- | --- | --- | --- | --- | --- | --- | --- | --- | --- | --- |
|  | **Iba1** | Mean | 72.22 | 39.10 | 64.18 | 70.48 | 73.21 | 62.65 | **Genotype p-value** | **Sex**  **p-value** | **Interaction**  **p-value** |
|  |  | 95% C.I. Mean  Lower Bound  Upper Bound | 14.21 | 19.54 | 1.43 | 27.83 | 18.95 | 31.01 | 0.769 | 0.550 | 0.618 |
|  |  |  | 130.23 | 58.66 | 126.94 | 113.13 | 127.47 | 94.28 | **Post-hoc (Holm-Sidak) p-values** | | |
|  |  | Median | 37.81 | 43.65 | 35.68 | 56.09 | 50.75 | 53.29 | **Genotype** | **within ♂** | **within ♀** |
|  |  | Std. Deviation | 81.10 | 27.35 | 87.72 | 59.62 | 75.85 | 44.22 | WT vs KO | - | - |
|  |  | Minimum | 3.07 | 3.50 | 2.87 | 9.53 | 17.78 | 8.13 | HET vs KO | - | - |
|  |  | Maximum | 238.06 | 81.80 | 295.22 | 191.63 | 266.52 | 148.79 | WT vs HET | - | - |
|  |  | Range | 234.99 | 78.30 | 292.35 | 182.10 | 248.75 | 140.66 |  |  |  |
|  |  | Interquartile Range | 132.94 | 53.47 | 75.54 | 95.39 | 67.96 | 59.14 |  |  |  |
|  | **GFAP** | Mean | 64.78 | 30.12 | 41.75 | 207.23 | 208.02 | 192.23 | **Genotype p-value** | **Sex**  **p-value** | **Interaction**  **p-value** |
|  |  | 95% C.I. Mean  Lower Bound  Upper Bound | 5.33 | 12.45 | 6.63 | 43.07 | 56.35 | 52.08 | 0.915 | <0.001* | 0.932 |
|  |  |  | 124.23 | 47.79 | 76.87 | 371.39 | 359.68 | 332.39 | **Post-hoc (Holm-Sidak) p-values** | | |
|  |  | Median | 23.18 | 25.94 | 11.34 | 141.54 | 124.63 | 135.70 | **Genotype** | **within ♂** | **within ♀** |
|  |  | Std. Deviation | 83.11 | 24.70 | 49.09 | 229.48 | 212.01 | 195.92 | WT vs KO | 0.743 | 0.831 |
|  |  | Minimum | 1.75 | 2.50 | 2.13 | 12.96 | 32.43 | 39.68 | HET vs KO | 0.868 | 0.822 |
|  |  | Maximum | 234.18 | 68.74 | 138.36 | 666.16 | 660.62 | 569.44 | WT vs HET | 0.621 | 0.991 |
|  |  | Range | 232.42 | 66.24 | 136.23 | 653.20 | 628.19 | 529.76 |  |  |  |
|  |  | Interquartile Range | 110.26 | 42.20 | 81.89 | 305.80 | 252.99 | 211.28 |  |  |  |

|  | **Variable** | **Statistic** | **WT**♂ | **IDE-HET** ♂ | **IDE-KO** ♂ | **WT** ♀ | **IDE-HET** ♀ | **IDE-KO** ♀ | **2-way ANOVA** | | |
| --- | --- | --- | --- | --- | --- | --- | --- | --- | --- | --- | --- |
|  | **ApoD** | Mean | 93.36 | 91.95 | 81.28 | 59.12 | 58.74 | 48.17 | **Genotype p-value** | **Sex**  **p-value** | **Interaction**  **p-value** |
|  |  | 95% C.I. Mean  Lower Bound  Upper Bound | 59.71 | 55.35 | 37.40 | 37.95 | 44.86 | 34.52 | 0.620 | 0.003* | 0.999 |
|  |  |  | 127.01 | 128.56 | 125.16 | 80.29 | 72.62 | 61.83 | **Post-hoc (Holm-Sidak) p-values** | | |
|  |  | Median | 107.20 | 109.44 | 69.79 | 52.26 | 53.22 | 39.61 | **Genotype** | **within ♂** | **within ♀** |
|  |  | Std. Deviation | 47.04 | 51.17 | 61.34 | 29.60 | 19.40 | 19.09 | WT vs KO | 0.516 | 0.555 |
|  |  | Minimum | 17.45 | 17.69 | 6.87 | 28.23 | 38.46 | 31.60 | HET vs KO | 0.565 | 0.569 |
|  |  | Maximum | 166.89 | 164.41 | 188.83 | 113.84 | 93.18 | 91.27 | WT vs HET | 0.940 | 0.984 |
|  |  | Range | 149.45 | 146.72 | 181.96 | 85.62 | 54.72 | 59.67 |  |  |  |
|  |  | Interquartile Range | 60.56 | 93.49 | 83.64 | 46.70 | 32.28 | 25.05 |  |  |  |
|  | **Aβ** | Mean | 88.98 | 89.60 | 85.19 | 53.29 | 58.92 | 44.62 | **Genotype p-value** | **Sex**  **p-value** | **Interaction**  **p-value** |
|  |  | 95% C.I. Mean  Lower Bound  Upper Bound | 46.61 | 58.32 | 47.41 | 0.39 | 6.47 | 8.27 | 0.822 | 0.025* | 0.967 |
|  |  |  | 131.36 | 120.87 | 122.97 | 106.19 | 111.36 | 80.97 | **Post-hoc (Holm-Sidak) p-values** | | |
|  |  | Median | 82.74 | 94.65 | 65.56 | 25.17 | 25.13 | 22.20 | **Genotype** | **within ♂** | **within ♀** |
|  |  | Std. Deviation | 59.23 | 43.72 | 52.81 | 73.95 | 73.32 | 50.81 | WT vs KO | 0.888 | 0.748 |
|  |  | Minimum | 21.08 | 25.03 | 24.23 | 4.51 | 11.43 | 6.71 | HET vs KO | 0.87 | 0.597 |
|  |  | Maximum | 204.28 | 165.01 | 178.58 | 219.74 | 213.96 | 147.78 | WT vs HET | 0.982 | 0.835 |
|  |  | Range | 183.21 | 139.98 | 154.35 | 215.23 | 202.52 | 141.07 |  |  |  |
|  |  | Interquartile Range | 96.83 | 76.23 | 88.56 | 52.78 | 75.06 | 40.88 |  |  |  |

**Table S1.2. Multinomial logistic regression model.** *WT genotype was set as the reference category. Predictors for the classification of genotype are shown with their coefficients and statistical significance. Residual deviance: 28.52. AIC: 100.52 (*, p<0.05; **, p<0.01; ***, p<0.001)*

| **Multinomial Logistic Regression Model (Reference = WT)** | | | | | |
| --- | --- | --- | --- | --- | --- |
| **Genotype** | **Variable** | **Coefficient** | **Std. Error** | **P-value** | **Significance** |
| IDE-HET | (Intercept) | 53.60 | 1.46 | 0.000 | *** |
| IDE-HET | Sex (F) | 0.53 | 2.23 | 0.813 |  |
| IDE-HET | Ambulatory episodes | -0.08 | 0.07 | 0.265 |  |
| IDE-HET | Stereotypic counts | -0.02 | 0.02 | 0.223 |  |
| IDE-HET | Relative time (C/P) | -17.01 | 1.42 | 0.000 | *** |
| IDE-HET | OLT | -15.32 | 4.35 | 0.000 | *** |
| IDE-HET | NORT | -1.56 | 3.92 | 0.690 |  |
| IDE-HET | Weight | -0.77 | 0.17 | 0.000 | *** |
| IDE-HET | Blood glucose | -0.07 | 0.04 | 0.047 | * |
| IDE-HET | Plasma insulin | -6.94 | 4.41 | 0.115 |  |
| IDE-HET | InsR | 0.02 | 0.02 | 0.386 |  |
| IDE-HET | AKT | -0.01 | 0.02 | 0.761 |  |
| IDE-HET | IDE | 0.00 | 0.02 | 0.849 |  |
| IDE-HET | CD11b | 0.00 | 0.01 | 0.744 |  |
| IDE-HET | Iba1 | 0.02 | 0.02 | 0.348 |  |
| IDE-HET | GFAP | -0.04 | 0.02 | 0.049 | * |
| IDE-HET | ApoD | -0.03 | 0.03 | 0.290 |  |
| IDE-HET | Aβ | 0.05 | 0.04 | 0.192 |  |
| IDE-KO | (Intercept) | -6.43 | 0.00 | 0.000 | *** |
| IDE-KO | Sex (F) | -8.72 | 0.04 | 0.000 | *** |
| IDE-KO | Ambulatory episodes | -0.71 | 0.54 | 0.186 |  |
| IDE-KO | Stereotypic counts | 0.14 | 0.99 | 0.890 |  |
| IDE-KO | Relative time (C/P) | -17.73 | 0.00 | 0.000 | *** |
| IDE-KO | OLT | 12.15 | 0.01 | 0.000 | *** |
| IDE-KO | NORT | -27.91 | 0.01 | 0.000 | *** |
| IDE-KO | Weight | -1.21 | 0.60 | 0.045 | * |
| IDE-KO | Blood glucose | 0.28 | 1.75 | 0.873 |  |
| IDE-KO | Plasma insulin | -8.65 | 0.01 | 0.000 | *** |
| IDE-KO | InsR | 0.20 | 2.11 | 0.926 |  |
| IDE-KO | AKT | -0.72 | 0.93 | 0.438 |  |
| IDE-KO | IDE | -1.50 | 0.75 | 0.046 | * |
| IDE-KO | CD11b | 0.11 | 2.65 | 0.967 |  |
| IDE-KO | Iba1 | -0.04 | 1.20 | 0.971 |  |
| IDE-KO | GFAP | 0.12 | 2.24 | 0.959 |  |
| IDE-KO | ApoD | 0.47 | 1.59 | 0.766 |  |
| IDE-KO | Aβ | 0.26 | 1.56 | 0.868 |  |
