# Supplementary material for: Insulin-degrading enzyme (IDE) as a modulator of microglial phenotypes in the context of Alzheimer’s disease and brain aging
Source: J Neuroinflammation. 2023 Oct 11;20:233. doi: 10.1186/s12974-023-02914-7 (PMC10566021; doi:10.1186/s12974-023-02914-7)
Supplement: Supplementary file 2 — Additional file 2: File S2. Transcriptomic profiling of IDE-KO vs. WT primary microglia. [file 12974_2023_2914_MOESM2_ESM.docx]

**Insulin-degrading enzyme (IDE) as a modulator of microglial phenotypes in the context of Alzheimer’s disease and brain aging.**

**Miriam Corraliza-Gomez, Teresa Bermejo, Jingtao Lilue, Noelia Rodriguez-Iglesias, Jorge Valero, Irene Cozar-Castellano, Eduardo Arranz, Diego Sanchez and Maria Dolores Ganfornina**

**Supplementary File 2.** **Transcriptomic profiling of IDE-KO vs WT primary microglia.**

**Table S2.1. List of genes differentially expressed in IDE-KO microglia vs WT microglia.** *Only genes with FDR<0.05 and absolute log2 fold change ≥ 1 are included.*

| Gene | Description | log2 FoldChange | Fold Change | FDR | Ensembl ID |
| --- | --- | --- | --- | --- | --- |
| Slfn1 | Schlafen 1 | 5.03 | 32.56 | 2.76E-09 | ENSMUSG00000078763 |
| Ccl7 | Chemokine (C-C motif) ligand 7 | 3.69 | 12.95 | 4.03E-138 | ENSMUSG00000035373 |
| Ms4a4c | Membrane-spanning 4-domains, subfamily A, member 4C | 3.55 | 11.69 | 3.89E-19 | ENSMUSG00000024675 |
| Ccl5 | Chemokine (C-C motif) ligand 5 | 3.23 | 9.38 | 5.97E-105 | ENSMUSG00000035042 |
| Lcn2 | Lipocalin 2 | 3.21 | 9.28 | 9.89E-05 | ENSMUSG00000026822 |
| Il1b | Interleukin 1 beta | 2.96 | 7.77 | 7.97E-11 | ENSMUSG00000027398 |
| Gm6545 | Predicted gene 6545 | 2.91 | 7.53 | 4.53E-15 | ENSMUSG00000111118 |
| Ccl8 | Chemokine (C-C motif) ligand 8 | 2.76 | 6.80 | 4.65E-34 | ENSMUSG00000009185 |
| Iigp1 | Interferon inducible GTPase 1 | 2.54 | 5.82 | 9.46E-12 | ENSMUSG00000054072 |
| H2-Q7 | Histocompatibility 2, Q region locus 7 | 2.46 | 5.50 | 5.37E-23 | ENSMUSG00000060550 |
| Slc13a3 | Solute carrier family 13 (sodium-dependent dicarboxylate transporter), member 3 | 2.43 | 5.39 | 6.60E-09 | ENSMUSG00000018459 |
| Ifi208 | Interferon activated gene 208 | 2.25 | 4.75 | 2.22E-16 | ENSMUSG00000066677 |
| Fgl2 | Fibrinogen-like protein 2 | 2.19 | 4.57 | 8.38E-17 | ENSMUSG00000039899 |
| Oasl1 | 2'-5' oligoadenylate synthetase-like 1 | 2.03 | 4.09 | 1.87E-08 | ENSMUSG00000041827 |
| BC147527 | cDNA sequence BC147527 | 2.01 | 4.03 | 1.15E-03 | ENSMUSG00000094796 |
| Gm1966 | Predicted gene 1966 | 1.96 | 3.90 | 1.92E-04 | ENSMUSG00000073902 |
| Clec4a1 | C-type lectin domain family 4, member a1 | 1.88 | 3.67 | 1.63E-40 | ENSMUSG00000049037 |
| Fcrls | Fc receptor-like S, scavenger receptor | 1.79 | 3.45 | 4.28E-34 | ENSMUSG00000015852 |
| Slco2b1 | Solute carrier organic anion transporter family, member 2b1 | 1.78 | 3.43 | 7.58E-18 | ENSMUSG00000030737 |
| Gbp5 | Guanylate binding protein 5 | 1.74 | 3.34 | 1.08E-02 | ENSMUSG00000105504 |
| Ifi205 | Interferon activated gene 205 | 1.73 | 3.32 | 6.15E-07 | ENSMUSG00000054203 |
| Irf7 | Interferon regulatory factor 7 | 1.72 | 3.30 | 1.25E-37 | ENSMUSG00000025498 |
| Nav3 | Neuron navigator 3 | 1.64 | 3.11 | 1.15E-03 | ENSMUSG00000020181 |
| Ifi209 | Interferon activated gene 209 | 1.57 | 2.96 | 3.76E-23 | ENSMUSG00000043263 |
| Ifi206 | Interferon activated gene 206 | 1.54 | 2.90 | 5.93E-13 | ENSMUSG00000037849 |
| Ifi213 | Interferon activated gene 213 | 1.54 | 2.90 | 2.41E-09 | ENSMUSG00000073491 |
| Slamf7 | SLAM family member 7 | 1.51 | 2.84 | 4.10E-08 | ENSMUSG00000038179 |
| Atf5 | Activating transcription factor 5 | 1.47 | 2.77 | 1.57E-15 | ENSMUSG00000038539 |
| G530011O06Rik | RIKEN cDNA G530011O06 gene | 1.46 | 2.74 | 1.81E-12 | ENSMUSG00000072844 |
| Ccr5 | Chemokine (C-C motif) receptor 5 | 1.42 | 2.68 | 2.78E-20 | ENSMUSG00000079227 |
| Mx1 | MX dynamin-like GTPase 1 | 1.42 | 2.67 | 1.81E-12 | ENSMUSG00000000386 |
| Ifi44 | Interferon-induced protein 44 | 1.41 | 2.66 | 5.21E-05 | ENSMUSG00000028037 |
| Ccl12 | Chemokine (C-C motif) ligand 12 | 1.41 | 2.66 | 5.11E-31 | ENSMUSG00000035352 |
| Zbp1 | Z-DNA binding protein 1 | 1.36 | 2.57 | 1.30E-15 | ENSMUSG00000027514 |
| Ifi211 | Interferon activated gene 211 | 1.33 | 2.52 | 4.16E-20 | ENSMUSG00000026536 |
| Phf11a | PHD finger protein 11A | 1.33 | 2.51 | 3.44E-07 | ENSMUSG00000044703 |
| Phf11b | PHD finger protein 11B | 1.29 | 2.45 | 3.07E-22 | ENSMUSG00000091649 |
| Socs3 | Suppressor of cytokine signaling 3 | 1.26 | 2.40 | 2.11E-04 | ENSMUSG00000053113 |
| Phf11d | PHD finger protein 11D | 1.26 | 2.39 | 8.72E-17 | ENSMUSG00000068245 |
| Ifit2 | Interferon-induced protein with tetratricopeptide repeats 2 | 1.24 | 2.37 | 2.53E-10 | ENSMUSG00000045932 |
| Ifit3 | Interferon-induced protein with tetratricopeptide repeats 3 | 1.24 | 2.35 | 1.99E-13 | ENSMUSG00000074896 |
| Bcl3 | B cell leukemia/lymphoma 3 | 1.23 | 2.35 | 1.52E-05 | ENSMUSG00000053175 |
| Clec4a3 | C-type lectin domain family 4, member a3 | 1.22 | 2.33 | 1.75E-09 | ENSMUSG00000043832 |
| AW112010 | Expressed sequence AW112010 | 1.21 | 2.32 | 5.26E-04 | ENSMUSG00000075010 |
| Ms4a6b | Membrane-spanning 4-domains, subfamily A, member 6B | 1.17 | 2.25 | 1.65E-17 | ENSMUSG00000024677 |
| Egr1 | Early growth response 1 | 1.12 | 2.18 | 3.62E-04 | ENSMUSG00000038418 |
| Tap1 | Transporter 1, ATP-binding cassette, sub-family B (MDR/TAP) | 1.12 | 2.18 | 6.09E-09 | ENSMUSG00000037321 |
| Zfp36 | Zinc finger protein 36 | 1.11 | 2.16 | 4.39E-10 | ENSMUSG00000044786 |
| Ly6e | Lymphocyte antigen 6 complex, locus E | 1.10 | 2.15 | 7.51E-16 | ENSMUSG00000022587 |
| Ifi47 | Interferon gamma inducible protein 47 | 1.10 | 2.15 | 4.81E-10 | ENSMUSG00000078920 |
| Ifi204 | Interferon activated gene 204 | 1.07 | 2.10 | 1.97E-14 | ENSMUSG00000073489 |
| Ptger4 | Prostaglandin E receptor 4 (subtype EP4) | 1.05 | 2.07 | 4.07E-07 | ENSMUSG00000039942 |
| Isg20 | Interferon-stimulated protein | 1.05 | 2.07 | 2.28E-03 | ENSMUSG00000039236 |
| Gbp7 | Guanylate binding protein 7 | 1.04 | 2.06 | 2.99E-04 | ENSMUSG00000040253 |
| Nfkbiz | Nuclear factor of kappa light polypeptide gene enhancer in B cells inhibitor, zeta | 1.04 | 2.06 | 7.67E-06 | ENSMUSG00000035356 |
| Oas3 | 2'-5' oligoadenylate synthetase-like 3 | 1.04 | 2.06 | 1.01E-08 | ENSMUSG00000032661 |
| Eps8 | Epidermal growth factor receptor pathway substrate 8 | 1.03 | 2.05 | 3.75E-02 | ENSMUSG00000015766 |
| Traf1 | TNF receptor-associated factor 1 | 1.03 | 2.04 | 8.57E-03 | ENSMUSG00000026875 |
| Fcgr1 | Fc receptor, IgG, high affinity I | 1.02 | 2.03 | 8.99E-14 | ENSMUSG00000015947 |
| Lrrc27 | Leucine rich repeat containing 27 | -1.00 | 0.50 | 3.57E-03 | ENSMUSG00000015980 |
| Cdkn2a | Cyclin dependent kinase inhibitor 2A | -1.00 | 0.50 | 4.54E-13 | ENSMUSG00000044303 |
| Rraga | Ras-related GTP binding A | -1.01 | 0.50 | 9.02E-11 | ENSMUSG00000070934 |
| Tgm2 | Transglutaminase 2, C polypeptide | -1.01 | 0.50 | 8.85E-04 | ENSMUSG00000037820 |
| St3gal5 | ST3 beta-galactoside alpha-2,3-sialyltransferase 5 | -1.03 | 0.49 | 1.61E-11 | ENSMUSG00000056091 |
| Ralgapa2 | Ral GTPase activating protein, alpha subunit 2 (catalytic) | -1.04 | 0.48 | 2.38E-05 | ENSMUSG00000037110 |
| Emp1 | Epithelial membrane protein 1 | -1.07 | 0.48 | 5.36E-03 | ENSMUSG00000030208 |
| L1cam | L1 cell adhesion molecule | -1.09 | 0.47 | 1.82E-02 | ENSMUSG00000031391 |
| Pdxk | Pyridoxal (pyridoxine, vitamin B6) kinase | -1.10 | 0.47 | 4.97E-07 | ENSMUSG00000032788 |
| Pls3 | Plastin 3 (T-isoform) | -1.10 | 0.47 | 1.39E-05 | ENSMUSG00000016382 |
| Sgsh | N-sulfoglucosamine sulfohydrolase (sulfamidase) | -1.13 | 0.46 | 1.30E-05 | ENSMUSG00000005043 |
| Cd28 | CD28 antigen | -1.15 | 0.45 | 1.30E-03 | ENSMUSG00000026012 |
| Cd9 | CD9 antigen | -1.15 | 0.45 | 4.42E-16 | ENSMUSG00000030342 |
| Anxa1 | Annexin A1 | -1.16 | 0.45 | 2.05E-09 | ENSMUSG00000024659 |
| Anpep | Alanyl (membrane) aminopeptidase | -1.17 | 0.44 | 3.96E-05 | ENSMUSG00000039062 |
| Alox5 | Arachidonate 5-lipoxygenase | -1.19 | 0.44 | 6.16E-06 | ENSMUSG00000025701 |
| Lrrc17 | Leucine rich repeat containing 17 | -1.20 | 0.44 | 1.87E-11 | ENSMUSG00000039883 |
| Ahnak | AHNAK nucleoprotein (desmoyokin) | -1.25 | 0.42 | 1.37E-06 | ENSMUSG00000069833 |
| Il1rn | Interleukin 1 receptor antagonist | -1.29 | 0.41 | 1.99E-13 | ENSMUSG00000026981 |
| Slc7a2 | Solute carrier family 7 (cationic amino acid transporter, y+ system), member 2 | -1.29 | 0.41 | 7.60E-03 | ENSMUSG00000031596 |
| Fabp4 | Fatty acid binding protein 4, adipocyte | -1.33 | 0.40 | 9.64E-03 | ENSMUSG00000062515 |
| Bhlhe40 | Basic helix-loop-helix family, member e40 | -1.35 | 0.39 | 1.72E-05 | ENSMUSG00000030103 |
| Marveld1 | MARVEL (membrane-associating) domain containing 1 | -1.37 | 0.39 | 3.09E-04 | ENSMUSG00000044345 |
| Ldlrad3 | Low density lipoprotein receptor class A domain containing 3 | -1.37 | 0.39 | 1.72E-02 | ENSMUSG00000048058 |
| Emilin2 | Elastin microfibril interfacer 2 | -1.38 | 0.38 | 1.27E-08 | ENSMUSG00000024053 |
| Chchd10 | Coiled-coil-helix-coiled-coil-helix domain containing 10 | -1.39 | 0.38 | 1.96E-04 | ENSMUSG00000049422 |
| Abcb4 | ATP-binding cassette, sub-family B (MDR/TAP), member 4 | -1.39 | 0.38 | 5.56E-12 | ENSMUSG00000042476 |
| Gask1b | Golgi associated kinase 1B | -1.40 | 0.38 | 6.78E-03 | ENSMUSG00000027955 |
| Atp8b4 | ATPase, class I, type 8B, member 4 | -1.42 | 0.37 | 2.38E-07 | ENSMUSG00000060131 |
| Hgsnat | Heparan-alpha-glucosaminide N-acetyltransferase | -1.44 | 0.37 | 1.55E-19 | ENSMUSG00000037260 |
| Gm14221 | Predicted gene 14221 | -1.49 | 0.36 | 2.16E-03 | ENSMUSG00000078956 |
| Gpnmb | Glycoprotein (transmembrane) nmb | -1.49 | 0.35 | 2.60E-31 | ENSMUSG00000029816 |
| Tmem158 | Transmembrane protein 158 | -1.53 | 0.35 | 3.66E-02 | ENSMUSG00000054871 |
| Gcm2 | Glial cells missing homolog 2 | -1.60 | 0.33 | 4.20E-02 | ENSMUSG00000021362 |
| Alox15 | Arachidonate 15-lipoxygenase | -1.65 | 0.32 | 2.78E-03 | ENSMUSG00000018924 |
| S100a6 | S100 calcium binding protein A6 (calcyclin) | -1.80 | 0.29 | 3.92E-27 | ENSMUSG00000001025 |
| Dab2ip | Disabled 2 interacting protein | -1.81 | 0.29 | 3.26E-03 | ENSMUSG00000026883 |
| Tnfsf8 | Tumor necrosis factor (ligand) superfamily, member 8 | -2.02 | 0.25 | 1.64E-02 | ENSMUSG00000028362 |
| Adgre5 | Adhesion G protein-coupled receptor E5 | -2.04 | 0.24 | 5.88E-03 | ENSMUSG00000002885 |
| S100a4 | S100 calcium binding protein A4 | -2.06 | 0.24 | 3.71E-08 | ENSMUSG00000001020 |
| Timd4 | T cell immunoglobulin and mucin domain containing 4 | -2.25 | 0.21 | 2.71E-04 | ENSMUSG00000055546 |
| Ide | Insulin-degrading enzyme | -2.39 | 0.19 | 1.79E-18 | ENSMUSG00000056999 |
| Csta2 | Cystatin A family member 2 | -2.48 | 0.18 | 3.61E-20 | ENSMUSG00000095620 |
| Fcna | Ficolin A | -2.54 | 0.17 | 9.46E-12 | ENSMUSG00000026938 |

**Table S2.2. Gene ontology enrichment analysis on the upregulated genes in IDE-KO vs WT microglia**

|  | **Category** | **ID** | **Description** | **P-adj. value** | **Term Size** | **Gene ID** | **Gene**  **Ratio** | **Bg**  **Ratio** |
| --- | --- | --- | --- | --- | --- | --- | --- | --- |
| **GO:0006952** | GO:BP | GO:0006952 | defense response | 7.89e-16 | 1742 | Ccl7/Ccl5/Irf7/Ccl8/Ccr5/Fgl2/Zbp1/Ifit3/Mx1/Il1b/Ifit2/Zfp36/Tap1/Oas3/Slamf7/Ptger4/Nfkbiz/ Bcl3/Lcn2/Socs3/Gbp7/Isg20/Gbp5 | 23/31 | 1742/21092 |
| **GO:0044419** | GO:BP | GO:0044419 | biological process involved in interspecies interaction between organisms | 9.86e-15 | 1675 | Ccl7/Ccl5/Irf7/Ccl8/Ccr5/Fgl2/Ly6e/Zbp1/Ifit3/ Mx1/Il1b/Ifit2/Zfp36/Oas3/Slamf7/Ptger4/Bcl3/ Ifi44/Lcn2/Gbp7/Isg20/Gbp5 | 22/31 | 1675/21092 |
| **GO:0071345** | GO:BP | GO:0071345 | cellular response to cytokine stimulus | 1.62e-14 | 817 | Ccl7/Ccl5/Irf7/Ccl8/Ccr5/Zbp1/Ifit3/Mx1/Il1b/Ifit2/Zfp36/Oas3/Socs3/Gbp7/Egr1/Traf1/Gbp5/Eps8 | 18/32 | 817/21092 |
| **GO:0051707** | GO:BP | GO:0051707 | response to other organism | 3.22e-14 | 1513 | Ccl7/Ccl5/Irf7/Ccl8/Ccr5/Fgl2/Zbp1/Ifit3/Mx1/ Il1b/Ifit2/Zfp36/Oas3/Slamf7/Ptger4/Bcl3/Ifi44/ Lcn2/Gbp7/Isg20/Gbp5 | 21/31 | 1513/21092 |
| **GO:0043207** | GO:BP | GO:0043207 | response to external biotic stimulus | 3.36e-14 | 1516 | Ccl7/Ccl5/Irf7/Ccl8/Ccr5/Fgl2/Zbp1/Ifit3/Mx1/ Il1b/Ifit2/Zfp36/Oas3/Slamf7/Ptger4/Bcl3/Ifi44/ Lcn2/Gbp7/Isg20/Gbp5 | 21/31 | 1516/21092 |
| **GO:0009607** | GO:BP | GO:0009607 | response to biotic stimulus | 5.56e-14 | 1554 | Ccl7/Ccl5/Irf7/Ccl8/Ccr5/Fgl2/Zbp1/Ifit3/Mx1/ Il1b/Ifit2/Zfp36/Oas3/Slamf7/Ptger4/Bcl3/Ifi44/ Lcn2/Gbp7/Isg20/Gbp5 | 21/31 | 1554/21092 |
| **GO:0009615** | GO:BP | GO:0009615 | response to virus | 7.37e-14 | 403 | Ccl5/Irf7/Ccl8/Fgl2/Zbp1/Ifit3/Mx1/Il1b/Ifit2/ Oas3/Bcl3/Ifi44/Gbp7/Isg20 | 14/29 | 403/21092 |
| **GO:0034097** | GO:BP | GO:0034097 | response to cytokine | 1.00e-13 | 906 | Ccl7/Ccl5/Irf7/Ccl8/Ccr5/Zbp1/Ifit3/Mx1/Il1b/Ifit2/Zfp36/Oas3/Socs3/Gbp7/Egr1/Traf1/Gbp5/Eps8 | 18/32 | 906/21092 |
| **GO:0006955** | GO:BP | GO:0006955 | immune response | 1.67e-13 | 1913 | Ccl7/Ccl5/Irf7/Ccl8/Ccr5/Fgl2/Zbp1/Ifit3/Mx1/ Il1b/Ifit2/Tap1/Oas3/Slamf7/Ptger4/Nfkbiz/Bcl3/ Ifi44/Lcn2/Gbp7/Isg20/Gbp5 | 22/31 | 1913/21092 |
| **GO:0002376** | GO:BP | GO:0002376 | immune system process | 3.61e-13 | 2842 | Ccl7/Ccl5/Irf7/Ccl8/Ccr5/Fgl2/Zbp1/Ifit3/Mx1/ Il1b/Ifit2/Zfp36/Tap1/Oas3/Slamf7/Ptger4/Nfkbiz/Bcl3/Ifi44/Lcn2/Gbp7/Egr1/Isg20/Gbp5/Eps8 | 25/32 | 2842/21092 |
| **GO:0009605** | GO:BP | GO:0009605 | response to external stimulus | 2.38e-11 | 2814 | Ccl7/Ccl5/Irf7/Ccl8/Ccr5/Fgl2/Zbp1/Ifit3/Mx1/ Il1b/Ifit2/Zfp36/Oas3/Slamf7/Ptger4/Nfkbiz/Bcl3/Ifi44/Lcn2/Socs3/Gbp7 | 21/26 | 2814/21092 |
| **GO:0098542** | GO:BP | GO:0098542 | defense response to other organism | 8.16e-11 | 1163 | Ccl7/Ccl5/Irf7/Ccl8/Fgl2/Zbp1/Ifit3/Mx1/Il1b/Ifit2/Oas3/Slamf7/Bcl3/Lcn2/Gbp7/Isg20/Gbp5 | 17/31 | 1163/21092 |
| **GO:0006950** | GO:BP | GO:0006950 | response to stress | 2.28e-10 | 3938 | Ccl7/Ccl5/Irf7/Ccl8/Ccr5/Fgl2/Zbp1/Ifit3/Mx1/ Il1b/Ifit2/Zfp36/Tap1/Oas3/Slamf7/Ptger4/Nfkbiz/Bcl3/Lcn2/Socs3/Gbp7/Egr1/Isg20/Traf1/Gbp5 | 25/31 | 3938/21092 |
| **GO:0051607** | GO:BP | GO:0051607 | defense response to virus | 9.68e-09 | 305 | Irf7/Fgl2/Zbp1/Ifit3/Mx1/Il1b/Ifit2/Oas3/Gbp7/ Isg20 | 10/29 | 305/21092 |
| **GO:0140546** | GO:BP | GO:0140546 | defense response to symbiont | 9.99e-09 | 306 | Irf7/Fgl2/Zbp1/Ifit3/Mx1/Il1b/Ifit2/Oas3/Gbp7/ Isg20 | 10/29 | 306/21092 |
| **GO:0045087** | GO:BP | GO:0045087 | innate immune response | 1.82e-08 | 916 | Ccl7/Ccl5/Irf7/Ccl8/Zbp1/Ifit3/Mx1/Ifit2/Oas3/ Slamf7/Lcn2/Gbp7/Isg20/Gbp5 | 14/31 | 916/21092 |
| **GO:0019221** | GO:BP | GO:0019221 | cytokine-mediated signaling pathway | 4.88e-08 | 479 | Ccl7/Ccl5/Irf7/Ccl8/Ccr5/Zbp1/Mx1/Il1b/Oas3/ Egr1/Traf1 | 11/30 | 479/21092 |
| **GO:0071310** | GO:BP | GO:0071310 | cellular response to organic substance | 1.60e-07 | 2405 | Ccl7/Ccl5/Irf7/Ccl8/Ccr5/Zbp1/Ifit3/Mx1/Il1b/Ifit2/Zfp36/Oas3/Ptger4/Socs3/Gbp7/Egr1/Traf1/ Gbp5/Eps8 | 19/32 | 2405/21092 |
| **GO:0016032** | GO:BP | GO:0016032 | viral process | 1.90e-07 | 413 | Ccl5/Irf7/Ccl8/Ccr5/Ly6e/Mx1/Zfp36/Oas3/Gbp7/Isg20 | 10/29 | 413/21092 |
| **GO:0070887** | GO:BP | GO:0070887 | cellular response to chemical stimulus | 1.04e-06 | 3049 | Ccl7/Ccl5/Irf7/Ccl8/Ccr5/Slco2b1/Zbp1/Ifit3/Mx1/Il1b/Ifit2/Zfp36/Oas3/Ptger4/Socs3/Gbp7/Egr1/Traf1/Gbp5/Eps8 | 20/32 | 3049/21092 |
| **GO:0031347** | GO:BP | GO:0031347 | regulation of defense response | 1.14e-06 | 620 | Ccl5/Irf7/Fgl2/Zbp1/Il1b/Zfp36/Oas3/Ptger4/ Nfkbiz/Socs3/Gbp5 | 11/31 | 620/21092 |
| **GO:0070098** | GO:BP | GO:0070098 | chemokine-mediated signaling pathway | 2.63e-06 | 89 | Ccl7/Ccl5/Ccl8/Ccr5 | 4/5 | 89/21092 |
| **GO:0050792** | GO:BP | GO:0050792 | regulation of viral process | 3.20e-06 | 161 | Ccl5/Ly6e/Mx1/Zfp36/Oas3/Gbp7/Isg20 | 7/29 | 161/21092 |
| **GO:1990868** | GO:BP | GO:1990868 | response to chemokine | 3.89e-06 | 98 | Ccl7/Ccl5/Ccl8/Ccr5 | 4/5 | 98/21092 |
| **GO:1990869** | GO:BP | GO:1990869 | cellular response to chemokine | 3.89e-06 | 98 | Ccl7/Ccl5/Ccl8/Ccr5 | 4/5 | 98/21092 |
| **GO:0048245** | GO:BP | GO:0048245 | eosinophil chemotaxis | 4.42e-06 | 19 | Ccl7/Ccl5/Ccl8 | 3/4 | 19/21092 |
| **GO:0048525** | GO:BP | GO:0048525 | negative regulation of viral process | 4.87e-06 | 93 | Ccl5/Ly6e/Mx1/Zfp36/Oas3/Isg20 | 6/29 | 93/21092 |
| **GO:0032101** | GO:BP | GO:0032101 | regulation of response to external stimulus | 6.36e-06 | 948 | Ccl7/Ccl5/Irf7/Fgl2/Zbp1/Il1b/Zfp36/Oas3/Ptger4/Nfkbiz/Socs3 | 11/25 | 948/21092 |
| **GO:0071347** | GO:BP | GO:0071347 | cellular response to interleukin-1 | 6.48e-06 | 106 | Ccl7/Ccl5/Ccl8/Zbp1/Il1b | 5/13 | 106/21092 |
| **GO:0072677** | GO:BP | GO:0072677 | eosinophil migration | 8.08e-06 | 23 | Ccl7/Ccl5/Ccl8 | 3/4 | 23/21092 |
| **GO:0010033** | GO:BP | GO:0010033 | response to organic substance | 8.88e-06 | 3034 | Ccl7/Ccl5/Irf7/Ccl8/Ccr5/Zbp1/Ifit3/Mx1/Il1b/Ifit2/Zfp36/Oas3/Ptger4/Socs3/Gbp7/Egr1/Traf1/ Gbp5/Eps8 | 19/32 | 3034/21092 |
| **GO:0009617** | GO:BP | GO:0009617 | response to bacterium | 9.67e-06 | 760 | Ccl5/Ccr5/Il1b/Zfp36/Oas3/Ptger4/Bcl3/Ifi44/ Lcn2/Gbp7/Gbp5 | 11/31 | 760/21092 |
| **GO:0019058** | GO:BP | GO:0019058 | viral life cycle | 1.22e-05 | 312 | Ccl5/Ccl8/Ccr5/Ly6e/Mx1/Oas3/Gbp7/Isg20 | 8/29 | 312/21092 |
| **GO:0006954** | GO:BP | GO:0006954 | inflammatory response | 1.37e-05 | 786 | Ccl7/Ccl5/Ccl8/Ccr5/Zbp1/Il1b/Zfp36/Ptger4/ Nfkbiz/Socs3/Gbp5 | 11/31 | 786/21092 |
| **GO:0070555** | GO:BP | GO:0070555 | response to interleukin-1 | 2.03e-05 | 133 | Ccl7/Ccl5/Ccl8/Zbp1/Il1b | 5/13 | 133/21092 |
| **GO:0019079** | GO:BP | GO:0019079 | viral genome replication | 2.77e-05 | 124 | Ccl5/Ccl8/Mx1/Oas3/Gbp7/Isg20 | 6/29 | 124/21092 |
| **GO:0050896** | GO:BP | GO:0050896 | response to stimulus | 3.30e-05 | 9000 | Ccl7/Ccl5/Irf7/Ccl8/Ccr5/Slco2b1/Fgl2/Ly6e/Zbp1/Ifit3/Mx1/Il1b/Ifit2/Zfp36/Tap1/Oas3/Slamf7/ Ptger4/Nfkbiz/Bcl3/Ifi44/Lcn2/Socs3/Gbp7/Egr1/ Isg20/Traf1/Gbp5/Eps8 | 29/32 | 9000/21092 |
| **GO:1903900** | GO:BP | GO:1903900 | regulation of viral life cycle | 5.50e-05 | 139 | Ccl5/Ly6e/Mx1/Oas3/Gbp7/Isg20 | 6/29 | 139/21092 |
| **GO:0002682** | GO:BP | GO:0002682 | regulation of immune system process | 6.75e-05 | 1488 | Ccl7/Ccl5/Irf7/Ccl8/Fgl2/Zbp1/Il1b/Zfp36/Oas3/ Ptger4/Nfkbiz | 11/21 | 1488/21092 |
| **GO:0071674** | GO:BP | GO:0071674 | mononuclear cell migration | 7.67e-05 | 205 | Ccl7/Ccl5/Ccl8/Ccr5 | 4/5 | 205/21092 |
| **GO:0001817** | GO:BP | GO:0001817 | regulation of cytokine production | 9.87e-05 | 733 | Irf7/Il1b/Zfp36/Oas3/Ptger4/Bcl3/Gbp7/Egr1/ Nav3/Gbp5 | 10/31 | 733/21092 |
| **GO:0001816** | GO:BP | GO:0001816 | cytokine production | 0.0001 | 739 | Irf7/Il1b/Zfp36/Oas3/Ptger4/Bcl3/Gbp7/Egr1/ Nav3/Gbp5 | 10/31 | 739/21092 |
| **GO:2000503** | GO:BP | GO:2000503 | positive regulation of natural killer cell chemotaxis | 0.0001 | 6 | Ccl7/Ccl5 | 2/2 | 6/21092 |
| **GO:0006915** | GO:BP | GO:0006915 | apoptotic process | 0.0001 | 1889 | Ccl5/Irf7/Zbp1/Atf5/Ifit3/Mx1/Il1b/Ifit2/Zfp36/ Bcl3/Lcn2/Socs3/Egr1/Traf1 | 14/30 | 1889/21092 |
| **GO:0030595** | GO:BP | GO:0030595 | leukocyte chemotaxis | 0.0001 | 239 | Ccl7/Ccl5/Ccl8/Ccr5 | 4/5 | 239/21092 |
| **GO:0012501** | GO:BP | GO:0012501 | programmed cell death | 0.0001 | 1942 | Ccl5/Irf7/Zbp1/Atf5/Ifit3/Mx1/Il1b/Ifit2/Zfp36/ Bcl3/Lcn2/Socs3/Egr1/Traf1 | 14/30 | 1942/21092 |
| **GO:0048247** | GO:BP | GO:0048247 | lymphocyte chemotaxis | 0.0001 | 64 | Ccl7/Ccl5/Ccl8 | 3/4 | 64/21092 |
| **GO:0045069** | GO:BP | GO:0045069 | regulation of viral genome replication | 0.0001 | 86 | Ccl5/Mx1/Oas3/Gbp7/Isg20 | 5/29 | 86/21092 |
| **GO:2000501** | GO:BP | GO:2000501 | regulation of natural killer cell chemotaxis | 0.0002 | 8 | Ccl7/Ccl5 | 2/2 | 8/21092 |
| **GO:0002548** | GO:BP | GO:0002548 | monocyte chemotaxis | 0.0002 | 69 | Ccl7/Ccl5/Ccl8 | 3/4 | 69/21092 |
| **GO:0050727** | GO:BP | GO:0050727 | regulation of inflammatory response | 0.0002 | 360 | Ccl5/Zbp1/Il1b/Zfp36/Ptger4/Nfkbiz/Socs3 | 7/25 | 360/21092 |
| **GO:0032103** | GO:BP | GO:0032103 | positive regulation of response to external stimulus | 0.0003 | 449 | Ccl7/Ccl5/Irf7/Zbp1/Il1b/Ptger4/Nfkbiz | 7/21 | 449/21092 |
| **GO:0006874** | GO:BP | GO:0006874 | cellular calcium ion homeostasis | 0.0003 | 290 | Ccl7/Ccl5/Ccl8/Ccr5 | 4/5 | 290/21092 |
| **GO:0031349** | GO:BP | GO:0031349 | positive regulation of defense response | 0.0003 | 293 | Ccl5/Irf7/Zbp1/Il1b/Ptger4/Nfkbiz/Gbp5 | 7/31 | 293/21092 |
| **GO:0035747** | GO:BP | GO:0035747 | natural killer cell chemotaxis | 0.0003 | 10 | Ccl7/Ccl5 | 2/2 | 10/21092 |
| **GO:0080134** | GO:BP | GO:0080134 | regulation of response to stress | 0.0003 | 1356 | Ccl5/Irf7/Fgl2/Zbp1/Il1b/Zfp36/Oas3/Ptger4/ Nfkbiz/Socs3/Traf1/Gbp5 | 12/31 | 1356/21092 |
| **GO:0007166** | GO:BP | GO:0007166 | cell surface receptor signaling pathway | 0.0003 | 2816 | Ccl7/Ccl5/Irf7/Ccl8/Ccr5/Ly6e/Zbp1/Mx1/Il1b/ Oas3/Nfkbiz/Bcl3/Socs3/Gbp7/Egr1/Traf1 | 16/30 | 2816/21092 |
| **GO:0055074** | GO:BP | GO:0055074 | calcium ion homeostasis | 0.0004 | 313 | Ccl7/Ccl5/Ccl8/Ccr5 | 4/5 | 313/21092 |
| **GO:0060326** | GO:BP | GO:0060326 | cell chemotaxis | 0.0004 | 314 | Ccl7/Ccl5/Ccl8/Ccr5 | 4/5 | 314/21092 |
| **GO:0042110** | GO:BP | GO:0042110 | T cell activation | 0.0004 | 538 | Ccl5/Fgl2/Il1b/Slamf7/Ptger4/Nfkbiz/Bcl3/Egr1 | 8/27 | 538/21092 |
| **GO:0072503** | GO:BP | GO:0072503 | cellular divalent inorganic cation homeostasis | 0.0005 | 327 | Ccl7/Ccl5/Ccl8/Ccr5 | 4/5 | 327/21092 |
| **GO:0008219** | GO:BP | GO:0008219 | cell death | 0.0005 | 2107 | Ccl5/Irf7/Zbp1/Atf5/Ifit3/Mx1/Il1b/Ifit2/Zfp36/ Bcl3/Lcn2/Socs3/Egr1/Traf1 | 14/30 | 2107/21092 |
| **GO:0042981** | GO:BP | GO:0042981 | regulation of apoptotic process | 0.0005 | 1453 | Ccl5/Irf7/Zbp1/Atf5/Ifit3/Il1b/Ifit2/Zfp36/Bcl3/Socs3/Egr1/Traf1 | 12/30 | 1453/21092 |
| **GO:0002831** | GO:BP | GO:0002831 | regulation of response to biotic stimulus | 0.0005 | 351 | Ccl5/Irf7/Fgl2/Zbp1/Il1b/Oas3 | 6/18 | 351/21092 |
| **GO:0097530** | GO:BP | GO:0097530 | granulocyte migration | 0.0005 | 160 | Ccl7/Ccl5/Ccl8/Il1b/Ptger4 | 5/20 | 160/21092 |
| **GO:0042221** | GO:BP | GO:0042221 | response to chemical | 0.0006 | 4383 | Ccl7/Ccl5/Irf7/Ccl8/Ccr5/Slco2b1/Zbp1/Ifit3/Mx1/Il1b/Ifit2/Zfp36/Oas3/Ptger4/Socs3/Gbp7/Egr1/Traf1/Gbp5/Eps8 | 20/32 | 4383/21092 |
| **GO:0043067** | GO:BP | GO:0043067 | regulation of programmed cell death | 0.0006 | 1482 | Ccl5/Irf7/Zbp1/Atf5/Ifit3/Il1b/Ifit2/Zfp36/Bcl3/ Socs3/Egr1/Traf1 | 12/30 | 1482/21092 |
| **GO:0072507** | GO:BP | GO:0072507 | divalent inorganic cation homeostasis | 0.0007 | 358 | Ccl7/Ccl5/Ccl8/Ccr5 | 4/5 | 358/21092 |
| **GO:0030593** | GO:BP | GO:0030593 | neutrophil chemotaxis | 0.0007 | 106 | Ccl7/Ccl5/Ccl8/Il1b | 4/13 | 106/21092 |
| **GO:0048583** | GO:BP | GO:0048583 | regulation of response to stimulus | 0.0007 | 3970 | Ccl7/Ccl5/Irf7/Ccl8/Fgl2/Ly6e/Zbp1/Il1b/Zfp36/ Oas3/Ptger4/Nfkbiz/Bcl3/Socs3/Gbp7/Egr1/Traf1/Gbp5/Eps8 | 19/32 | 3970/21092 |
| **GO:0050900** | GO:BP | GO:0050900 | leukocyte migration | 0.0010 | 397 | Ccl7/Ccl5/Ccl8/Ccr5 | 4/5 | 397/21092 |
| **GO:0034340** | GO:BP | GO:0034340 | response to type I interferon | 0.0011 | 83 | Irf7/Zbp1/Mx1/Oas3 | 4/18 | 83/21092 |
| **GO:0071346** | GO:BP | GO:0071346 | cellular response to interferon-gamma | 0.0013 | 121 | Ccl7/Ccl5/Ccl8 | 3/4 | 121/21092 |
| **GO:0072676** | GO:BP | GO:0072676 | lymphocyte migration | 0.0013 | 123 | Ccl7/Ccl5/Ccl8 | 3/4 | 123/21092 |
| **GO:0006875** | GO:BP | GO:0006875 | cellular metal ion homeostasis | 0.0013 | 422 | Ccl7/Ccl5/Ccl8/Ccr5 | 4/5 | 422/21092 |
| **GO:1990266** | GO:BP | GO:1990266 | neutrophil migration | 0.00166 | 131 | Ccl7/Ccl5/Ccl8 | 3/4 | 131/21092 |
| **GO:0071621** | GO:BP | GO:0071621 | granulocyte chemotaxis | 0.00166 | 131 | Ccl7/Ccl5/Ccl8 | 3/4 | 131/21092 |
| **GO:0045071** | GO:BP | GO:0045071 | negative regulation of viral genome replication | 0.0017 | 56 | Ccl5/Mx1/Oas3/Isg20 | 4/29 | 56/21092 |
| **GO:0140131** | GO:BP | GO:0140131 | positive regulation of lymphocyte chemotaxis | 0.0018 | 22 | Ccl7/Ccl5 | 2/2 | 22/21092 |
| **GO:0010941** | GO:BP | GO:0010941 | regulation of cell death | 0.0018 | 1637 | Ccl5/Irf7/Zbp1/Atf5/Ifit3/Il1b/Ifit2/Zfp36/Bcl3/ Socs3/Egr1/Traf1 | 12/30 | 1637/21092 |
| **GO:0034341** | GO:BP | GO:0034341 | response to interferon-gamma | 0.0021 | 142 | Ccl7/Ccl5/Ccl8 | 3/4 | 142/21092 |
| **GO:0002292** | GO:BP | GO:0002292 | T cell differentiation involved in immune response | 0.0021 | 78 | Fgl2/Ptger4/Nfkbiz/Bcl3 | 4/22 | 78/21092 |
| **GO:0002687** | GO:BP | GO:0002687 | positive regulation of leukocyte migration | 0.0025 | 151 | Ccl7/Ccl5/Ccl8 | 3/4 | 151/21092 |
| **GO:0030003** | GO:BP | GO:0030003 | cellular cation homeostasis | 0.0027 | 499 | Ccl7/Ccl5/Ccl8/Ccr5 | 4/5 | 499/21092 |
| **GO:0001818** | GO:BP | GO:0001818 | negative regulation of cytokine production | 0.0027 | 281 | Zfp36/Oas3/Ptger4/Bcl3/Gbp7/Nav3 | 6/28 | 281/21092 |
| **GO:1903131** | GO:BP | GO:1903131 | mononuclear cell differentiation | 0.0028 | 470 | Irf7/Fgl2/Il1b/Ptger4/Nfkbiz/Bcl3/Egr1 | 7/27 | 470/21092 |
| **GO:1901623** | GO:BP | GO:1901623 | regulation of lymphocyte chemotaxis | 0.0028 | 27 | Ccl7/Ccl5 | 2/2 | 27/21092 |
| **GO:0030217** | GO:BP | GO:0030217 | T cell differentiation | 0.0030 | 297 | Fgl2/Il1b/Ptger4/Nfkbiz/Bcl3/Egr1 | 6/27 | 297/21092 |
| **GO:0006873** | GO:BP | GO:0006873 | cellular ion homeostasis | 0.0031 | 516 | Ccl7/Ccl5/Ccl8/Ccr5 | 4/5 | 516/21092 |
| **GO:0055065** | GO:BP | GO:0055065 | metal ion homeostasis | 0.0031 | 520 | Ccl7/Ccl5/Ccl8/Ccr5 | 4/5 | 520/21092 |
| **GO:0097529** | GO:BP | GO:0097529 | myeloid leukocyte migration | 0.0044 | 240 | Ccl7/Ccl5/Ccl8/Il1b/Ptger4 | 5/20 | 240/21092 |
| **GO:0046426** | GO:BP | GO:0046426 | negative regulation of receptor signaling pathway via JAK-STAT | 0.0045 | 22 | Bcl3/Socs3/Gbp7 | 3/26 | 22/21092 |
| **GO:0044403** | GO:BP | GO:0044403 | biological process involved in symbiotic interaction | 0.0048 | 301 | Ccl5/Ccl8/Ccr5/Ly6e | 4/8 | 301/21092 |
| **GO:0055080** | GO:BP | GO:0055080 | cation homeostasis | 0.0056 | 601 | Ccl7/Ccl5/Ccl8/Ccr5 | 4/5 | 601/21092 |
| **GO:0098771** | GO:BP | GO:0098771 | inorganic ion homeostasis | 0.0062 | 616 | Ccl7/Ccl5/Ccl8/Ccr5 | 4/5 | 616/21092 |
| **GO:0006935** | GO:BP | GO:0006935 | chemotaxis | 0.0064 | 620 | Ccl7/Ccl5/Ccl8/Ccr5 | 4/5 | 620/21092 |
| **GO:0042330** | GO:BP | GO:0042330 | taxis | 0.0065 | 622 | Ccl7/Ccl5/Ccl8/Ccr5 | 4/5 | 622/21092 |
| **GO:0050801** | GO:BP | GO:0050801 | ion homeostasis | 0.0067 | 626 | Ccl7/Ccl5/Ccl8/Ccr5 | 4/5 | 626/21092 |
| **GO:1904893** | GO:BP | GO:1904893 | negative regulation of receptor signaling pathway via STAT | 0.0067 | 25 | Bcl3/Socs3/Gbp7 | 3/26 | 25/21092 |
| **GO:0002252** | GO:BP | GO:0002252 | immune effector process | 0.0070 | 680 | Irf7/Fgl2/Il1b/Slamf7/Ptger4/Nfkbiz/Bcl3 | 7/22 | 680/21092 |
| **GO:0070374** | GO:BP | GO:0070374 | positive regulation of ERK1 and ERK2 cascade | 0.0071 | 213 | Ccl7/Ccl5/Ccl8 | 3/4 | 213/21092 |
| **GO:2000403** | GO:BP | GO:2000403 | positive regulation of lymphocyte migration | 0.0072 | 43 | Ccl7/Ccl5 | 2/2 | 43/21092 |
| **GO:0002684** | GO:BP | GO:0002684 | positive regulation of immune system process | 0.0074 | 954 | Ccl7/Ccl5/Irf7/Ccl8 | 4/4 | 954/21092 |
| **GO:0000165** | GO:BP | GO:0000165 | MAPK cascade | 0.0076 | 769 | Ccl7/Ccl5/Ccl8/Ccr5/Il1b/Zfp36/Ptger4 | 7/20 | 769/21092 |
| **GO:0071356** | GO:BP | GO:0071356 | cellular response to tumor necrosis factor | 0.0081 | 222 | Ccl7/Ccl5/Ccl8 | 3/4 | 222/21092 |
| **GO:0001819** | GO:BP | GO:0001819 | positive regulation of cytokine production | 0.0084 | 477 | Irf7/Il1b/Oas3/Ptger4/Bcl3/Egr1/Gbp5 | 7/31 | 477/21092 |
| **GO:0055082** | GO:BP | GO:0055082 | cellular chemical homeostasis | 0.0089 | 673 | Ccl7/Ccl5/Ccl8/Ccr5 | 4/5 | 673/21092 |
| **GO:0060338** | GO:BP | GO:0060338 | regulation of type I interferon-mediated signaling pathway | 0.0090 | 40 | Irf7/Zbp1/Oas3 | 3/18 | 40/21092 |
| **GO:0002685** | GO:BP | GO:0002685 | regulation of leukocyte migration | 0.0090 | 230 | Ccl7/Ccl5/Ccl8 | 3/4 | 230/21092 |
| **GO:0070498** | GO:BP | GO:0070498 | interleukin-1-mediated signaling pathway | 0.0095 | 27 | Zbp1/Il1b/Egr1 | 3/27 | 27/21092 |
| **GO:0002286** | GO:BP | GO:0002286 | T cell activation involved in immune response | 0.0105 | 116 | Fgl2/Ptger4/Nfkbiz/Bcl3 | 4/22 | 116/21092 |
| **GO:0034612** | GO:BP | GO:0034612 | response to tumor necrosis factor | 0.0105 | 242 | Ccl7/Ccl5/Ccl8 | 3/4 | 242/21092 |
| **GO:0031348** | GO:BP | GO:0031348 | negative regulation of defense response | 0.0117 | 230 | Fgl2/Zfp36/Oas3/Ptger4/Socs3 | 5/25 | 230/21092 |
| **GO:0046649** | GO:BP | GO:0046649 | lymphocyte activation | 0.0117 | 834 | Ccl5/Fgl2/Il1b/Slamf7/Ptger4/Nfkbiz/Bcl3/Egr1 | 8/27 | 834/21092 |
| **GO:0046425** | GO:BP | GO:0046425 | regulation of receptor signaling pathway via JAK-STAT | 0.0121 | 101 | Ccl5/Bcl3/Socs3/Gbp7 | 4/26 | 101/21092 |
| **GO:0001959** | GO:BP | GO:0001959 | regulation of cytokine-mediated signaling pathway | 0.0127 | 151 | Ccl5/Irf7/Zbp1/Oas3 | 4/18 | 151/21092 |
| **GO:0002521** | GO:BP | GO:0002521 | leukocyte differentiation | 0.0133 | 595 | Irf7/Fgl2/Il1b/Ptger4/Nfkbiz/Bcl3/Egr1 | 7/27 | 595/21092 |
| **GO:1904892** | GO:BP | GO:1904892 | regulation of receptor signaling pathway via STAT | 0.0153 | 107 | Ccl5/Bcl3/Socs3/Gbp7 | 4/26 | 107/21092 |
| **GO:0019725** | GO:BP | GO:0019725 | cellular homeostasis | 0.0154 | 772 | Ccl7/Ccl5/Ccl8/Ccr5 | 4/5 | 772/21092 |
| **GO:0043547** | GO:BP | GO:0043547 | positive regulation of GTPase activity | 0.0156 | 276 | Ccl7/Ccl5/Ccl8 | 3/4 | 276/21092 |
| **GO:0060759** | GO:BP | GO:0060759 | regulation of response to cytokine stimulus | 0.0168 | 162 | Ccl5/Irf7/Zbp1/Oas3 | 4/18 | 162/21092 |
| **GO:0070371** | GO:BP | GO:0070371 | ERK1 and ERK2 cascade | 0.0187 | 323 | Ccl7/Ccl5/Ccl8/Il1b/Ptger4 | 5/20 | 323/21092 |
| **GO:2000401** | GO:BP | GO:2000401 | regulation of lymphocyte migration | 0.0188 | 69 | Ccl7/Ccl5 | 2/2 | 69/21092 |
| **GO:0035556** | GO:BP | GO:0035556 | intracellular signal transduction | 0.0195 | 2627 | Ccl7/Ccl5/Irf7/Ccl8/Ccr5/Il1b/Zfp36/Oas3/Ptger4/Bcl3/Socs3/Gbp7/Traf1/Eps8 | 14/32 | 2627/21092 |
| **GO:0032496** | GO:BP | GO:0032496 | response to lipopolysaccharide | 0.0198 | 327 | Ccl5/Ccr5/Il1b/Zfp36/Ptger4 | 5/20 | 327/21092 |
| **GO:0070372** | GO:BP | GO:0070372 | regulation of ERK1 and ERK2 cascade | 0.0199 | 299 | Ccl7/Ccl5/Ccl8 | 3/4 | 299/21092 |
| **GO:0050729** | GO:BP | GO:0050729 | positive regulation of inflammatory response | 0.0209 | 145 | Zbp1/Il1b/Ptger4/Nfkbiz | 4/21 | 145/21092 |
| **GO:0071222** | GO:BP | GO:0071222 | cellular response to lipopolysaccharide | 0.0213585076515551 | 210 | Ccl5/Ccr5/Il1b/Zfp36 | 4/15 | 210/21092 |
| **GO:0071677** | GO:BP | GO:0071677 | positive regulation of mononuclear cell migration | 0.0216 | 74 | Ccl7/Ccl5 | 2/2 | 74/21092 |
| **GO:0030098** | GO:BP | GO:0030098 | lymphocyte differentiation | 0.0226 | 421 | Fgl2/Il1b/Ptger4/Nfkbiz/Bcl3/Egr1 | 6/27 | 421/21092 |
| **GO:0030097** | GO:BP | GO:0030097 | hemopoiesis | 0.0246 | 923 | Irf7/Fgl2/Il1b/Zfp36/Ptger4/Nfkbiz/Bcl3/Egr1 | 8/27 | 923/21092 |
| **GO:0071219** | GO:BP | GO:0071219 | cellular response to molecule of bacterial origin | 0.0251 | 219 | Ccl5/Ccr5/Il1b/Zfp36 | 4/15 | 219/21092 |
| **GO:0002237** | GO:BP | GO:0002237 | response to molecule of bacterial origin | 0.0260 | 346 | Ccl5/Ccr5/Il1b/Zfp36/Ptger4 | 5/20 | 346/21092 |
| **GO:0002407** | GO:BP | GO:0002407 | dendritic cell chemotaxis | 0.0281 | 27 | Ccl5/Ccr5 | 2/5 | 27/21092 |
| **GO:0048585** | GO:BP | GO:0048585 | negative regulation of response to stimulus | 0.0284 | 1604 | Ccl5/Fgl2/Il1b/Zfp36/Oas3/Ptger4/Bcl3/Socs3/ Gbp7/Egr1 | 10/27 | 1604/21092 |
| **GO:0050776** | GO:BP | GO:0050776 | regulation of immune response | 0.0306 | 899 | Ccl5/Irf7/Fgl2/Zbp1/Il1b/Oas3/Nfkbiz | 7/21 | 899/21092 |
| **GO:0036336** | GO:BP | GO:0036336 | dendritic cell migration | 0.0328 | 34 | Ccl5/Ccr5/Eps8 | 3/32 | 34/21092 |
| **GO:0048534** | GO:BP | GO:0048534 | hematopoietic or lymphoid organ development | 0.0337 | 964 | Irf7/Fgl2/Il1b/Zfp36/Ptger4/Nfkbiz/Bcl3/Egr1 | 8/27 | 964/21092 |
| **GO:2000107** | GO:BP | GO:2000107 | negative regulation of leukocyte apoptotic process | 0.0344 | 54 | Ccl5/Irf7 | 2/3 | 54/21092 |
| **GO:0045088** | GO:BP | GO:0045088 | regulation of innate immune response | 0.0362 | 231 | Ccl5/Irf7/Zbp1/Oas3/Gbp5 | 5/31 | 231/21092 |
| **GO:0043087** | GO:BP | GO:0043087 | regulation of GTPase activity | 0.0368 | 367 | Ccl7/Ccl5/Ccl8 | 3/4 | 367/21092 |
| **GO:0002690** | GO:BP | GO:0002690 | positive regulation of leukocyte chemotaxis | 0.0381 | 98 | Ccl7/Ccl5 | 2/2 | 98/21092 |
| **GO:0071216** | GO:BP | GO:0071216 | cellular response to biotic stimulus | 0.0397 | 246 | Ccl5/Ccr5/Il1b/Zfp36 | 4/15 | 246/21092 |
| **GO:0045321** | GO:BP | GO:0045321 | leukocyte activation | 0.0423 | 995 | Ccl5/Fgl2/Il1b/Slamf7/Ptger4/Nfkbiz/Bcl3/Egr1 | 8/27 | 995/21092 |
| **GO:0045089** | GO:BP | GO:0045089 | positive regulation of innate immune response | 0.0435 | 142 | Ccl5/Irf7/Zbp1 | 3/9 | 142/21092 |
| **GO:0035457** | GO:BP | GO:0035457 | cellular response to interferon-alpha | 0.0480 | 12 | Ifit3/Ifit2 | 2/14 | 12/21092 |
| **GO:0009967** | GO:BP | GO:0009967 | positive regulation of signal transduction | 0.0483 | 1523 | Ccl7/Ccl5/Irf7/Ccl8 | 4/4 | 1523/21092 |
| **GO:0060340** | GO:BP | GO:0060340 | positive regulation of type I interferon-mediated signaling pathway | 0.0492 | 19 | Irf7/Zbp1 | 2/9 | 19/21092 |
| **GO:0048020** | GO:MF | GO:0048020 | CCR chemokine receptor binding | 1.78e-05 | 47 | Ccl7/Ccl5/Ccl8 | 3/4 | 47/20166 |
| **GO:0008009** | GO:MF | GO:0008009 | chemokine activity | 2.15e-05 | 50 | Ccl7/Ccl5/Ccl8 | 3/4 | 50/20166 |
| **GO:0031726** | GO:MF | GO:0031726 | CCR1 chemokine receptor binding | 2.77e-05 | 6 | Ccl7/Ccl5 | 2/2 | 6/20166 |
| **GO:0042379** | GO:MF | GO:0042379 | chemokine receptor binding | 6.82e-05 | 73 | Ccl7/Ccl5/Ccl8 | 3/4 | 73/20166 |
| **GO:0016004** | GO:MF | GO:0016004 | phospholipase activator activity | 0.0010 | 14 | Ccl5/Ccl8 | 2/4 | 14/20166 |
| **GO:0060229** | GO:MF | GO:0060229 | lipase activator activity | 0.0015 | 17 | Ccl5/Ccl8 | 2/4 | 17/20166 |
| **GO:0005125** | GO:MF | GO:0005125 | cytokine activity | 0.0023 | 237 | Ccl7/Ccl5/Ccl8 | 3/4 | 237/20166 |
| **GO:0005126** | GO:MF | GO:0005126 | cytokine receptor binding | 0.0038 | 277 | Ccl7/Ccl5/Ccl8 | 3/4 | 277/20166 |
| **GO:0001664** | GO:MF | GO:0001664 | G protein-coupled receptor binding | 0.0043 | 290 | Ccl7/Ccl5/Ccl8 | 3/4 | 290/20166 |
| **GO:0030545** | GO:MF | GO:0030545 | signaling receptor regulator activity | 0.0056 | 543 | Ccl7/Ccl5/Ccl8/Ly6e/Il1b | 5/13 | 543/20166 |
| **GO:0004435** | GO:MF | GO:0004435 | phosphatidylinositol phospholipase C activity | 0.0059 | 26 | Ccl5/Ccr5 | 2/5 | 26/20166 |
| **GO:0004629** | GO:MF | GO:0004629 | phospholipase C activity | 0.0069 | 28 | Ccl5/Ccr5 | 2/5 | 28/20166 |
| **GO:0048018** | GO:MF | GO:0048018 | receptor ligand activity | 0.0223 | 500 | Ccl7/Ccl5/Ccl8 | 3/4 | 500/20166 |
| **GO:0030547** | GO:MF | GO:0030547 | signaling receptor inhibitor activity | 0.0223 | 30 | Ccl5/Ly6e | 2/8 | 30/20166 |
| **GO:0030546** | GO:MF | GO:0030546 | signaling receptor activator activity | 0.0234 | 508 | Ccl7/Ccl5/Ccl8 | 3/4 | 508/20166 |
| **GO:1904680** | GO:MF | GO:1904680 | peptide transmembrane transporter activity | 0.0299 | 16 | Tap1/Slc13a3 | 2/17 | 16/20166 |
| **GO:0046817** | GO:MF | GO:0046817 | chemokine receptor antagonist activity | 0.0372 | 1 | Ccl5 | 1/2 | 1/20166 |
| **KEGG:04061** | KEGG | KEGG:04061 | Viral protein interaction with cytokine and cytokine receptor | 6.488e-06 | 98 | Ccl7/Ccl5/Ccl8/Ccr5 | 4/5 | 98/8064 |
| **KEGG:04062** | KEGG | KEGG:04062 | Chemokine signaling pathway | 9.36e-05 | 190 | Ccl7/Ccl5/Ccl8/Ccr5 | 4/5 | 190/8064 |
| **KEGG:04623** | KEGG | KEGG:04623 | Cytosolic DNA-sensing pathway | 0.0001 | 62 | Ccl5/Irf7/Zbp1/Il1b | 4/13 | 62/8064 |
| **KEGG:04060** | KEGG | KEGG:04060 | Cytokine-cytokine receptor interaction | 0.0005 | 293 | Ccl7/Ccl5/Ccl8/Ccr5 | 4/5 | 293/8064 |
| **KEGG:05164** | KEGG | KEGG:05164 | Influenza A | 0.0006 | 168 | Ccl5/Irf7/Mx1/Il1b/Oas3/Socs3 | 6/25 | 168/8064 |
| **KEGG:04668** | KEGG | KEGG:04668 | TNF signaling pathway | 0.0032 | 112 | Ccl5/Il1b/Bcl3/Socs3/Traf1 | 5/30 | 112/8064 |
| **KEGG:04621** | KEGG | KEGG:04621 | NOD-like receptor signaling pathway | 0.0034 | 181 | Ccl5/Irf7/Il1b/Oas3/Gbp7/Gbp5 | 6/31 | 181/8064 |
| **KEGG:05163** | KEGG | KEGG:05163 | Human cytomegalovirus infection | 0.0108 | 223 | Ccl5/Ccr5/Il1b/Tap1/Ptger4 | 5/20 | 223/8064 |
| **KEGG:05162** | KEGG | KEGG:05162 | Measles | 0.0136 | 139 | Irf7/Mx1/Il1b/Oas3 | 4/18 | 139/8064 |
| **KEGG:04620** | KEGG | KEGG:04620 | Toll-like receptor signaling pathway | 0.0301 | 102 | Ccl5/Irf7 | 2/3 | 102/8064 |
| **REAC:R-HSA-909733** | REAC | REAC:R-HSA-909733 | Interferon alpha/beta signaling | 2.55e-09 | 68 | Irf7/Ifit3/Mx1/Ifit2/Oas3/Socs3/Egr1/Isg20 | 8/29 | 68/10461 |
| **REAC:R-HSA-913531** | REAC | REAC:R-HSA-913531 | Interferon Signaling | 3.46e-08 | 192 | Irf7/Ifit3/Mx1/Ifit2/Oas3/Socs3/Gbp7/Egr1/Isg20/Gbp5 | 10/31 | 192/10461 |
| **REAC:R-HSA-1280215** | REAC | REAC:R-HSA-1280215 | Cytokine Signaling in Immune system | 9.93e-07 | 704 | Ccl5/Irf7/Ccr5/Ifit3/Mx1/Il1b/Ifit2/Oas3/Lcn2/ Socs3/Gbp7/Egr1/Isg20/Gbp5 | 14/31 | 704/10461 |
| **REAC:R-HSA-380108** | REAC | REAC:R-HSA-380108 | Chemokine receptors bind chemokines | 0.0004 | 57 | Ccl7/Ccl5/Ccr5 | 3/5 | 57/10461 |
| **REAC:R-HSA-168256** | REAC | REAC:R-HSA-168256 | Immune System | 0.0007 | 2039 | Ccl5/Irf7/Ccr5/Fgl2/Zbp1/Ifit3/Mx1/Il1b/Ifit2/ Tap1/Oas3/Slamf7/Lcn2/Socs3/Gbp7/Egr1/Isg20/Gbp5 | 18/31 | 2039/10461 |
| **REAC:R-HSA-877300** | REAC | REAC:R-HSA-877300 | Interferon gamma signaling | 0.0015 | 86 | Irf7/Oas3/Socs3/Gbp7/Gbp5 | 5/31 | 86/10461 |
| **REAC:R-HSA-6783783** | REAC | REAC:R-HSA-6783783 | Interleukin-10 signaling | 0.0065 | 45 | Ccl5/Ccr5/Il1b | 3/13 | 45/10461 |
| **REAC:R-HSA-375276** | REAC | REAC:R-HSA-375276 | Peptide ligand-binding receptors | 0.0197 | 195 | Ccl7/Ccl5/Ccr5 | 3/5 | 195/10461 |
| **TF:M11677** | TF | TF:M11677 | Factor: IRF-3; motif: NGGAAACNGAAACCGAAACN | 6.77e-10 | 468 | Ccl5/Irf7/Ccl8/Zbp1/Atf5/Ifit3/Mx1/Ifit2/Zfp36/ Tap1 | 10/16 | 468/19943 |
| **TF:M09957** | TF | TF:M09957 | Factor: IRF-2; motif: NAAANNGAAAGTGAAASTRN | 9.24e-07 | 272 | Zbp1/Ifit3/Mx1/Ifit2/Zfp36/Tap1/Oas3/Ifi44 | 8/23 | 272/19943 |
| **TF:M04014** | TF | TF:M04014 | Factor: IRF3; motif: NNRRAANGGAAACCGAAACYR | 1.26e-06 | 283 | Ccl8/Atf5/Ifit3/Mx1/Ifit2/Tap1/Oas3/Ifi44 | 8/23 | 283/19943 |
| **TF:M11676** | TF | TF:M11676 | Factor: IRF-3; motif: NGGAAANGGAAASNGAAACN | 7.86e-06 | 558 | Ccl5/Irf7/Ccl8/Zbp1/Atf5/Ifit3/Ifit2/Tap1 | 8/16 | 558/19943 |
| **TF:M07216** | TF | TF:M07216 | Factor: IRF1; motif: NNNYASTTTCACTTTCNNTTT | 3.88e-05 | 438 | Zbp1/Ifit3/Mx1/Ifit2/Zfp36/Tap1/Oas3/Ifi44 | 8/23 | 438/19943 |
| **TF:M04018** | TF | TF:M04018 | Factor: IRF7; motif: NCGAAARYGAAANT | 6.37e-05 | 628 | Irf7/Fgl2/Zbp1/Ifit3/Mx1/Ifit2/Tap1/Oas3 | 8/18 | 628/19943 |
| **TF:M11665_1** | TF | TF:M11665_1 | Factor: IRF-2; motif: NGAAASYGAAAS; match class: 1 | 0.0001 | 251 | Atf5/Ifit3/Ifit2/Zfp36/Tap1/Oas3 | 6/18 | 251/19943 |
| **TF:M04020_1** | TF | TF:M04020_1 | Factor: IRF8; motif: NCGAAACCGAAACT; match class: 1 | 0.0001 | 44 | Irf7/Zbp1/Ifit3/Oas3 | 4/18 | 44/19943 |
| **TF:M10080** | TF | TF:M10080 | Factor: STAT2; motif: RRGRAANNGAAACTGAAAN | 0.0001 | 347 | Irf7/Ccl8/Zbp1/Ifit3/Mx1/Oas3/Ifi44 | 7/23 | 347/19943 |
| **TF:M11687_1** | TF | TF:M11687_1 | Factor: IRF-4; motif: NCGAAACCGAAACYN; match class: 1 | 0.0002 | 480 | Irf7/Zbp1/Ifit3/Ifit2/Zfp36/Tap1/Oas3 | 7/18 | 480/19943 |
| **TF:M11664_1** | TF | TF:M11664_1 | Factor: IRF-2; motif: NGAAASYGAAAS; match class: 1 | 0.0002 | 143 | Atf5/Ifit3/Ifit2/Tap1/Oas3 | 5/18 | 143/19943 |
| **TF:M00258** | TF | TF:M00258 | Factor: ISGF-3; motif: CAGTTTCWCTTTYCC | 0.0003 | 838 | Irf7/Zbp1/Ifit3/Mx1/Il1b/Ifit2/Zfp36/Nfkbiz/Ifi44 | 9/23 | 838/19943 |
| **TF:M08775_1** | TF | TF:M08775_1 | Factor: IRF-2; motif: NAANYGAAASYR; match class: 1 | 0.0004 | 2756 | Ccl5/Irf7/Fgl2/Zbp1/Atf5/Ifit3/Mx1/Ifit2/Zfp36/ Tap1/Slc13a3/Oas3/Nfkbiz/Ifi44 | 14/23 | 2756/19943 |
| **TF:M11683_1** | TF | TF:M11683_1 | Factor: IRF-8; motif: NCGAAACCGAAACYN; match class: 1 | 0.0004 | 820 | Irf7/Zbp1/Atf5/Ifit3/Ifit2/Zfp36/Tap1/Oas3 | 8/18 | 820/19943 |
| **TF:M11684_1** | TF | TF:M11684_1 | Factor: IRF-8; motif: NYGAAACYGAAACTN; match class: 1 | 0.0005 | 837 | Ccl8/Zbp1/Atf5/Ifit3/Ifit2/Zfp36/Tap1/Oas3 | 8/18 | 837/19943 |
| **TF:M00699** | TF | TF:M00699 | Factor: ICSBP; motif: RAARTGAAACTG | 0.0007 | 1203 | Ccl8/Zbp1/Ifit3/Mx1/Il1b/Ifit2/Zfp36/Oas3/Nfkbiz/Ifi44 | 10/23 | 1203/19943 |
| **TF:M00453_1** | TF | TF:M00453_1 | Factor: IRF-7; motif: TNSGAAWNCGAAANTNNN; match class: 1 | 0.0007 | 336 | Ccl8/Zbp1/Ifit3/Ifit2/Tap1/Oas3 | 6/18 | 336/19943 |
| **TF:M01279_1** | TF | TF:M01279_1 | Factor: IRF-3; motif: NBNBTTTCSCTTT; match class: 1 | 0.0019 | 97 | Ccl7/Irf7/Ifit2/Tap1 | 4/16 | 97/19943 |
| **TF:M09956** | TF | TF:M09956 | Factor: IRF-1; motif: NAAANNGAAASTGAAASNRN | 0.0025 | 317 | Zbp1/Ifit3/Ifit2/Zfp36/Oas3/Ifi44 | 6/23 | 317/19943 |
| **TF:M09629** | TF | TF:M09629 | Factor: IRF-1; motif: NAAANNGAAAGTGAAASTRN | 0.0036 | 336 | Zbp1/Ifit3/Ifit2/Tap1/Oas3/Ifi44 | 6/23 | 336/19943 |
| **TF:M11686_1** | TF | TF:M11686_1 | Factor: IRF-4; motif: NYGAAASYGAAACYN; match class: 1 | 0.0042 | 456 | Atf5/Ifit3/Ifit2/Zfp36/Tap1/Oas3 | 6/18 | 456/19943 |
| **TF:M11670_1** | TF | TF:M11670_1 | Factor: IRF-5; motif: NYGAAACCGAAACY; match class: 1 | 0.0062 | 32 | Irf7/Ifit3/Oas3 | 3/18 | 32/19943 |
| **TF:M09958** | TF | TF:M09958 | Factor: IRF-3; motif: RAAARGGAAANNGAAASNGA | 0.0070 | 598 | Atf5/Ifit3/Mx1/Ifit2/Zfp36/Tap1/Ifi44 | 7/23 | 598/19943 |
| **TF:M11685_1** | TF | TF:M11685_1 | Factor: IRF-8; motif: NCGAAACYGAAACYN; match class: 1 | 0.0074 | 120 | Irf7/Ifit3/Tap1/Oas3 | 4/18 | 120/19943 |
| **TF:M11685** | TF | TF:M11685 | Factor: IRF-8; motif: NCGAAACYGAAACYN | 0.0097 | 1656 | Irf7/Zbp1/Ifit3/Mx1/Ifit2/Zfp36/Tap1/Oas3/ Nfkbiz/Ifi44/Egr1 | 11/27 | 1656/19943 |
| **TF:M11682_1** | TF | TF:M11682_1 | Factor: IRF-8; motif: NYGAAASYGAAACYN; match class: 1 | 0.0128 | 553 | Atf5/Ifit3/Ifit2/Zfp36/Tap1/Oas3 | 6/18 | 553/19943 |
| **TF:M07323** | TF | TF:M07323 | Factor: IRF-4; motif: KRAAMNGAAANYN | 0.0149 | 960 | Fgl2/Zbp1/Ifit3/Ifit2/Zfp36/Oas3/Nfkbiz/Ifi44 | 8/23 | 960/19943 |
| **TF:M00453** | TF | TF:M00453 | Factor: IRF-7; motif: TNSGAAWNCGAAANTNNN | 0.0162 | 3115 | Irf7/Ccl8/Zbp1/Atf5/Ifit3/Mx1/Ifit2/Zfp36/Tap1/ Oas3/Ptger4/Nfkbiz/Ifi44 | 13/23 | 3115/19943 |
| **TF:M04020** | TF | TF:M04020 | Factor: IRF8; motif: NCGAAACCGAAACT | 0.0180 | 1013 | Irf7/Zbp1/Ifit3/Mx1/Ifit2/Tap1/Oas3/Ifi44/Isg20 | 9/29 | 1013/19943 |
| **TF:M00772_1** | TF | TF:M00772_1 | Factor: IRF; motif: RRAANTGAAASYGNV; match class: 1 | 0.0182 | 59 | Ifit3/Mx1/Ifit2 | 3/14 | 59/19943 |
| **TF:M00772** | TF | TF:M00772 | Factor: IRF; motif: RRAANTGAAASYGNV | 0.0238 | 1023 | Ccl7/Fgl2/Ifit3/Mx1/Ifit2/Zfp36/Oas3/Ifi44 | 8/23 | 1023/19943 |
| **TF:M11679** | TF | TF:M11679 | Factor: IRF-7; motif: NCGAAANCGAAANYN | 0.0268 | 629 | Irf7/Ifit3/Mx1/Ifit2/Tap1/Oas3 | 6/18 | 629/19943 |
| **TF:M04015_1** | TF | TF:M04015_1 | Factor: IRF-4; motif: NCGAAACCGAAACYA; match class: 1 | 0.0275 | 167 | Irf7/Ifit2/Tap1/Oas3 | 4/18 | 167/19943 |
| **TF:M11680** | TF | TF:M11680 | Factor: IRF-9; motif: NYGAAACYGAAACYN | 0.0355 | 137 | Ifit3/Mx1/Ifit2/Ifi44 | 4/23 | 137/19943 |
| **TF:M11686** | TF | TF:M11686 | Factor: IRF-4; motif: NYGAAASYGAAACYN | 0.0472 | 3425 | Irf7/Ccl8/Zbp1/Atf5/Ifit3/Mx1/Il1b/Ifit2/Zfp36/ Tap1/Oas3/Nfkbiz/Ifi44 | 13/23 | 3425/19943 |
| **TF:M09733** | TF | TF:M09733 | Factor: STAT2; motif: NRGAAANNGAAACTNA | 0.0494 | 2890 | Irf7/Ccl8/Zbp1/Atf5/Ifit3/Mx1/Ifit2/Zfp36/Tap1/ Oas3/Nfkbiz/Ifi44 | 12/23 | 2890/19943 |
| **WP:WP5039** | WP | WP:WP5039 | SARS-CoV-2 innate immunity evasion and cell-specific immune response | 0.0005 | 66 | Ccl5/Irf7/Mx1/Ifit2 | 4/14 | 66/7827 |
| **WP:WP4655** | WP | WP:WP4655 | Cytosolic DNA-sensing pathway | 0.0005 | 73 | Ccl5/Irf7/Zbp1/Il1b | 4/13 | 73/7827 |
| **WP:WP619** | WP | WP:WP619 | Type II interferon signaling | 0.0006 | 37 | Il1b/Ifit2/Tap1/Socs3 | 4/25 | 37/7827 |
| **WP:WP4197** | WP | WP:WP4197 | Immune response to tuberculosis | 0.0014 | 23 | Ifit3/Mx1/Tap1 | 3/16 | 23/7827 |
| **WP:WP5095** | WP | WP:WP5095 | Overview of proinflammatory and profibrotic mediators | 0.0020 | 127 | Ccl7/Ccl5/Ccl8 | 3/4 | 127/7827 |
| **WP:WP5115** | WP | WP:WP5115 | Network map of SARS-CoV-2 signaling pathway | 0.0021 | 218 | Ccl5/Ccl8/Ccr5/Mx1/Il1b | 5/13 | 218/7827 |
| **WP:WP5088** | WP | WP:WP5088 | Prostaglandin signaling | 0.0091 | 33 | Irf7/Il1b/Ptger4 | 3/20 | 33/7827 |
| **WP:WP4630** | WP | WP:WP4630 | Measles virus infection | 0.0271 | 136 | Irf7/Mx1/Il1b/Oas3 | 4/18 | 136/7827 |
| **WP:WP4462** | WP | WP:WP4462 | Platelet-mediated interactions with vascular and circulating cells | 0.0419 | 17 | Ccl5/Il1b | 2/13 | 17/7827 |

**Table S2.3. Gene ontology enrichment analysis on the downregulated genes in IDE-KO microglia**

|  | **Category** | **ID** | **Description** | **P-adj.**  **value** | **Term Size** | **Gene ID** | **Gene**  **Ratio** | **Bg**  **Ratio** |
| --- | --- | --- | --- | --- | --- | --- | --- | --- |
| **GO:0022408** | GO:BP | GO:0022408 | negative regulation of cell-cell adhesion | 7.43e-05 | 192 | Gpnmb/Cd9/Il1rn/Cdkn2a/Anxa1 | 5/12 | 192/21092 |
| **GO:0030155** | GO:BP | GO:0030155 | regulation of cell adhesion | 7.49e-05 | 775 | Gpnmb/Cd9/Il1rn/Cdkn2a/Anxa1/Emilin2/Alox5/Tgm2/Cd28/Alox15 | 10/29 | 775/21092 |
| **GO:0022407** | GO:BP | GO:0022407 | regulation of cell-cell adhesion | 0.0001 | 475 | Gpnmb/Cd9/Il1rn/Cdkn2a/Anxa1/Emilin2/Alox5 | 7/18 | 475/21092 |
| **GO:0007162** | GO:BP | GO:0007162 | negative regulation of cell adhesion | 0.0005 | 287 | Gpnmb/Cd9/Il1rn/Cdkn2a/Anxa1 | 5/12 | 287/21092 |
| **GO:0061041** | GO:BP | GO:0061041 | regulation of wound healing | 0.0065 | 130 | Cd9/Anxa1/Emilin2/Alox5 | 4/18 | 130/21092 |
| **GO:0050866** | GO:BP | GO:0050866 | negative regulation of cell activation | 0.0081 | 215 | Gpnmb/Cd9/Cdkn2a/Anxa1 | 4/12 | 215/21092 |
| **GO:0098609** | GO:BP | GO:0098609 | cell-cell adhesion | 0.0122 | 946 | Gpnmb/Cd9/Il1rn/Cdkn2a/Anxa1/Emilin2/Alox5 | 7/18 | 946/21092 |
| **GO:1903034** | GO:BP | GO:1903034 | regulation of response to wounding | 0.0157 | 162 | Cd9/Anxa1/Emilin2/Alox5 | 4/18 | 162/21092 |
| **GO:2001301** | GO:BP | GO:2001301 | lipoxin biosynthetic process | 0.0182 | 4 | Alox5/Alox15 | 2/29 | 4/21092 |
| **GO:0007155** | GO:BP | GO:0007155 | cell adhesion | 0.0193 | 1521 | Gpnmb/Cd9/Il1rn/Cdkn2a/Anxa1/Emilin2/Alox5/Tgm2/Cd28/Alox15/Adgre5 | 11/33 | 1521/21092 |
| **GO:0033034** | GO:BP | GO:0033034 | positive regulation of myeloid cell apoptotic process | 0.0221 | 10 | Cdkn2a/Anxa1 | 2/12 | 10/21092 |
| **GO:2000026** | GO:BP | GO:2000026 | regulation of multicellular organismal development | 0.0227 | 1394 | Gpnmb/Cdkn2a/Lrrc17/Anxa1/Emilin2/Alox5/ Bhlhe40/Tgm2/Cd28/Dab2ip | 10/30 | 1394/21092 |
| **GO:0042129** | GO:BP | GO:0042129 | regulation of T cell proliferation | 0.0262 | 182 | Gpnmb/Cdkn2a/Anxa1/Cd28/Tnfsf8 | 5/37 | 182/21092 |
| **GO:0042060** | GO:BP | GO:0042060 | wound healing | 0.0425 | 441 | Cd9/Anxa1/Emilin2/Ahnak/Alox5/Alox15 | 6/29 | 441/21092 |
| **GO:1902106** | GO:BP | GO:1902106 | negative regulation of leukocyte differentiation | 0.0437 | 106 | Cdkn2a/Lrrc17/Anxa1 | 3/12 | 106/21092 |
| **GO:2001300** | GO:BP | GO:2001300 | lipoxin metabolic process | 0.0454 | 6 | Alox5/Alox15 | 2/29 | 6/21092 |
| **GO:1903707** | GO:BP | GO:1903707 | negative regulation of hemopoiesis | 0.0489 | 110 | Cdkn2a/Lrrc17/Anxa1 | 3/12 | 110/21092 |
| **GO:0071944** | GO:CC | GO:0071944 | cell periphery | 1.25e-06 | 6270 | Gpnmb/S100a6/Hgsnat/Ide/Cd9/Il1rn/Abcb4/ St3gal5/Lrrc17/Anxa1/Emilin2/S100a4/Atp8b4/ Ahnak/Pls3/Ralgapa2/Anpep/Timd4/Marveld1/ Tgm2/Cd28/Alox15/Dab2ip/Emp1/Adgre5/Slc7a2/Tnfsf8/Ldlrad3/L1cam | 29/39 | 6270/21690 |
| **GO:0005886** | GO:CC | GO:0005886 | plasma membrane | 4.70e-05 | 5788 | Gpnmb/S100a6/Hgsnat/Ide/Cd9/Il1rn/Abcb4/ St3gal5/Anxa1/Atp8b4/Ahnak/Pls3/Ralgapa2/ Anpep/Timd4/Marveld1/Tgm2/Cd28/Alox15/ Dab2ip/Emp1/Adgre5/Slc7a2/Tnfsf8/Ldlrad3/ L1cam | 26/39 | 5788/21690 |
| **GO:0031982** | GO:CC | GO:0031982 | vesicle | 4.95-05 | 3973 | Gpnmb/S100a6/Hgsnat/Ide/Cd9/Il1rn/Abcb4/ Anxa1/S100a4/Atp8b4/Pdxk/Ahnak/Alox5/Sgsh | 14/19 | 3973/21690 |
| **GO:0005615** | GO:CC | GO:0005615 | extracellular space | 0.0002 | 3368 | S100a6/Ide/Cd9/Il1rn/Abcb4/Lrrc17/Anxa1/ S100a4/Pdxk/Ahnak/Alox5/Sgsh/Ralgapa2/Anpep | 14/23 | 3368/21690 |
| **GO:0005576** | GO:CC | GO:0005576 | extracellular region | 0.0002 | 4302 | S100a6/Ide/Cd9/Il1rn/Abcb4/Lrrc17/Anxa1/ Emilin2/S100a4/Pdxk/Ahnak/Alox5/Sgsh/ Ralgapa2/Anpep/Timd4/Tgm2 | 17/27 | 4302/21690 |
| **GO:0070062** | GO:CC | GO:0070062 | extracellular exosome | 0.0006 | 2108 | S100a6/Ide/Cd9/Il1rn/Abcb4/Anxa1/S100a4/Pdxk/Ahnak/Sgsh/Anpep/Tgm2 | 12/27 | 2108/21690 |
| **GO:1903561** | GO:CC | GO:1903561 | extracellular vesicle | 0.0007 | 2132 | S100a6/Ide/Cd9/Il1rn/Abcb4/Anxa1/S100a4/Pdxk/Ahnak/Sgsh | 10/19 | 2132/21690 |
| **GO:0065010** | GO:CC | GO:0065010 | extracellular membrane-bounded organelle | 0.0007 | 2133 | S100a6/Ide/Cd9/Il1rn/Abcb4/Anxa1/S100a4/Pdxk/Ahnak/Sgsh | 10/19 | 2133/21690 |
| **GO:0043230** | GO:CC | GO:0043230 | extracellular organelle | 0.0007 | 2133 | S100a6/Ide/Cd9/Il1rn/Abcb4/Anxa1/S100a4/Pdxk/Ahnak/Sgsh | 10/19 | 2133/21690 |
| **GO:0005925** | GO:CC | GO:0005925 | focal adhesion | 0.0021 | 424 | Cd9/Abcb4/Anxa1/Ahnak/Tgm2/Adgre5/L1cam | 7/39 | 424/21690 |
| **GO:0030055** | GO:CC | GO:0030055 | cell-substrate junction | 0.0024 | 433 | Cd9/Abcb4/Anxa1/Ahnak/Tgm2/Adgre5/L1cam | 7/39 | 433/21690 |
| **GO:0031012** | GO:CC | GO:0031012 | extracellular matrix | 0.0044 | 565 | S100a6/Lrrc17/Anxa1/Emilin2/S100a4 | 5/14 | 565/21690 |
| **GO:0030312** | GO:CC | GO:0030312 | external encapsulating structure | 0.0045 | 566 | S100a6/Lrrc17/Anxa1/Emilin2/S100a4 | 5/14 | 566/21690 |
| **GO:0098552** | GO:CC | GO:0098552 | side of membrane | 0.0057 | 681 | S100a6/Ide/Cd9/Anxa1/Anpep/Cd28/Alox15 | 7/29 | 681/21690 |
| **GO:0016020** | GO:CC | GO:0016020 | membrane | 0.0082 | 9856 | Gpnmb/S100a6/Hgsnat/Ide/Cd9/Il1rn/Abcb4/ St3gal5/Rraga/Anxa1/Atp8b4/Ahnak/Alox5/Pls3/Ralgapa2/Anpep/Chchd10/Timd4/Marveld1/ Tgm2/Cd28/Alox15/Dab2ip/Emp1/Adgre5/ Gask1b/Slc7a2/Tnfsf8/Ldlrad3/L1cam/Tmem158 | 31/40 | 9856/21690 |
| **GO:0005737** | GO:CC | GO:0005737 | cytoplasm | 0.0182 | 12270 | Gpnmb/S100a6/Hgsnat/Ide/Cd9/Il1rn/Cdkn2a/ Abcb4/St3gal5/Rraga/Anxa1/S100a4/Atp8b4/ Pdxk/Ahnak/Alox5/Sgsh/Pls3/Bhlhe40/Ralgapa2/Anpep/Chchd10/Marveld1/Tgm2/Cd28/Alox15/ Dab2ip | 27/30 | 12270/21690 |
| **GO:0098588** | GO:CC | GO:0098588 | bounding membrane of organelle | 0.0213 | 2132 | Gpnmb/Hgsnat/Cd9/St3gal5/Rraga/Anxa1/ Atp8b4/Ahnak | 8/17 | 2132/21690 |
| **GO:0030667** | GO:CC | GO:0030667 | secretory granule membrane | 0.0223 | 309 | Hgsnat/Cd9/Atp8b4/Anpep/Adgre5 | 5/33 | 309/21690 |
| **GO:0062023** | GO:CC | GO:0062023 | collagen-containing extracellular matrix | 0.0249 | 429 | S100a6/Anxa1/Emilin2/S100a4/Tgm2/L1cam | 6/39 | 429/21690 |
| **GO:0042581** | GO:CC | GO:0042581 | specific granule | 0.0456 | 158 | Hgsnat/Atp8b4/Pdxk | 3/16 | 158/21690 |
| **GO:0004052** | GO:MF | GO:0004052 | arachidonate 12(S)-lipoxygenase activity | 0.0045 | 4 | Alox5/Alox15 | 2/29 | 4/20166 |
| **GO:0048306** | GO:MF | GO:0048306 | calcium-dependent protein binding | 0.0084 | 81 | S100a6/Anxa1/S100a4 | 3/14 | 81/20166 |
| **GO:0097493** | GO:MF | GO:0097493 | structural molecule activity conferring elasticity | 0.0139 | 11 | Emilin2/Ahnak | 2/17 | 11/20166 |
| **GO:0044548** | GO:MF | GO:0044548 | S100 protein binding | 0.0230 | 14 | S100a6/Ahnak | 2/17 | 14/20166 |
| **HPA:0300413** | HPA | HPA:0300413 | lung; macrophages[High] | 0.0362 | 1357 | S100a6/Hgsnat/Cd9/Rraga/Anxa1/S100a4/ Atp8b4/Alox5/Sgsh | 9/19 | 1357/10976 |
| **REAC:R-HSA-9026286** | REAC | REAC:R-HSA-9026286 | Biosynthesis of DPAn-3-derived protectins and resolvins | 0.0022 | 2 | Alox5/Alox15 | 2/29 | 2/10461 |
| **REAC:R-HSA-9023661** | REAC | REAC:R-HSA-9023661 | Biosynthesis of E-series 18(R)-resolvins | 0.0067 | 3 | Alox5/Alox15 | 2/29 | 3/10461 |
| **REAC:R-HSA-9018683** | REAC | REAC:R-HSA-9018683 | Biosynthesis of DPA-derived SPMs | 0.0134 | 4 | Alox5/Alox15 | 2/29 | 4/10461 |
| **REAC:R-HSA-9018896** | REAC | REAC:R-HSA-9018896 | Biosynthesis of E-series 18(S)-resolvins | 0.0134 | 4 | Alox5/Alox15 | 2/29 | 4/10461 |
| **REAC:R-HSA-9025094** | REAC | REAC:R-HSA-9025094 | Biosynthesis of DPAn-3 SPMs | 0.0134 | 4 | Alox5/Alox15 | 2/29 | 4/10461 |
| **REAC:R-HSA-9018679** | REAC | REAC:R-HSA-9018679 | Biosynthesis of EPA-derived SPMs | 0.0223 | 5 | Alox5/Alox15 | 2/29 | 5/10461 |
| **TF:M11728** | TF | TF:M11728 | Factor: NF-1C; motif: NTTGGCNNNNTGCCARN | 0.0450 | 2636 | Gpnmb/S100a6/Hgsnat/Ide/Cd9/Abcb4/Lrrc17/ Ahnak/Sgsh/Pls3/Bhlhe40 | 11/21 | 2636/19943 |
| **WP:WP5137** | WP | WP:WP5137 | Linoleic acid oxylipin metabolism | 0.0044 | 3 | Alox5/Alox15 | 2/29 | 3/7827 |
| **WP:WP5136** | WP | WP:WP5136 | ALA oxylipin metabolism | 0.0044 | 3 | Alox5/Alox15 | 2/29 | 3/7827 |
| **WP:WP2877** | WP | WP:WP2877 | Vitamin D receptor pathway | 0.0048 | 182 | S100a6/Cd9/Cdkn2a/S100a4/Alox5 | 5/18 | 182/7827 |
| **WP:WP4586** | WP | WP:WP4586 | Metabolism of alpha-linolenic acid | 0.0219 | 6 | Alox5/Alox15 | 2/29 | 6/7827 |
