# Supplementary material for: Insulin-degrading enzyme (IDE) as a modulator of microglial phenotypes in the context of Alzheimer’s disease and brain aging
Source: J Neuroinflammation. 2023 Oct 11;20:233. doi: 10.1186/s12974-023-02914-7 (PMC10566021; doi:10.1186/s12974-023-02914-7)
Supplement: Supplementary file 3 — Additional file 3: File S3. Cytokine profiling results of IDE-KO vs. WT primary microglia. [file 12974_2023_2914_MOESM3_ESM.docx]

**Insulin-degrading enzyme (IDE) as a modulator of microglial phenotypes in the context of Alzheimer’s disease and brain aging.**

**Miriam Corraliza-Gomez, Teresa Bermejo, Jingtao Lilue, Noelia Rodriguez-Iglesias, Jorge Valero, Irene Cozar-Castellano, Eduardo Arranz, Diego Sanchez and Maria Dolores Ganfornina**

**Supplementary File 3.** **Cytokine profiling results of IDE-KO vs WT primary microglia.**

**Table S3. Cytokine profiling in WT and IDE-KO primary microglia subjected to different stimuli.** *Data are expressed as mean ± SEM between three biological samples. Statistical differences were analyzed individually in each sex by 2-way ANOVA considering the factors “Genotype” and “Treatment”. Post-hoc pairwise comparisons were performed using Holm-Sidak tests. Red asterisks indicate significant genotype-dependent differences.*

| **Male microglia** | | | | | | | | | | |
| --- | --- | --- | --- | --- | --- | --- | --- | --- | --- | --- |
|  | **Control** | | **LPS** | | **IL-4 + IL-13** | | **PQ** | | **Aβ oligomers** | |
|  | **WT** | **KO** | **WT** | **KO** | **WT** | **KO** | **WT** | **KO** | **WT** | **KO** |
| **TNF-α** | <0.2 | <0.2 | 308.9±52.0 | 246.2±14.6 * | 0.70±0.69 | 4.6±0.8 | 0.19±0.0 | 0.94±0.0 | <0.2 | 7.4±1.0 * |
| **IL-1β** | <23.0 | <23.0 | 207.7±13.6 | 226.4±36.1 | <23.0 | <23.0 | <23.0 | <23.0 | <23.0 | <23.0 |
| **IL-6** | <2.5 | <2.5 | 530.0±85.7 | 595.2±75.6 | 0.3±0.1 | 1.8±0.7 | <2.5 | <2.5 | <2.5 | 4.2±1.3 |
| **IL-4** | <6.1 | <6.1 | <6.1 | <6.1 | 299.4±37.9 | 202.3±21.5 * | <6.1 | <6.1 | <6.1 | <6.1 |
| **IL-10** | <1.4 | <1.4 | 8.2±1.0 | 7.4±1.7 * | 0.3±0.1 | 0.3±0.1 | <1.4 | <1.4 | <1.4 | <1.4 |
| **TGF-β** | 54.7±9.3 | 60.1±11.1 | 75.5±4.3 | 77.0±2.6 | 77.0±12.2 | 78.3±6.7 | 29.0±3.5 | 13.8±1.1 * | 75.5±4.3 | 81.0±4.6 |
| **Female microglia** | | | | | | | | | | |
|  | **Control** | | **LPS** | | **IL-4 + IL-13** | | **PQ** | | **Aβ oligomers** | |
|  | **WT** | **KO** | **WT** | **KO** | **WT** | **KO** | **WT** | **KO** | **WT** | **KO** |
| **TNF-α** | <0.2 | <0.2 | 269.5±19.1 | 235.2±27.4 * | <0.2 | 2.4±1.0 | 0.2±0.1 | 0.5±0.1 | <0.2 | 0.5±0.1 |
| **IL-1β** | <23.0 | <23.0 | 165.1±18.8 | 198.6±84.6 | <23.0 | <23.0 | <23.0 | <23.0 | <23.0 | <23.0 |
| **IL-6** | <2.5 | <2.5 | 609.5±67.0 | 640.4±97.0 | 0.1±0.0 | 0.5±50.2 | <2.5 | <2.5 | <2.5 | <2.5 |
| **IL-4** | <6.1 | <6.1 | <6.1 | <6.1 | 354.0±14.2 | 252.4±27.6 * | <6.1 | 3.5±1.2 | <6.1 | <6.1 |
| **IL-10** | <1.4 | <1.4 | 5.7±0.5 | 6.8±0.1 * | 0.1±0.0 | 0.1±0.0 | <1.4 | 2.7±0.9 | <1.4 | <1.4 |
| **TGF-β** | 45.4±9.1 | 55.0±2.8 | 87.9±2.5 | 82.9±5.3 | 39.4±22.7 | 78.3±4.3 | 33.0±7.6 | 7.5±4.4 * | 89.9±5.0 | 96.6±4.8 |


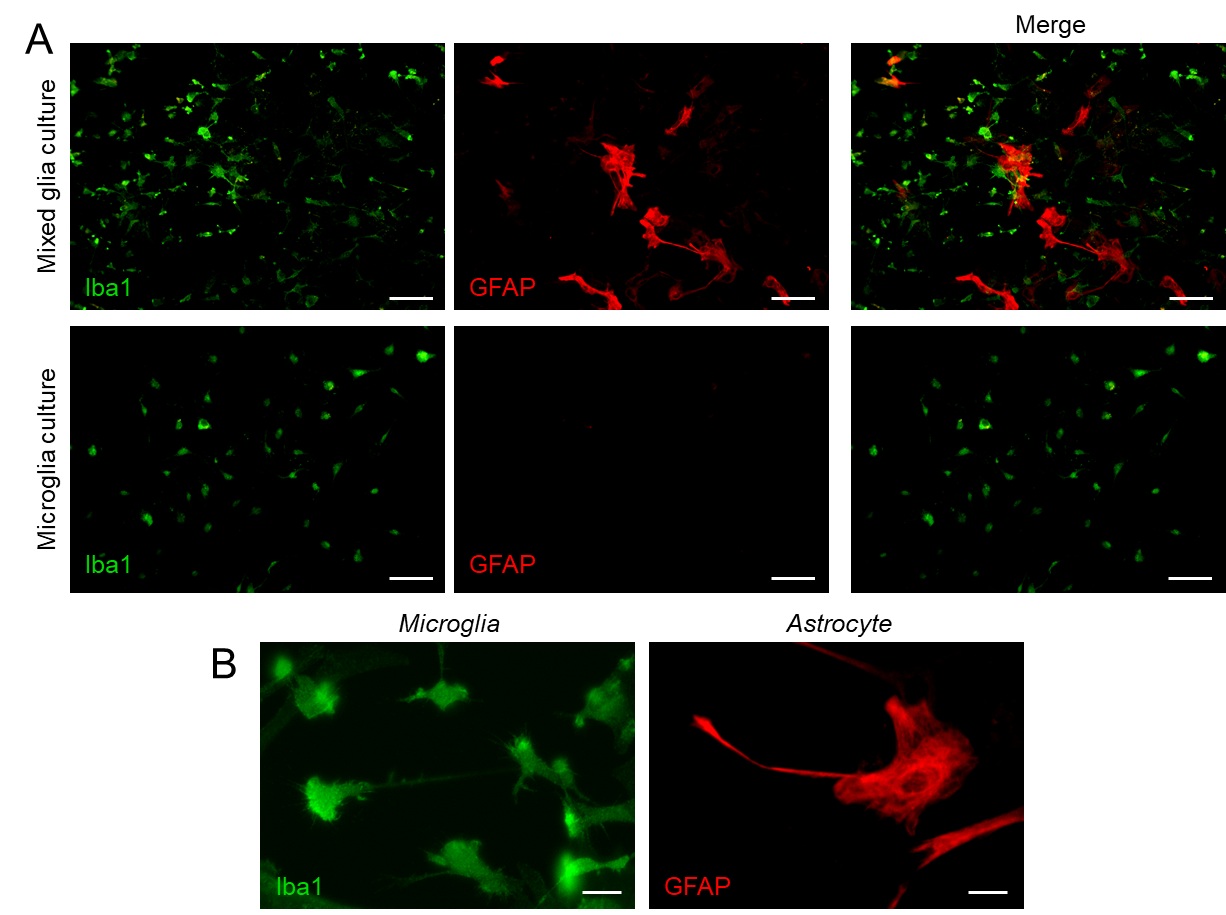


**Figure S3.1. Immunocytochemistry on primary mixed and pure microglia cultures.** Samples were labelled with Iba1 and GFAP. Only pure microglial cultures were used in all our functional experiments. Wells with astrocytes remaining after the mild trypsinization step were discarded. Calibration bars: 50 µm (A), 10 µm (B).
